# Pleiotropic genetic influence on birth weight and childhood obesity

**DOI:** 10.1038/s41598-020-80084-9

**Published:** 2021-01-08

**Authors:** Suvo Chatterjee, Marion Ouidir, Fasil Tekola-Ayele

**Affiliations:** Epidemiology Branch, Division of Intramural Population Health Research, Eunice Kennedy Shriver National Institute of Child Health and Human Development, National Institutes of Health, 6710B Rockledge Drive, Room 3204, Bethesda, 20892-7004 USA

**Keywords:** Genetics, Risk factors

## Abstract

Childhood obesity is a global public health problem. Understanding the molecular mechanisms that underlie early origins of childhood obesity can facilitate interventions. Consistent phenotypic and genetic correlations have been found between childhood obesity traits and birth weight (a proxy for in-utero growth), suggesting shared genetic influences (pleiotropy). We aimed to (1) investigate whether there is significant shared genetic influence between birth weight and childhood obesity traits, and (2) to identify genetic loci with shared effects. Using a statistical approach that integrates summary statistics and functional annotations for paired traits, we found strong evidence of pleiotropy (*P* < 3.53 × 10^–127^) and enrichment of functional annotations (*P* < 1.62 × 10^–39^) between birth weight and childhood body mass index (BMI)/obesity. The pleiotropic loci were enriched for regulatory features in skeletal muscle, adipose and brain tissues and in cell lines derived from blood lymphocytes. At 5% false discovery rate, 6 loci were associated with birth weight and childhood BMI and 13 loci were associated with birth weight and childhood obesity. Out of these 19 loci, one locus (*EBF1*) was novel to childhood obesity and one locus (*LMBR1L*) was novel to both birth weight and childhood BMI/obesity. These findings give evidence of substantial shared genetic effects in the regulation of both fetal growth and childhood obesity.

## Introduction

Childhood overweight or obesity is a global public health problem^1–3^. Complex interactions of genetic susceptibility^4–6^, environmental exposures^7,8^ and *in-utero* programming^9–12^ contribute to childhood obesity. Fetal growth and development during the intrauterine period shape physiological and structural processes that impact risk for childhood obesity (COB)^9^. Birth weight, a marker of fetal growth and prenatal environment, has been consistently associated with COB^13–20^. Notably, a significant positive genetic correlation has been found between birth weight and COB/childhood body mass index (CBMI)^21^, suggesting the possibility for genetic links these phenotypes may share. Insight into the shared genetic underpinnings of these early life phenotypes facilitates knowledge on the potential common molecular pathways and intervention targets of abnormal birth weight and COB/CBMI. However, pleiotropic evidence on these traits remains scarce.

Recent genome wide association studies (GWAS) have reported that shared genetic effects (pleiotropy) might explain a substantial proportion of correlations between complex human traits^22–26^. In an analysis of the GWAS catalog^27^ it was found that 16.9% of the reported genes were associated with multiple traits^28^. With the advent of large scale independent GWASs on multiple early life traits and functional annotation database^29–31^, innovative statistical approaches can be applied to identify and test for novel pleiotropic loci.

In addition, accounting for shared genetic effects improves statistical power for detecting genetic variants that are associated with complex traits^32^. Both birth weight and COB/CBMI are under strong genetic control^10,11,33–35^, with relatively high heritability estimates of 25–40% for birth weight^35^ and 30–70% for COB/CBMI^34^. However, the heritability of the traits explained by genetic variants identified using published GWAS for birth weight^36^, COB^37^ and CBMI^38–40^ was less than 10%. Therefore, detecting novel genetic loci via pleiotropic analysis can help close the gap in missing heritability.

To date, pleiotropic associations of birth weight and COB/CBMI have not been exclusively investigated. In the current study, we used an innovative statistical approach that integrates pleiotropy and functional annotation data with the following aims: (1) to investigate whether there is significant shared genetic influence between birth weight and COB/CBMI (pleiotropy enrichment test), (2) to examine whether the shared risk variants are functionally more enriched in birth weight and COB/CBMI as compared to only considering single traits in models that incorporate a variety of genetic annotations in different tissues and cell types (annotation enrichment test), and (3) to identify genetic loci with shared genetic effects on birth weight and COB/CBMI. Our analysis found abundant evidence of pleiotropy and significant enrichment of functional annotations for shared risk variants associated with birth weight and COB/CBMI. We identified genetic loci with overlapping influence on both birth weight and COB/CBMI. Most of those loci have been associated with childhood BMI/obesity in previous GWAS but not with birth weight, whereas one locus (*EBF1*) was novel to childhood obesity and one locus (*LMBR1L*) was novel to both birth weight and childhood BMI/obesity.

## Results

Data on summary statistics including *P* values and direction of effects in GWAS meta-analysis of birth weight, CBMI and COB was obtained from publicly available data of the Early Growth Genetics (EGG) consortium. Two sets of GWAS summary statistics were available for birth weight, one involving European ancestry individuals (BW_EU_) and a second from trans-ancestry meta-analysis (BW_TR_). The GWAS summary statistics for CBMI and COB were from European ancestry individuals. Therefore, tests of pleiotropy were conducted on four trait pairs: BW_EU_-CBMI, BW_EU_-COB, BW_TR_-CBMI, and BW_TR_-COB.

Pleiotropy analysis was performed via the R package GPA^41^ which implements a statistical approach to explore the genetic architecture of complex traits by integrating pleiotropy and functional annotation information, including prioritizing risk genetic variants. GPA performs hypothesis test for evaluating enrichment of pleiotropy and functional annotations using a likelihood ratio test approach. For each trait pair, we performed tests of pleiotropy and enrichment of functional annotations based on 4 functional categories: combined annotation dependent depletion (CADD) (http://www.cadd.gs.washington.edu)^42^, expression quantitative trait loci (eQTL) (https://gtexportal.org/home/datasets)^43^, transcription factor binding sites (TFBS) (ftp://ccg.epfl.ch/snp2tfbs/mapped_files/annotated)^44^, and DNase I hypersensitivity sites (DHS) (https://github.com/joepickrell/1000-genomes)^45^.

### Evidence of pleiotropy between birth weight and childhood obesity traits

Under each functional category, we found evidence of significant pleiotropic genetic effects for all tested trait pairs. Pleiotropic genetic effects had enrichment fold ranging from 2.6 to 5.4 (1.17 × 10^–277^ < *P* < 3.51 × 10^–127^) under CADD annotation, 2.6 to 5.2 (1.18 × 10^–277^ < *P* < 3.51 × 10^–127^) under eQTL annotation, 2.6 to 5.1 (2.52 × 10^–276^ < *P* < 6.52 × 10^–127^) under DHS annotation, and 2.6 to 5.1 (1.18 × 10^–277^ < *P* < 6.52 × 10^–127^) under TFBS annotation (Table 1).

**Table 1:** Genetic pleiotropy and enrichment of functional deleteriousness among genetic loci associated with birth weight and childhood obesity traits.

Trait pair	Annotation	Genetic pleiotropy		Functional annotation enrichment	
		Enrichment fold (s.e)	*P* value	q11/q00 (s.e)	*P* value
BW_EU_ and CBMI	CADD	2.683 (0.05)	1.17 × 10^–277^	1.69 (0.08)	2.69 × 10^–38^
	eQTLs	2.679 (0.05)	1.18 × 10^–277^	2.10 (0.04)	≤ 1 × 10^–300^
	DHSs	2.67 (0.05)	2.52 × 10^–276^	1.17 (0.01)	≤ 1 × 10^–300^
	TFBS	2.685 (0.05)	1.18 × 10^–277^	1.09 (0.03)	0.03
BW_EU_ and COB	CADD	5.413 (0.19)	9.79 × 10^–144^	1.54 (0.18)	1.42 × 10^–27^
	eQTLs	5.396 (0.29)	9.79 × 10^–144^	1.73 (0.09)	≤ 1 × 10^–300^
	DHSs	5.379 (0.24)	1.45 × 10^–143^	1.15 (0.03)	≤ 1 × 10^–300^
	TFBS	5.383 (0.24)	9.79 × 10^–144^	1.01 (0.06)	0.21
BW_TR_ and CBMI	CADD	2.653 (0.05)	9.97 × 10^–274^	1.79 (0.08)	1.62 × 10^–39^
	eQTLs	2.648 (0.05)	9.98 × 10^–271^	2.15 (0.04)	≤ 1 × 10^–300^
	DHSs	2.641 (0.05)	2.18 × 10^–269^	1.17 (0.02)	≤ 1 × 10^–300^
	TFBS	2.655 (0.05)	9.98 × 10^–271^	1.10 (0.03)	0.01
BW_TR_ and COB	CADD	5.209 (0.17)	3.51 × 10^–127^	1.68 (0.18)	1.94 × 10^–29^
	eQTLs	5.204 (0.18)	3.51 × 10^–127^	1.80 (0.10)	≤ 1 × 10^–300^
	DHSs	5.186 (0.20)	6.52 × 10^–127^	1.12 (0.03)	≤ 1 × 10^–300^
	TFBS	5.197 (0.18)	3.51 × 10^–127^	0.99 (0.07)	0.04

*q*11/*q*00 is the ratio of the probability of jointly associated SNPs being functionally annotated to the probability of a null SNP (not associated with either trait) being functionally annotated.

*BW*_*EU*_* and CBMI* European birth weight and childhood body mass index, *BW*_*EU*_* and COB* European birth weight and childhood obesity, *BW*_*TR*_* and CBMI* trans-ethnic birth weight and childhood body mass index, *BW*_*TR*_* and COB* trans-ethnic birth weight and childhood obesity, *CADD* combined annotation dependent depletion, *eQTLs* expression quantitative loci, *DHSs* DNase I hypersensitivity sites, *TFBS* transcription factor binding sites.

### Enrichment of functional annotations

Next, we assessed whether genetic loci with regulatory functional annotations had higher likelihood of being associated with both birth weight and CBMI/COB as compared to genetic loci without functional annotations. Functional annotation of the 2.4 million single-nucleotide polymorphisms (SNPs) tested was done using CADD^42^, eQTL^43^, TFBS^44^ , and DHS^45^. Out of the ~ 2.4 million SNPs tested, approximately 2% of the SNPs were annotated as CADD related, 7% as eQTL related, 20% as TFBS related and 51% as DHS related (Table S1). Enrichment of each of the four functional categories was tested for four trait pairs using the GPA model^41^, resulting in 16 tests of functional enrichment.

**Figure 1:**
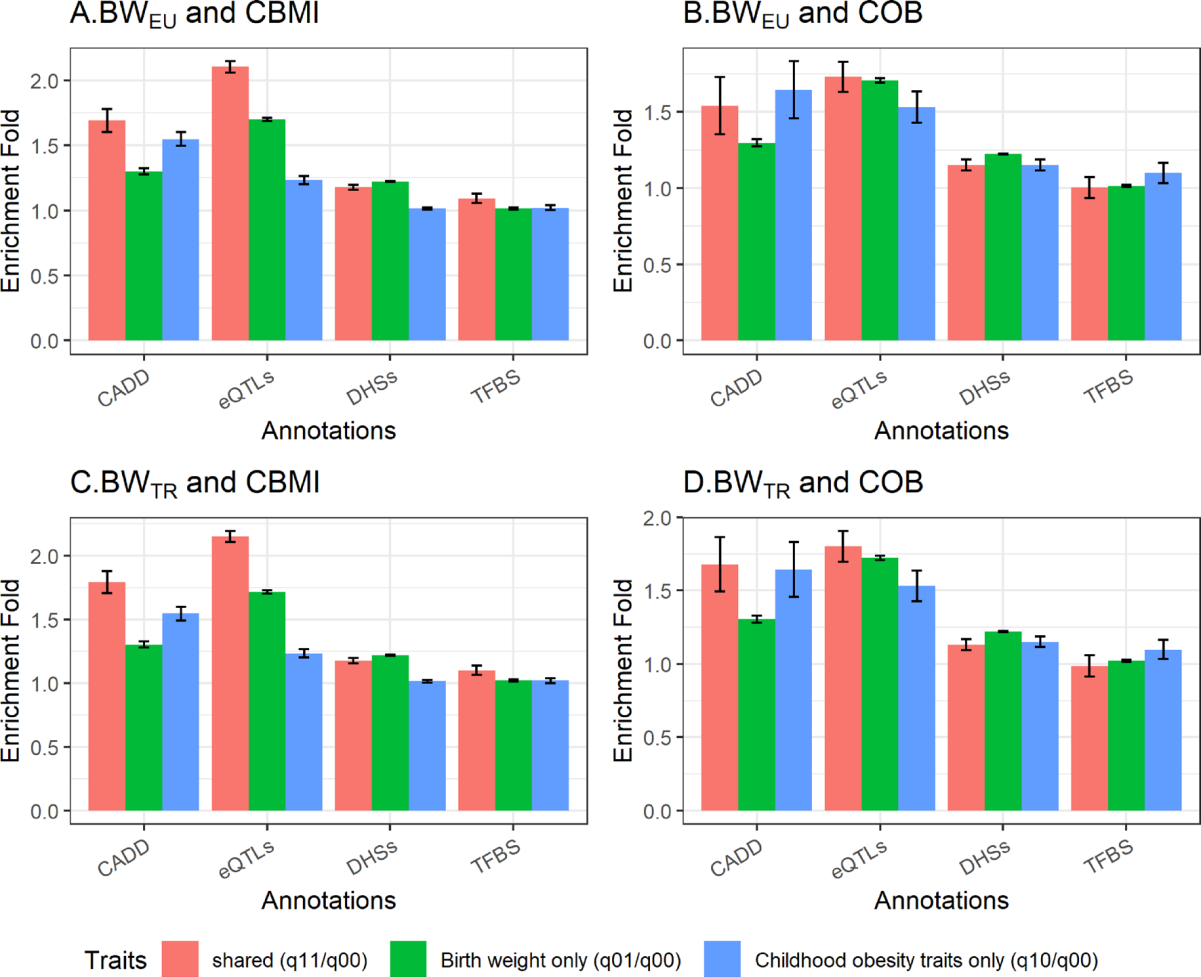
Enrichment of four functional annotations among loci jointly associated with birth weight and childhood obesity traits. (**A**) European birth weight and childhood body mass index (BW_EU_-CBMI), (**B**) European birth weight and childhood obesity (BW_EU_-COB), (**C**) Trans-ethnic birth weight and childhood body mass index (BW_TR_-CBMI), (**D**) Trans-ethnic birth weight and childhood obesity (BW_TR_-COB). The bars denote the enrichment fold for variants that are associated jointly with BW and childhood obesity traits (red), only with birth weight (green), and only with childhood obesity traits (blue), respectively.

To compare the functional deleteriousness of the pleiotropic SNPs with non-pleiotropic SNPs, each variant in our analysis was annotated to be deleterious or non-deleterious based on CADD score ≥ 15 or CADD score < 15, respectively^42^. Out of the 16 tests, SNPs associated with pairs of birth weight and childhood obesity traits were more likely to be functionally deleterious than SNPs associated with neither trait in 15 tests (enrichment fold ranging from 0.99 to 2.15 and Bonferroni corrected *P* values ranging from 1 × 10^–300^ ≤ *P* < 1.62 × 10^–39^); however, 1 test under TFBS category for BW_EU_-COB pair was not significant (*P* = 0.21) (Table 1). Enrichment of eQTL annotation was consistently higher for SNPs associated with both birth weight and COB traits compared to SNPs associated with only birth weight or CBMI/COB (Fig. 1).

We further examined the specific tissues, cell types and transcription factors that had relatively higher functional enrichment among the common variants between birth weight and childhood obesity traits. For each trait pair, we performed annotation enrichment tests of 49 tissues with eQTL annotations^43^, 402 tissues/cell lines/cell types with DHSs annotations^45^, and 195 transcription factors in the TFBS database^44^, one at a time. The most significant eQTL enrichments (*P* < 0.05 and lowest in the joint trait association) were observed in tissues from skeletal muscle, adipose, brain, heart, esophagus, thyroid, adrenal, colon, small intestine and whole blood. The most significant DHS enrichments were observed in cell lines derived from blood lymphocytes (e.g. CD4+), skin (e.g. iPS), cancer (e.g. HeLa), embryo (e.g. embryonic stem cells) and in tissues from fetal brain. The most significant transcription factor enrichments were Interferon Regulatory Factor 1, AT-Rich Interaction Domain 3A and Testicular receptor 4 (Table S2–S3).

**Figure 2:**
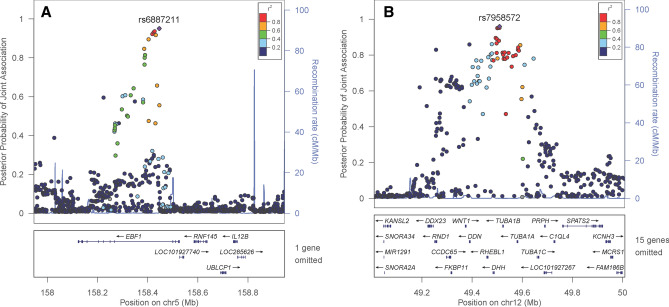
Regional plots of novel pleiotropic loci associated with birthweight and childhood obesity traits. (**A**) rs6887211 (*EBF1*) locus association with both birth weight and childhood body mass index (**B**) rs7958572 (*LMRB1L*) locus associated with both birthweight and childhood obesity or childhood body mass index. The horizontal axes cover a region 500 kb upstream and downstream from the reference SNP. The vertical axes denote the joint association probabilities of the SNPs with both birthweight and childhood obesity traits. The purple triangles denote the index SNPs (rs6887211 in *EBF1* and rs7958572 in *LMRB1L*). All other colored points denote the surrounding SNPs in that region, and they are colored based on their linkage disequilibrium (r^2^) with the reference SNP. The box at the bottom shows genes that fall in the region.

At 5% false discovery rate, 19 loci (consisting 509 SNPs, with each locus having correlated SNPs with linkage disequilibrium r^2^ > 0.5) were jointly associated with birth weight and childhood obesity traits (Figs. S1–S4). Among these 19 loci, 5 loci were jointly associated with European BW and COB traits, 1 locus was jointly associated with transethnic BW and COB traits and 13 loci were jointly associated with both European and transethnic BW and COB traits (Table S4–S5). Additionally, out of these 19 loci, 17 loci were novel to birth weight but not childhood obesity traits at the genome-wide significance level (*P* < 5 × 10^–8^, in the NHGRI-EBI GWAS catalogue: www.ebi.ac.uk/gwas/), one locus (rs6887211 in *EBF1*) was novel to childhood obesity traits but not birth weight, and one locus (rs7958572 4 Kb upstream to *LMBR1L*) was novel to both birth weight and childhood obesity traits (Fig. 2). The *EBF1* locus was only suggestively associated with childhood obesity traits (*P* = 8.44 × 10^–5^) and the *LMBR1L* locus was only suggestively associated with birth weight (*P* = 2.4 × 10^–3^) and childhood obesity traits (*P* = 7.3 × 10^–6^) in previous GWAS^36,39^.

For most of the jointly associated loci, the alleles associated with decreased birth weight were associated with lower CBMI/COB risk. In contrast, for three loci (*EBF1*, *NCOA1* and *SEC16B)*, nearly all alleles associated with decreased birth weight were associated with higher CBMI/COB risk (Table S4–S5). Furthermore, we evaluated our findings using the European-ancestry summary statistics in a recent childhood obesity GWAS^46^. Out of the 342 SNPs in our study found to be associated with both birthweight and childhood obesity, 98.5% reached > 95% posterior probability when we used this validation childhood obesity GWAS (Table S6).

### Pathway analysis

To further understand the functional relevance of the genetic loci jointly associated with birth weight and childhood obesity traits, we performed pathway enrichment analysis of the 25 genes that are near the pleiotropic SNPs using the Ingenuity Pathway Analysis (IPA) tool (QIAGEN Inc, https://digitalinsights.qiagen.com/). The top enriched IPA Canonical Pathways included RAR Activation and Estrogen Receptor Signaling (Table S7) and the top enriched IPA Disease or Function annotations included brain size and morphology (Table S8).

### Colocalization analysis

To determine whether the signals identified represent horizontal pleiotropy (influence birthweight and childhood obesity via independent biological pathways) or vertical pleiotropy (influence via shared mechanism in a causal pathway) we performed colocalization analysis using the coloc R package^47^. Only the *EBF1* locus showed evidence of colocalization with a posterior probability of 0.87 for being associated with both traits and share a single causal variant (Tables S9 and S10).

## Discussion

There is growing evidence that several genetic variants can influence two or more traits^28,48,49^. Investigating such effects in early life traits can facilitate our understanding of their complex genetic architecture and in developing early life interventions to promote long-term health^50,51^. The present study revealed substantial genetic pleiotropic effects between birth weight and childhood obesity traits. We also found that biologically functional SNPs are more likely to be associated with both birth weight and COB traits compared to SNPs that are not functional, consistent with evidence of high evolutionary conservation of pleiotropic genes and their consequences^52^. Lastly, we identified 19 genetic loci with pleiotropic effects, including loci in *EBF1* and *LMRBL1* that have not been associated with birth weight and/or COB phenotypes in previous GWAS. With the majority (68.4%) of the 19 loci reaching the significance threshold in analyses involving European-only or trans-ethnic birthweight GWAS and the remaining loci exhibiting high posterior probability of association (0.85–0.93), the results suggest that the effects of many pleiotropic loci are likely shared across ancestries. In all, these findings facilitate our understanding of the genetic mechanism that may underlie associations of early life growth with COB traits.

The birth weight-decreasing allele of the novel pleiotropic SNP rs6887211 (*EBF1*) was found to be associated with increased risk of COB traits. The association of *EBF1* with birth weight and gestational duration is well-recognized^36,53–55^. *EBF1* is highly expressed in adipocytes (Fig. S5) and plays a crucial role in adipogenesis and development of B lymphocytes. Dysregulated expression of *EBF1* is associated with adipose hypertrophy, adipose inflammation, variation in body fat distribution and altered adipose morphology through impaired adipogenesis, which in turn has been implicated as a key factor in the development of obesity related traits^56–59^. *EBF1* has a well-known association with cardiometabolic traits in adults. SNPs near *EBF1* have been associated with blood pressure^60^ and hip circumference^61^. Notably, a multi-ethnic GWAS in adults has found an association between rs1650505 (near *EBF1*) and pericardial adipose tissue volume^56^. Previous studies found substantial overlaps between childhood and adult obesity GWAS loci^62^. While there is relatively weak LD between the lead SNP showing pleiotropic effect in our study (rs6887211) and rs1650505 (r^2^ = 0.06 in CEU), we acknowledge that the novelty of the locus in childhood BMI may be in part due to the small study power of the source GWAS used for childhood BMI. Furthermore, our novel SNP and its LD proxies (r^2^ ≥ 0.8) overlapped with DNase I hypersensitivity sites of blood lymphocytes and fetal muscle further indicating that many components of the metabolic and inflammatory pathways are positively and directly regulated by *EBF1* as emphasized in a prior study^63^. These findings collectively indicate *EBF1*’s role in fetal growth and general adiposity which highlights that it should be considered as a key player in understanding the association of early life growth with the development of COB.

The birth weight-decreasing allele of the novel pleiotropic SNP in *LMBR1L* (rs7958572) was found to be associated with decreased risk of COB. *LMBR1L* has regulatory effects on the canonical Wnt signaling pathway. It stabilizes the beta-catenin destruction complex^64^ that is required for regulating *CTNNB1* expression, and is responsible in making the beta-catenin protein which plays a key role in the canonical Wnt signaling pathway^65–68^. The Wnt signaling pathway is conserved in various organisms and plays important roles in development, cellular proliferation, and differentiation^69^. Previous studies have demonstrated that the Wnt signaling pathway regulates adipogenesis and maintains the undifferentiated state of pre-adipocytes by inhibiting adipogenic gene expression ^70,71^. In addition, the Wnt signaling cascade plays fundamental roles in placental development by regulating trophoblast differentiation and invasion, and aberrant Wnt signaling activation can have downstream consequences on fetal growth ^72–74^. Our finding here for *LMBR1L*, together with the regulatory impact of *LMBR1L* on Wnt signaling and the functional relevance of the Wnt pathway in placental function, suggests a novel hypothetical mechanism by which *LMBR1L* contributes to the link between fetal growth and development of COB. Future studies such as eQTLs in tissues relevant to early life traits can facilitate better inference from the pleiotropic SNP to a potential functional gene.

In agreement with previous observational studies which reported that small for gestational age (SGA) or low birth weight is associated with both lower and higher risk of COB^13–20^, we observed that birth weight-decreasing alleles of pleiotropic SNPs may be associated with either increased or decreased risk of COB. We found that the birth weight-reducing alleles of most of the pleiotropic loci we found (e.g. *FTO, FAIM2, TMEM18*) have previously been associated with decreased risk of COB, suggesting that concurrent genetic mechanisms may play an important role in the well-recognized positive correlation between low birth weight and low risk of COB. We also found that in 3 out of our 19 pleiotropic loci (i.e., *EBF1, SEC16B, NCOA1*) the birth weight-decreasing alleles were associated with increased risk of COB. Genetic loci near *EBF1*and *SEC16B* genes have been associated with birth weight, SGA and adiposity traits ^53–56,75,76^; however, the association of *NCOA1* with birth weight or COB traits remains elusive. Interestingly, co-activators like *NCOA1* are fundamental in uterine growth by regulating placental morphogenesis, embryo survival and interacting with estrogen receptors in the human placenta to enhance estrogen signaling which has downstream consequences on birth weight^77,78^. *NCOA1* is also highly expressed in the hypothalamus region of the brain (Fig. S6) which is implicated in appetite control, weight loss and shaping the metabolic landscape of an individual^79^ and any disruption of *NCOA1′s* function can consequently lead to several metabolic disorders which may explain its association with COB^79,80^. Therefore, the mixed direction of effects found in our study highlights the complex relationship of in-utero growth and COB traits and may provide new scopes to understand the mechanisms of development of COB.

Functional enrichment tests and pathway analysis revealed three key biological processes that may jointly contribute to the association of early life growth and development of COB. Our study found that SNPs associated with both birth weight and COB traits mapped to genes that were significantly enriched in immune system pathways and regulated the signaling of immune cells and estrogen. Estrogen plays an essential role in regulating immune response, through its interactions with the receptors on the immune cells and their functioning^81^. Previous studies^82,83^ have demonstrated that the cell‑mediated immune response is impaired in obese children along with an over‑representation of the immune and inflammatory response in adipose tissues of children. We also found the pleiotropic SNPs to be significantly enriched in Retinoic Acid (RA) signaling pathway and have *cis*-regulatory effects on gene expression in adipose tissue and muscle skeletal. Multiple studies have documented that RA regulates adipogenesis^84–86^. RA acts as a high affinity ligand for the nuclear receptor peroxisome proliferation-activated receptor β/δ which is a master regulator of lipid metabolism and glucose homeostasis. Deactivation of these receptors decreases lipid catabolism in adipose tissue and skeletal muscle, increasing the risk of obesity. Lastly, we found the pleiotropic SNPs to be significantly enriched in intracerebral signaling pathway by having regulatory effects on several cerebral tissues. Brain-derived neurotrophic factor (BDNF), a key neurotrophin with multipotent impact on brain signaling^87^ plays an important role in regulating energy homeostasis and metabolism^88–90^. BDNF acts on hypothalamic PVN and VMH neurons to suppress appetite and mediate the anorexigenic effects of MSH acting on the MCH-4 receptor^89^. Deficit in BDNF levels or signaling is attributed to the development of obesity^91,92^, indicating that pathways of intracerebral signaling may be related to COB. Our results suggest that these same causal biologic pathways likely influence early life growth, but further research is needed to understand how these pathways that influence COB also influence birth weight.

We recognize that our study has limitations. First, despite the large sample sizes of the consortia-based meta-analysis GWAS studies included in our study, there were differences in sample sizes and number of SNPs among the various GWAS studies. These contrasts may contribute to power differences in identifying pleiotropic loci. Additionally, some of the observed associations might be due to independent associations of the locus on birth weight and COB traits, due to the correlation of the traits in a causal pathway or some unmeasured characteristics. Second, the inference on joint associations of the variants in our study is based on estimates from the GPA model which performs optimally under low to moderate genetic correlation between the traits; however, under complex and weaker correlations the estimates of the GPA model can be biased. Third, some cohorts contributed to both childhood BMI and birthweight GWAS. Since GPA does not account for sample overlaps, we do not know whether the estimates are biased. Fourth, our analysis did not find functional enrichment of the pleiotropic loci between birth weight and COB under the TFBS annotation set, which requires further investigation in larger samples. An important strength of our study is the integrated modelling of functional annotations and GWAS summary statistics data from pairs of traits. This multi-trait approach has been most conducive in testing for functional enrichment and identifying novel genetic loci with their shared impacts on multiple traits, expanding our understanding of the genetic links between fetal growth and COB traits.

In conclusion, this study found that pleiotropic genetic influences and enrichment of functional annotations are substantially pervasive in the genetic architectures of birth weight and COB traits. The novel loci found in the analysis and the pathways through which the associated genes act have the potential to unravel the genetic basis that underlines associations between early growth and development of COB.

## Materials and methods

### Birth weight, childhood BMI and childhood obesity GWAS summary statistic data

Summary statistic data including *P* values and direction of effects in GWAS meta-analysis of birth weight^36^, CBMI^39^ and COB^37^ was obtained from the publicly available data of EGG (Table S2). The GWAS meta-analysis of CBMI was performed on 20 combined studies with a total sample size of 35,668 children. Children aged 2–10 years with European ancestry were included in the study^39^. The GWAS meta-analysis of COB combined 14 studies of European children aged 2–10 years with a total sample size of 5530 cases and 8318 controls. Children who had a BMI > 95th percentile were considered to be obese cases while children of BMI < 50th percentile were considered to be controls^37^. The GWAS meta-analysis of birth weight included neonates of European ancestry (BW_EU_, n = 298,142) and transethnic (African, South Asian, European) meta-analysis (BW_TR,_ n = 321,223). Individuals who were part of multiple births, who reported their birth weight in multiple visits with the mean difference in their birth weight being > 1 kg and individuals whose birth weight was < 2.5 kg or > 4.5 kg were excluded^36^. The results from the GWAS of own birth weight (as opposed to offspring birth weight), and without adjustment for maternal genotype, were used in this analysis.

### Functional annotation data

We used 4 annotation databases CADD^42^, eQTL^43^, TFBS^44^ and DHSs^45^ to functionally annotate the SNPs. Under CADD framework, implemented in CADD v1.2 (http://www.cadd.gs.washington.edu)^42^, a deleteriousness score (combined SVM score; c-score) is generated using the integrated functional and evolutionary importance of each variant from 63 annotation sources. Phred-like scores (ranged 1–99) are further generated based on the rank of each variant relative to 8.6 billion substitutions in human reference genome (-10*log_10_[rank/total]). Each variant in our analysis receiving a score ≥ 15 was assigned an annotation of 1 (deleterious) while scores < 15 were assigned an annotation of 0 (non-deleterious)^42^. In addition to CADD, which comprises a composite score, we used annotations from a variety of tissues and cell lines to elucidate the regulatory mechanisms of the risk variants. The eQTL annotations was obtained from dbGaP accession number phs000424.vN.pN on 02/11/20 (https://gtexportal.org/home/datasets)^43^ which consisted of cis-eQTL files on 49 different tissues. We took the intersection of these eQTL with the common variants of BW and COB/CBMI and the variants that overlapped were annotated as 1 and others as 0. The TFBS annotation (ftp://ccg.epfl.ch/snp2tfbs/mapped_files/annotated/)^44^ had data on 195 different transcription factors. Similar to eQTL, we took the intersection of the TFBS to annotate the common variants of birth weight and COB/CBMI. The DHSs annotation data, which has also been used in other studies^45,93^, was downloaded from (https://github.com/joepickrell/1000-genomes)^45^ and comprised of 402 binary annotations that included maps of DNase-I hypersensitive sites from different primary tissues, cell lines and cell types^94,95^.

### Statistical Analysis

We used a unified statistical approach that integrates summary statistics with functional annotations for paired traits using probabilistic models implemented in genetic analysis incorporating pleiotropy and annotation (GPA)^41^. For convenience, we briefly introduce the GPA model and its notations below.

Suppose the *P* values from two GWAS have been collected in an M × 2 matrix, p = [p_jk_], where p_jk_ denotes the *P* value of the jth SNP in the kth GWAS, k = 1,2 (in our case) and M is the number of SNPs. In the GPA model, these *P* values are assumed to come from a mixture of null (un-associated) and non-null (associated), with probability π_0_ and π_1_ = 1 − π_0_, respectively. GPA uses the Uniform distribution on [0,1] and the Beta distribution with parameters (α,1) to model the *P* values from the null and non-null groups, respectively.

Let Zj ∈ {00,10,01,11} indicate the association between the jth SNP and the two traits: Zj = 00 means the jth SNP is associated with neither of them, Zj = 10 means it is only associated with the first trait, Zj = 01 means it is only associated with the second trait, and Zj = 11 means it is associated with both trait. Thus, the four-group model is represented as:

$$\begin{array}{*{20}l} {\uppi _{00} = {\text{ Pr }}\left( {{\text{Zj }} = 00} \right):{\text{ p}}_{{{\text{j1}}}} \sim {\text{U}}\left[ {0,{1}} \right],{\text{ p}}_{{{\text{j2}}}} \sim {\text{U}}\left[ {0,{1}} \right],} \hfill & {{\text{if Zj }} = 10} \hfill \\ {\uppi _{{{1}0}} = {\text{ Pr }}\left( {{\text{Zj }} = {1}0} \right):{\text{ p}}_{{{\text{j1}}}} \sim {\text{Beta}}\left( {\upalpha {1},{1}} \right),{\text{ p}}_{{{\text{j2}}}} \sim {\text{U}}\left[ {0,{1}} \right],} \hfill & {{\text{if Zj }} = 00} \hfill \\ {\uppi _{{0{1}}} = {\text{ Pr }}\left( {{\text{Zj }} = 0{1}} \right):{\text{ p}}_{{{\text{j1}}}} \sim {\text{U}}\left[ {0,{1}} \right],{\text{ p}}_{{{\text{j2}}}} \sim {\text{Beta}}\left( {\upalpha {2},{1}} \right),} \hfill & {{\text{if Zj }} = 01} \hfill \\ {\uppi _{{{11}}} = {\text{ Pr }}\left( {{\text{Zj }} = {11}} \right):{\text{ p}}_{{{\text{j1}}}} \sim {\text{Beta}}\left( {\upalpha {1},{1}} \right),{\text{ p}}_{{{\text{j2}}}} \sim {\text{Beta}}\left( {\upalpha {2},{1}} \right),} \hfill & {{\text{if Zj }} = 11} \hfill \\ \end{array}$$

where p_j1_ and p_j2_ is the *P* value of the jth SNP in GWAS 1 and 2. GPA further incorporates functional annotation as follows. Let an M-dimensional vector A collect functional information from an annotation source, where Aj ∈ {0,1} indicates whether the jth SNP is a functional unit according to the annotation source. For example, given an eQTL data, if the jth SNP is an eQTL, then Aj = 1, otherwise Aj = 0. The relationship between Zj and Aj is described as:

$$\begin{array}{*{20}l} {{\text{q}}_{00} = {\text{ Pr }}\left( {{\text{Aj }} = {1}|{\text{ Zj }} = 00} \right),} \hfill \\ {{\text{q}}_{{{1}0}} = {\text{ Pr }}\left( {{\text{Aj }} = {1}|{\text{ Zj }} = {1}0} \right),} \hfill \\ {{\text{q}}_{{0{1}}} = {\text{ Pr }}\left( {{\text{Aj }} = {1}|{\text{ Zj }} = 0{1}} \right),} \hfill \\ {{\text{q}}_{{{11}}} = {\text{ Pr }}\left( {{\text{Aj }} = {1}|{\text{ Zj }} = {11}} \right),} \hfill \\ \end{array}$$

where q_00_ is the probability of a null SNP being annotated, q_10_ is the probability of the first trait-associated SNP being annotated, q_01_ is the probability of the second trait-associated SNP being annotated, and q_11_ is the probability of jointly associated SNP being annotated. GPA then implements an efficient EM-algorithm to obtain the estimates of the model parameters: {π_00_, π_10_, π_01_, π_11_, q_00_, q_10_, q_01_, q_11_, α}.

To assess the significance of enrichment for pleiotropy between two traits it uses the likelihood ratio test (LRT) with H_0_: π_11_ = (π_10_ + π_11_)( π_01_ + π_11_), versus H_1_: not H0. Similarly, to assess the significance of enrichment for annotation it uses LRT with H_0_: q_00_ = q_11_, versus H1: q_00_ ≠ q_11_. GPA also calculates the standard errors for the model parameters along with their covariance matrix based on an empirically observed information matrix.

After estimating the parameters, GPA assigns each SNP four posterior probabilities (PP) (estimated values of {π_00_, π_10_, π_01_, π_11_}) and controls for false discovery based on the local false discovery rate (FDR). The local FDR is defined as the probability that the jth SNP is either not associated with any trait (Fdr_0_) or is associated with the first trait (Fdr_1_), second trait (Fdr_2_) or both trait (Fdr_1,2_) given its *P* value and annotation information.

$$\begin{array}{*{20}l} {{\text{Fdr}}_{0} \left( {{\text{p}}_{{{\text{j1}}}} ,{\text{ p}}_{{{\text{j2}}}} ,{\text{A}}} \right) = {\text{Pr}}\left( {{\text{Zj}}_{00} = {1}|{\text{ p}}_{{{\text{j1}}}} ,{\text{ p}}_{{{\text{j2}}}} ,{\text{A}}} \right)} \hfill \\ {{\text{Fdr}}_{{1}} \left( {{\text{p}}_{{{\text{j1}}}} ,{\text{ p}}_{{{\text{j2}}}} ,{\text{A}}} \right) = {\text{Pr}}\left( {{\text{Zj}}_{00} + {\text{ Zj}}_{{0{1}}} = {1}|{\text{ p}}_{{{\text{j1}}}} ,{\text{ p}}_{{{\text{j2}}}} ,{\text{A}}} \right)} \hfill \\ {{\text{Fdr}}_{{2}} \left( {{\text{p}}_{{{\text{j1}}}} ,{\text{ p}}_{{{\text{j2}}}} ,{\text{A}}} \right) = {\text{Pr}}\left( {{\text{Zj}}_{00} + {\text{ Zj}}_{{{1}0}} = {1}|{\text{ p}}_{{{\text{j1}}}} ,{\text{ p}}_{{{\text{j2}}}} ,{\text{A}}} \right)} \hfill \\ {{\text{Fdr}}_{{{1},{2}}} \left( {{\text{p}}_{{{\text{j1}}}} ,{\text{ p}}_{{{\text{j2}}}} ,{\text{A}}} \right) = {\text{Pr}}\left( {{\text{Zj}}_{00} + {\text{ Zj}}_{{0{1}}} + {\text{ Zj}}_{{{1}0}} = {1}|{\text{ p}}_{{{\text{j1}}}} ,{\text{ p}}_{{{\text{j2}}}} ,{\text{A}}} \right)} \hfill \\ \end{array}$$

Finally, to infer associations at the variant level and to control the FDR at 5% we then select those SNPs with any of the four PP > 95% and FDR < 0.05. From the selected set, SNPs that achieve PP > 95% under the PP categories of {π_00_, π_10_, π_01_, π_11_} are concluded to be associated with neither, first, second or both traits. In our study, we conducted all the tests after controlling for FDR at 0.05 level and used 10,000 EM iterations.

### Pathway analysis

Pathway analysis elucidates the underlying biological processes in which genes are related by common functionality. To detect such relationships of the identified pleiotropic genetic loci in our study, we used QIAGEN’s IPA tool (https://digitalinsights.qiagen.com/). IPA is a web-based software application that allows analysis of data obtained from several sequencing platforms. IPA enables for targeted search of information on genes, proteins, chemicals, diseases, and drugs. IPA’s data analysis provides clarity in understanding the significance of data or targets of interest in relation to larger biological systems. Statistical significance of overrepresented canonical pathways was determined using Fisher’s exact test after adjustment for multiple testing using the Benjamini–Hochberg method. Statistical significance was based on *P* < 0.05 in pathways with at least two molecules.

### Colocalization analysis

To evaluated whether birthweight and childhood obesity traits share common genetic causal variant(s) in a region, we performed colocalization analysis for each of the 19 pleiotropic loci using the European ancestry GWAS summary statistics. For each genetic locus, the lead SNP with the highest posterior probability of joint association with birthweight and childhood obesity traits as well as SNPs within 500 kb window on either side of the lead SNP defined a colocalization region. Analysis was performed using the coloc R package^47^.

### Ethical approval

The NIH Office of Human Subjects Research Program granted the study an exemption from IRB review (OHSRP ID Number: 18-NICHD-00412) per 45 CFR 46 and NIH policy for the use of specimens/data.

## Supplementary Information

Supplementary Information.

## Data Availability

The data analyzed in this study are available online. Table S1 lists the URL of the data sources.
