# Supplementary material for: Pleiotropic genetic influence on birth weight and childhood obesity
Source: Sci Rep. 2021 Jan 8;11:48. doi: 10.1038/s41598-020-80084-9 (PMC7794220; doi:10.1038/s41598-020-80084-9)
Supplement: Supplementary file 1 — Supplementary Information. [file 41598_2020_80084_MOESM1_ESM.docx]

**Supplementary Information**

**Pleiotropic genetic influence on birth weight and childhood obesity**

Suvo Chatterjee^1^, Marion Ouidir^1^, Fasil Tekola-Ayele^1^

^1^Epidemiology Branch, Division of Intramural Population Health Research, *Eunice Kennedy Shriver* National Institute of Child Health and Human Development, National Institutes of Health, Bethesda, MD, USA

*Correspondence:*

Fasil Tekola-Ayele, PhD

Epidemiology Branch, Division of Intramural Population Health Research, *Eunice Kennedy Shriver* National Institute of Child Health and Human Development, National Institutes of Health

6710B Rockledge Drive, Room 3204, Bethesda, MD 20892-7004

E-mail : [ayeleft@mail.nih.gov](mailto:ayeleft@mail.nih.gov)

Tel: 301-827-6518



**Table S1: Source and sample size of the publicly available summary statistics data included in the current analysis.**

| **Traits** | **Source** | **PMID** | **Sample Size** | **Number of SNPs** | **CADD annotated SNPs** | **eQTL annotated SNPs** | **TFBS annotated SNPs** | **DHSs annotated SNPs** |
| --- | --- | --- | --- | --- | --- | --- | --- | --- |
| BW_EU_ and CBMI | Early Growth Genetics Consortium(EGG); http://egg-consortium.org | BW_EU_ : 31043758  CBMI : 26604143 | BW_EU_ = 298142  CBMI = 35668 | 2478965 | 54742 | 173301 | 498782 | 1260460 |
| BW_EU_ and COB | Early Growth Genetics Consortium(EGG); http://egg-consortium.org | BW_EU_ : 31043758  COB : 22484627 | BW_EU_ = 298142  COB = 13848 | 2482680 | 54804 | 173448 | 499623 | 1262217 |
| BW_TR_ and CBMI | Early Growth Genetics Consortium(EGG); http://egg-consortium.org | BW_TR_ : 31043758  CBMI : 26604143 | BW_TR_ : 321223  CBMI : 35668 | 2425936 | 53178 | 169127 | 489077 | 1232922 |
| BW_TR_ and COB | Early Growth Genetics Consortium(EGG); http://egg-consortium.org | BW_TR_ : 31043758  COB : 22484627 | BW_TR_ : 321223  COB : 13848 | 2427032 | 53191 | 169155 | 489334 | 1233423 |

Abbreviations: BW_EU_ and CBMI: European birth weight and childhood body mass index , BW_EU_ and COB: European birth weight and childhood obesity, BW_TR_ and CBMI : Trans-ethnic birth weight and childhood body mass index and BW_TR_ and COB : Trans-ethnic birth weight and childhood obesity. Also provided are the number of SNPs that were annotated to combined annotation dependent depletion (CADD), expression quantitative loci (eQTL), transcription factor binding sites (TFBS) and DNase I hypersensitivity sites (DHSs).

**Table S2: Functional annotation enrichment of expression quantitative loci (eQTL) tissues, DHS cell types and transcription factors in the joint analysis birthweight and Childhood body mass index.**

| **European birthweight (BW) & Childhood body mass index (CBMI)** | | | | | | | |
| --- | --- | --- | --- | --- | --- | --- | --- |
| **Type** | **Annotation** | **Enrichment fold for both traits** | **Enrichment fold for BW** | **Enrichment fold for CBMI** | **Enrichment P value (Joint)** | **Enrichment P value (BW)** | **Enrichment P value (CBMI)** |
| eQTL | Muscle Skeletal | 4.441705904 | 2.51839277 | 2.060251465 | 8.00E-44 | 1.27E-41 | 1.79E-05 |
| eQTL | Artery Aorta | 4.331093332 | 2.21496124 | 2.117798613 | 2.44E-35 | 4.38E-32 | 6.45E-06 |
| eQTL | Brain Cerebellum | 3.805270786 | 2.26511124 | 1.829608388 | 7.80E-36 | 8.91E-35 | 0.000244535 |
| eQTL | Esophagus Gastroesophageal Junction | 3.75927885 | 2.02176862 | 2.117811774 | 4.34E-26 | 1.40E-23 | 8.37E-06 |
| eQTL | Heart Atrial Appendage | 3.50391778 | 2.28276429 | 1.877049896 | 4.21E-33 | 2.38E-32 | 0.000148206 |
| eQTL | Thyroid | 3.450898725 | 2.37633854 | 1.655413954 | 5.16E-44 | 5.35E-44 | 0.002516604 |
| eQTL | Adipose Visceral Omentum | 3.343738558 | 2.21101831 | 1.518326992 | 3.86E-32 | 4.62E-32 | 0.02099558 |
| eQTL | Brain Caudate basal ganglia | 3.240813196 | 2.05437057 | 1.4791811 | 9.46E-24 | 1.54E-23 | 0.037251296 |
| eQTL | Adrenal Gland | 3.224392659 | 1.95404225 | 1.647407287 | 1.36E-20 | 2.21E-20 | 0.008631977 |
| eQTL | Brain Hippocampus | 3.026594988 | 1.74785557 | 1.649937015 | 7.06E-14 | 3.41E-13 | 0.005436268 |
| DHS | CD3+ cells | 2.806186778 | 2.28721334 | 1.377822997 | 1.90E-231 | 1.12E-230 | 9.63E-06 |
| DHS | CD56+ cells | 2.167298568 | 2.13130228 | 1.358526283 | 0.00E+00 | 0.00E+00 | 4.42E-08 |
| DHS | Embryonic stem cells | 2.077147099 | 1.59682197 | 1.25597718 | 7.10E-102 | 1.54E-100 | 8.68E-05 |
| DHS | iPS cells | 1.93136646 | 1.54869565 | 1.187039383 | 2.88E-106 | 4.47E-106 | 0.001389207 |
| DHS | Fetal brain | 1.482593402 | 1.17916586 | 1.07460927 | 4.06E-15 | 4.27E-15 | 0.192832905 |
| TFBS | ARID3A | 1.181362492 | 0.9188355 | 1.179352062 | 0.013745433 | 0.01691484 | 0.053801116 |
| TFBS | NR2C2 | 1 | 1.241192 | 1.658043196 | 0.015402996 | 0.04462806 | 0.034541542 |
| **Transethnic birthweight (BW) & Childhood body mass index (CBMI)** | | | | | | | |
| eQTL | Muscle Skeletal | 4.525394568 | 2.53940669 | 2.069045806 | 4.12E-45 | 5.01E-43 | 1.58E-05 |
| eQTL | Artery Aorta | 4.463121813 | 2.2377407 | 2.116693041 | 3.04E-36 | 5.30E-33 | 6.61E-06 |
| eQTL | Esophagus Gastroesophageal Junction | 3.987470651 | 2.08414804 | 2.138707146 | 9.68E-29 | 5.35E-26 | 6.03E-06 |
| eQTL | Brain Cerebellum | 3.810091952 | 2.3127489 | 1.831222971 | 8.33E-38 | 4.96E-37 | 0.000241027 |
| eQTL | Artery Tibial | 3.542864482 | 2.32745641 | 1.731594591 | 4.40E-37 | 5.82E-37 | 0.001359231 |
| eQTL | Adipose Visceral Omentum | 3.395300965 | 2.22957501 | 1.516841812 | 4.18E-33 | 4.24E-33 | 0.021392652 |
| eQTL | Adrenal Gland | 3.386953711 | 2.02009987 | 1.650988115 | 2.92E-23 | 6.84E-23 | 0.008373127 |
| eQTL | Heart Atrial Appendage | 3.34586269 | 2.29751811 | 1.876493614 | 9.77E-34 | 3.61E-33 | 0.000150633 |
| eQTL | Brain Caudate basal ganglia | 3.308607042 | 2.07729693 | 1.477921669 | 2.18E-24 | 3.95E-24 | 0.037791572 |
| eQTL | Brain Hippocampus | 3.12793298 | 1.77518803 | 1.664329965 | 1.64E-14 | 6.23E-14 | 0.0046178 |
| eQTL | Colon Sigmoid | 2.817963031 | 2.01123095 | 1.821387756 | 1.31E-22 | 1.35E-22 | 0.000794892 |
| DHS | CD3+ cells | 2.905322324 | 2.28574148 | 1.377332117 | 3.28E-234 | 5.55E-233 | 9.81E-06 |
| DHS | Embryonic stem cells | 2.091229919 | 1.60280535 | 1.253767324 | 4.67E-104 | 6.47E-103 | 9.89E-05 |
| DHS | Fetal brain | 1.31927558 | 1.15219433 | 1.156733442 | 9.80E-18 | 1.44E-17 | 0.000718691 |
| TFBS | IRF1 | 1.933904158 | 1.09163321 | 1.241776011 | 0.031816583 | 0.16189262 | 0.159068732 |
| TFBS | ARID3A | 1.276230337 | 0.92545121 | 1.178280078 | 0.012093466 | 0.02841776 | 0.055209239 |
| TFBS | NR2C2 | 1 | 1.26545994 | 1.658246066 | 0.01279644 | 0.02760383 | 0.034564844 |

**Table S3: Functional annotation enrichment of expression quantitative loci (eQTL) tissues, DHS cell types and transcription factors in the joint analysis birthweight and childhood obesity.**

| **European birthweight (BW) & Childhood obesity (COB)** | | | | | | | |
| --- | --- | --- | --- | --- | --- | --- | --- |
| **Type** | **Annotation** | **Enrichment fold for both traits** | **Enrichment fold for BW** | **Enrichment fold for COB** | **Enrichment P value (Joint)** | **Enrichment P value (BW)** | **Enrichment P value (COB)** |
| eQTL | Esophagus Mucosa | 5.013785478 | 2.383948821 | 4.861200003 | 3.42E-40 | 7.00E-39 | 0.000637691 |
| eQTL | Artery Aorta | 4.482556107 | 2.206851705 | 4.437047018 | 4.25E-32 | 1.54E-31 | 0.002941958 |
| eQTL | Adipose Visceral Omentum | 4.438685498 | 2.225982764 | 2.898558982 | 1.30E-32 | 1.71E-32 | 0.039580632 |
| eQTL | Small Intestine Terminal Ileum | 4.062260361 | 1.884334026 | 3.702264135 | 1.63E-17 | 2.97E-17 | 0.017075593 |
| eQTL | Whole Blood | 1.608733505 | 2.555319389 | 4.655393566 | 7.84E-40 | 8.03E-39 | 0.000589567 |
| **Transethnic birthweight (BW) & Childhood obesity (COB)** | | | | | | | |
| eQTL | Artery Aorta | 4.828230406 | 2.229845892 | 4.435047477 | 4.24E-33 | 2.01E-32 | 0.002958134 |
| eQTL | Adipose Visceral Omentum | 4.658585042 | 2.246010002 | 2.90447012 | 1.15E-33 | 1.36E-33 | 0.039214122 |
| eQTL | Esophagus Mucosa | 4.522221287 | 2.379896632 | 4.860896091 | 5.35E-40 | 5.45E-39 | 0.00063939 |
| eQTL | Small Intestine Terminal Ileum | 3.956187624 | 1.893356116 | 3.700219731 | 9.57E-18 | 1.22E-17 | 0.017146112 |
| eQTL | Whole Blood | 2.309171208 | 2.590382662 | 4.654703469 | 5.50E-41 | 2.42E-40 | 0.00059123 |
| DHS | HeLa | 1.113011459 | 1.889189062 | 1.702144302 | 4.70E-238 | 1.03E-237 | 0.001136133 |

**Table S4: Genetic loci significantly associated with both birth weight (BW) and childhood body mass index (CBMI) at posterior probability of association > 0.95.**

| **European birthweight (BW) & Childhood body mass index (CBMI)** | | | | | | | | | | | |
| --- | --- | --- | --- | --- | --- | --- | --- | --- | --- | --- | --- |
| **Chr** | **Position (hg19)** | **SNP** | **Joint Posterior Probability** | **Effect Allele** | **Beta (BW)** | **Beta (CBMI)** | **P value (BW)** | **P value (CBMI)** | **Nearest gene** | **Distance to nearest gene** | **Genetic loci** |
| 1 | 75014362 | rs10789396 | 0.97400619 | T | 0.007703 | 0.053 | 0.004172 | 6.68E-09 | *FPGT-TNNI3K* | 4245 | 1 |
| 2 | 24708396 | rs2584920 | 0.970799756 | T | -0.0102 | 0.0569 | 0.000844 | 4.31E-09 | *NCOA1* | 98948 | 2 |
| 2 | 24719710 | rs17734264 | 0.967336245 | C | -0.010025 | 0.0571 | 0.001009 | 3.66E-09 | *NCOA1* | 87634 |  |
| 2 | 24722438 | rs17734306 | 0.967240745 | A | -0.010016 | 0.0573 | 0.001016 | 2.83E-09 | *NCOA1* | 84906 |  |
| 2 | 24730847 | rs10495749 | 0.98156096 | G | -0.010624 | 0.0544 | 0.000416 | 1.30E-09 | *NCOA1* | 76497 |  |
| 2 | 24735089 | rs17790970 | 0.95518563 | G | -0.00987 | 0.0627 | 0.001688 | 1.53E-11 | *NCOA1* | 72255 |  |
| 2 | 24737833 | rs2044148 | 0.955777443 | G | -0.009889 | 0.0629 | 0.001653 | 1.23E-11 | *NCOA1* | 69511 |  |
| 2 | 24826619 | rs6720514 | 0.970848655 | T | -0.010122 | 0.0558 | 0.000836 | 7.24E-09 | *NCOA1* | 0 |  |
| 2 | 24886781 | rs2119115 | 0.964973851 | A | -0.009846 | 0.0561 | 0.001124 | 4.70E-09 | *NCOA1* | 0 |  |
| 2 | 24893059 | rs7572475 | 0.973763382 | A | -0.010319 | 0.054 | 0.000667 | 5.27E-08 | *NCOA1* | 0 |  |
| 2 | 24915342 | rs6749833 | 0.966680441 | A | -0.00953 | 0.05 | 0.000979 | 6.18E-08 | *NCOA1* | 0 |  |
| 2 | 24955160 | rs3731629 | 0.95395303 | A | -0.008898 | 0.0477 | 0.001692 | 3.09E-08 | *NCOA1* | 0 |  |
| 2 | 24959261 | rs6545698 | 0.951424474 | T | -0.008664 | 0.0511 | 0.002547 | 5.15E-08 | *NCOA1* | 0 |  |
| 2 | 24990082 | rs11892043 | 0.950704637 | A | -0.008734 | 0.0534 | 0.002362 | 6.27E-09 | *NCOA1* | 0 |  |
| 2 | 25070645 | rs1344840 | 0.968921137 | G | -0.008037 | 0.0601 | 0.005647 | 6.41E-12 | *ADCY3* | 0 | 3 |
| 12 | 49500509 | rs10747561 | 0.950050446 | C | -0.008039 | -0.037 | 0.003309 | 2.24E-05 | *LMBR1L* | 0 | 4 |
| 12 | 49505184 | rs10747562 | 0.951882721 | C | -0.007793 | -0.0378 | 0.004202 | 1.39E-05 | *LMBR1L* | 503 |  |
| 12 | 49509262 | rs7958241 | 0.951840297 | A | -0.007932 | -0.0374 | 0.003662 | 1.72E-05 | *LMBR1L* | 4581 |  |
| 12 | 49509441 | rs7958572 | 0.959040926 | C | -0.00782 | -0.0429 | 0.003993 | 7.35E-06 | *LMBR1L* | 4760 |  |
| 12 | 66379504 | rs2358954 | 0.951613435 | T | 0.026951 | 0.0365 | 4.33E-22 | 6.25E-05 | *HMGA2* | 19432 | 5 |
| **Transethnic birthweight (BW) & Childhood body mass index (CBMI)** | | | | | | | | | | | |
| 1 | 75014362 | rs10789396 | 0.952274632 | T | 0.006663 | 0.053 | 0.010331 | 6.68E-09 | *FPGT-TNNI3K* | 4245 | 1 |
| 2 | 24708396 | rs2584920 | 0.987361044 | T | -0.011 | 0.0569 | 0.000214 | 4.31E-09 | *NCOA1* | 98948 | 2 |
| 2 | 24719710 | rs17734264 | 0.986323676 | C | -0.010864 | 0.0571 | 0.000243 | 3.66E-09 | *NCOA1* | 87634 |  |
| 2 | 24722438 | rs17734306 | 0.984557787 | A | -0.010733 | 0.0573 | 0.000295 | 2.83E-09 | *NCOA1* | 84906 |  |
| 2 | 24727019 | rs2165740 | 0.957786628 | G | -0.009916 | 0.0628 | 0.001427 | 3.87E-10 | *NCOA1* | 80325 |  |
| 2 | 24730581 | rs17734407 | 0.956029798 | A | -0.009568 | 0.0627 | 0.00211 | 4.25E-10 | *NCOA1* | 76763 |  |
| 2 | 24730847 | rs10495749 | 0.990624421 | G | -0.011174 | 0.0544 | 0.000138 | 1.30E-09 | *NCOA1* | 76497 |  |
| 2 | 24735089 | rs17790970 | 0.975063293 | G | -0.010516 | 0.0627 | 0.000633 | 1.53E-11 | *NCOA1* | 72255 |  |
| 2 | 24737412 | rs11125632 | 0.955803202 | C | -0.009854 | 0.0648 | 0.001518 | 4.00E-09 | *NCOA1* | 69932 |  |
| 2 | 24737833 | rs2044148 | 0.974153397 | G | -0.010475 | 0.0629 | 0.000669 | 1.23E-11 | *NCOA1* | 69511 |  |
| 2 | 24812163 | rs17734650 | 0.957087092 | A | -0.009572 | 0.0621 | 0.002029 | 5.94E-10 | *NCOA1* | 0 |  |
| 2 | 24815624 | rs749046 | 0.964314302 | A | -0.010175 | 0.0622 | 0.001096 | 5.70E-10 | *NCOA1* | 0 |  |
| 2 | 24826619 | rs6720514 | 0.985037911 | T | -0.010727 | 0.0558 | 0.000275 | 7.24E-09 | *NCOA1* | 0 |  |
| 2 | 24871798 | rs11125733 | 0.956233656 | A | -0.009901 | 0.0655 | 0.001513 | 1.41E-10 | *NCOA1* | 0 |  |
| 2 | 24875403 | rs11125735 | 0.95341642 | G | -0.009811 | 0.0655 | 0.001669 | 1.36E-10 | *NCOA1* | 0 |  |
| 2 | 24875778 | rs719189 | 0.955712862 | A | -0.00988 | 0.0652 | 0.001541 | 1.67E-10 | *NCOA1* | 0 |  |
| 2 | 24881271 | rs17791703 | 0.95467193 | T | -0.009449 | 0.0632 | 0.002216 | 2.39E-10 | *NCOA1* | 0 |  |
| 2 | 24886781 | rs2119115 | 0.978943992 | A | -0.010274 | 0.0561 | 0.000474 | 4.70E-09 | *NCOA1* | 0 |  |
| 2 | 24893059 | rs7572475 | 0.985185974 | A | -0.010822 | 0.054 | 0.000244 | 5.27E-08 | *NCOA1* | 0 |  |
| 2 | 24895302 | rs11676900 | 0.959919916 | C | -0.009979 | 0.0619 | 0.001307 | 2.06E-09 | *NCOA1* | 0 |  |
| 2 | 24902072 | rs11693687 | 0.956150835 | A | -0.009515 | 0.0631 | 0.002103 | 2.67E-10 | *NCOA1* | 0 |  |
| 2 | 24906195 | rs11674421 | 0.952308841 | G | -0.009682 | 0.0636 | 0.001731 | 2.03E-10 | *NCOA1* | 0 |  |
| 2 | 24915342 | rs6749833 | 0.97768702 | A | -0.009773 | 0.05 | 0.000476 | 6.18E-08 | *NCOA1* | 0 |  |
| 2 | 24918283 | rs11894404 | 0.962871002 | G | -0.010088 | 0.0624 | 0.001161 | 1.57E-09 | *NCOA1* | 0 |  |
| 2 | 24924438 | rs17792389 | 0.956007288 | A | -0.00961 | 0.0641 | 0.001903 | 1.43E-10 | *NCOA1* | 0 |  |
| 2 | 24955160 | rs3731629 | 0.961924348 | A | -0.008892 | 0.0477 | 0.001162 | 3.09E-08 | *NCOA1* | 0 |  |
| 2 | 24959261 | rs6545698 | 0.957570756 | T | -0.008612 | 0.0511 | 0.001888 | 5.15E-08 | *NCOA1* | 0 |  |
| 2 | 24972217 | rs6761875 | 0.953767425 | T | -0.008463 | 0.0531 | 0.002253 | 5.95E-09 | *NCOA1* | 0 |  |
| 2 | 24990082 | rs11892043 | 0.956602771 | A | -0.008826 | 0.0534 | 0.001469 | 6.27E-09 | *NCOA1* | 0 |  |
| 2 | 24991569 | rs17737058 | 0.961097398 | C | -0.007999 | 0.0647 | 0.007608 | 4.85E-11 | *NCOA1* | 0 |  |
| 2 | 25070645 | rs1344840 | 0.975285014 | G | -0.008224 | 0.0601 | 0.003773 | 6.41E-12 | *ADCY3* | 0 | 3 |
| 5 | 158446223 | rs6887211 | 0.950597967 | C | 0.010699 | -0.0366 | 9.62E-05 | 8.44E-05 | *EBF1* | 0 | 4 |
| 12 | 49500509 | rs10747561 | 0.953459432 | C | -0.007953 | -0.037 | 0.002577 | 2.24E-05 | *LMBR1L* | 0 | 5 |
| 12 | 49505184 | rs10747562 | 0.957284169 | C | -0.00781 | -0.0378 | 0.002959 | 1.39E-05 | *LMBR1L* | 503 |  |
| 12 | 49509441 | rs7958572 | 0.965958544 | C | -0.007943 | -0.0429 | 0.002459 | 7.35E-06 | *LMBR1L* | 4760 |  |
| 12 | 66379504 | rs2358954 | 0.951075253 | T | 0.026498 | 0.0365 | 5.46E-23 | 6.25E-05 | *HMGA2* | 19432 | 6 |

**Table S5: Genetic loci significantly associated with both birth weight (BW) and childhood obesity (COB) at posterior probability of association > 0.95**

| **European birthweight (BW) & Childhood obesity (COB)** | | | | | | | | | | | |
| --- | --- | --- | --- | --- | --- | --- | --- | --- | --- | --- | --- |
| **Chr** | **Position (hg19)** | **SNP** | **Joint Posterior Probability** | **Effect Allele** | **Beta (BW)** | **Beta (COB)** | **P value (BW)** | **P value (COB)** | **Nearest gene** | **Distance to nearest gene** | **Genetic loci** |
| 1 | 74977277 | rs6690871 | 0.997407904 | A | -0.00677 | -0.1493 | 0.009442 | 3.89E-08 | *FPGT-TNNI3K* | 0 | 1 |
| 1 | 74977425 | rs953567 | 0.976787093 | A | -0.00653 | -0.1339 | 0.012666 | 2.86E-06 | *FPGT-TNNI3K* | 0 |  |
| 1 | 74977870 | rs1040070 | 0.997033229 | G | 0.006294 | 0.1492 | 0.014694 | 2.78E-08 | *FPGT-TNNI3K* | 0 |  |
| 1 | 74979975 | rs12036473 | 0.97301915 | A | -0.00497 | -0.133 | 0.05853 | 1.29E-06 | *FPGT-TNNI3K* | 0 |  |
| 1 | 74983835 | rs10493544 | 0.997908801 | T | 0.006962 | 0.1469 | 0.006852 | 3.15E-08 | *FPGT-TNNI3K* | 0 |  |
| 1 | 74991402 | rs1514177 | 0.991714514 | C | 0.006157 | 0.1348 | 0.016617 | 3.83E-07 | *FPGT-TNNI3K* | 0 |  |
| 1 | 74991596 | rs1514176 | 0.994395843 | G | 0.00714 | 0.1349 | 0.005471 | 3.75E-07 | *FPGT-TNNI3K* | 0 |  |
| 1 | 74991644 | rs1514175 | 0.991800667 | A | 0.007041 | 0.136 | 0.006124 | 3.02E-07 | *FPGT-TNNI3K* | 0 |  |
| 1 | 74992278 | rs6604867 | 0.997880948 | T | 0.007264 | 0.1444 | 0.004627 | 5.36E-08 | *FPGT-TNNI3K* | 0 |  |
| 1 | 74992546 | rs6604866 | 0.997742079 | G | 0.00704 | 0.145 | 0.006067 | 4.72E-08 | *FPGT-TNNI3K* | 0 |  |
| 1 | 74993063 | rs1514174 | 0.997702368 | C | 0.006992 | 0.1451 | 0.006428 | 4.62E-08 | *FPGT-TNNI3K* | 0 |  |
| 1 | 74993318 | rs7526762 | 0.997795987 | A | 0.007104 | 0.1449 | 0.005627 | 4.82E-08 | *FPGT-TNNI3K* | 0 |  |
| 1 | 74995110 | rs1514173 | 0.992022531 | C | -0.00626 | -0.1381 | 0.015726 | 3.71E-07 | *FPGT-TNNI3K* | 0 |  |
| 1 | 74995225 | rs7551507 | 0.997502654 | C | 0.007228 | 0.1432 | 0.004831 | 7.75E-08 | *FPGT-TNNI3K* | 0 |  |
| 1 | 74997762 | rs12042908 | 0.997590427 | A | 0.007255 | 0.1435 | 0.004704 | 7.28E-08 | *FPGT-TNNI3K* | 0 |  |
| 1 | 74997795 | rs6703637 | 0.990376812 | T | -0.00622 | -0.1374 | 0.01647 | 5.18E-07 | *FPGT-TNNI3K* | 0 |  |
| 1 | 74997956 | rs6698622 | 0.990801701 | G | -0.00633 | -0.1374 | 0.01463 | 5.18E-07 | *FPGT-TNNI3K* | 0 |  |
| 1 | 74999713 | rs2344508 | 0.997104367 | G | 0.007242 | 0.1426 | 0.004759 | 1.10E-07 | *FPGT-TNNI3K* | 0 |  |
| 1 | 75000011 | rs12142020 | 0.985126181 | A | -0.00629 | -0.1367 | 0.038996 | 5.91E-07 | *FPGT-TNNI3K* | 0 |  |
| 1 | 75001683 | rs3894212 | 0.990329548 | T | -0.00625 | -0.1372 | 0.015847 | 5.38E-07 | *FPGT-TNNI3K* | 0 |  |
| 1 | 75002193 | rs12566985 | 0.996752044 | G | 0.007077 | 0.1421 | 0.005805 | 1.22E-07 | *FPGT-TNNI3K* | 0 |  |
| 1 | 75002667 | rs3845345 | 0.989266527 | G | -0.00624 | -0.1362 | 0.016022 | 6.50E-07 | *FPGT-TNNI3K* | 0 |  |
| 1 | 75003500 | rs12041852 | 0.996934863 | G | 0.007172 | 0.1423 | 0.005179 | 1.17E-07 | *FPGT-TNNI3K* | 0 |  |
| 1 | 75003710 | rs12041912 | 0.997022349 | G | 0.007153 | 0.1427 | 0.005299 | 1.08E-07 | *FPGT-TNNI3K* | 0 |  |
| 1 | 75004048 | rs3845347 | 0.997015729 | G | 0.007244 | 0.1423 | 0.004786 | 1.17E-07 | *FPGT-TNNI3K* | 0 |  |
| 1 | 75004611 | rs6604872 | 0.996963996 | T | 0.007172 | 0.1424 | 0.005177 | 1.15E-07 | *FPGT-TNNI3K* | 0 |  |
| 1 | 75004875 | rs10890130 | 0.988667136 | G | -0.00621 | -0.1358 | 0.016624 | 7.01E-07 | *FPGT-TNNI3K* | 0 |  |
| 1 | 75004943 | rs7520945 | 0.996883128 | T | 0.007176 | 0.1421 | 0.005154 | 1.22E-07 | *FPGT-TNNI3K* | 0 |  |
| 1 | 75005067 | rs7553348 | 0.997134033 | G | 0.007136 | 0.1432 | 0.005402 | 9.73E-08 | *FPGT-TNNI3K* | 0 |  |
| 1 | 75005238 | rs7553158 | 0.9970154 | G | 0.007053 | 0.1426 | 0.005962 | 9.83E-08 | *FPGT-TNNI3K* | 0 |  |
| 1 | 75005363 | rs11210477 | 0.989139965 | G | -0.00617 | -0.1369 | 0.017358 | 6.28E-07 | *FPGT-TNNI3K* | 0 |  |
| 1 | 75005776 | rs6656785 | 0.974307034 | A | -0.00617 | -0.1292 | 0.017929 | 2.80E-06 | *FPGT-TNNI3K* | 0 |  |
| 1 | 75006027 | rs3895907 | 0.996599604 | A | 0.006993 | 0.142 | 0.006433 | 1.24E-07 | *FPGT-TNNI3K* | 0 |  |
| 1 | 75006720 | rs7514705 | 0.997309857 | T | 0.007113 | 0.1441 | 0.005628 | 8.09E-08 | *FPGT-TNNI3K* | 0 |  |
| 1 | 75007008 | rs3765680 | 0.990084348 | A | -0.00611 | -0.1381 | 0.018595 | 5.00E-07 | *FPGT-TNNI3K* | 0 |  |
| 1 | 75008008 | rs6604871 | 0.989035897 | T | -0.0059 | -0.138 | 0.023186 | 5.10E-07 | *FPGT-TNNI3K* | 0 |  |
| 1 | 75008411 | rs11210478 | 0.989796592 | A | -0.00605 | -0.1381 | 0.019957 | 5.00E-07 | *FPGT-TNNI3K* | 0 |  |
| 1 | 75011358 | rs6669189 | 0.989932151 | C | -0.00624 | -0.138 | 0.016584 | 5.62E-07 | *FPGT-TNNI3K* | 1241 |  |
| 1 | 75012637 | rs10218727 | 0.988699919 | G | -0.00596 | -0.1381 | 0.022661 | 5.51E-07 | *FPGT-TNNI3K* | 2520 |  |
| 1 | 75013054 | rs3843262 | 0.990570538 | T | -0.00606 | -0.1409 | 0.021862 | 3.95E-07 | *FPGT-TNNI3K* | 2937 |  |
| 1 | 75014362 | rs10789396 | 0.997232469 | T | 0.007703 | 0.1612 | 0.004172 | 1.10E-07 | *FPGT-TNNI3K* | 4245 |  |
| 1 | 177852580 | rs633715 | 0.977654408 | T | 0.00407 | -0.1699 | 0.19203 | 2.08E-07 | *SEC16B* | 45660 | 2 |
| 1 | 177873210 | rs574367 | 0.983031603 | G | 0.004404 | -0.1741 | 0.159993 | 1.24E-07 | *SEC16B* | 25030 |  |
| 1 | 177875514 | rs527248 | 0.982374864 | A | 0.004383 | -0.1735 | 0.16206 | 1.37E-07 | *SEC16B* | 22726 |  |
| 1 | 177876946 | rs589500 | 0.983374161 | C | 0.004516 | -0.1737 | 0.14932 | 1.33E-07 | *SEC16B* | 21294 |  |
| 1 | 177881651 | rs693232 | 0.983558143 | C | 0.004533 | -0.1738 | 0.147811 | 1.31E-07 | *SEC16B* | 16589 |  |
| 1 | 177889480 | rs543874 | 0.971279422 | A | 0.003614 | -0.178 | 0.252343 | 8.54E-08 | *SEC16B* | 8760 |  |
| 1 | 177894287 | rs506589 | 0.977485766 | T | 0.003229 | -0.1782 | 0.306948 | 8.27E-08 | *SEC16B* | 3953 |  |
| 1 | 177913519 | rs10913469 | 0.973174914 | T | 0.002594 | -0.1773 | 0.413486 | 7.99E-08 | *SEC16B* | 0 |  |
| 2 | 600575 | rs2683992 | 0.980391467 | G | -0.00227 | -0.224 | 0.497712 | 1.96E-10 | *TMEM18* | 67396 | 3 |
| 2 | 601905 | rs2867105 | 0.9798236 | T | -0.00214 | -0.2241 | 0.522932 | 1.03E-10 | *TMEM18* | 66066 |  |
| 2 | 610603 | rs2867131 | 0.981331476 | T | -0.00248 | -0.2341 | 0.465755 | 4.87E-11 | *TMEM18* | 57368 |  |
| 2 | 614168 | rs2947411 | 0.983961917 | A | -0.00301 | -0.2381 | 0.37012 | 1.16E-11 | *TMEM18* | 53803 |  |
| 2 | 614210 | rs2860323 | 0.981161767 | A | -0.00243 | -0.2379 | 0.474072 | 1.21E-11 | *TMEM18* | 53761 |  |
| 2 | 615140 | rs7567570 | 0.984058185 | T | -0.00313 | -0.223 | 0.352679 | 8.31E-10 | *TMEM18* | 52831 |  |
| 2 | 621461 | rs6548237 | 0.975541378 | A | -0.00126 | -0.239 | 0.709728 | 1.11E-11 | *TMEM18* | 46510 |  |
| 2 | 621558 | rs939584 | 0.977334826 | C | -0.00257 | -0.191 | 0.444212 | 2.49E-08 | *TMEM18* | 46413 |  |
| 2 | 622161 | rs1320331 | 0.981610728 | G | -0.0025 | -0.2431 | 0.457339 | 4.28E-12 | *TMEM18* | 45810 |  |
| 2 | 622225 | rs1320330 | 0.981595305 | T | -0.0025 | -0.2383 | 0.457325 | 1.28E-11 | *TMEM18* | 45746 |  |
| 2 | 622531 | rs939583 | 0.981081282 | C | -0.00239 | -0.2422 | 0.477704 | 5.12E-12 | *TMEM18* | 45440 |  |
| 2 | 622723 | rs939582 | 0.980974226 | A | -0.00243 | -0.2279 | 0.471553 | 3.52E-10 | *TMEM18* | 45248 |  |
| 2 | 622827 | rs2867125 | 0.981014877 | T | -0.00238 | -0.24 | 0.480064 | 7.95E-12 | *TMEM18* | 45144 |  |
| 2 | 623691 | rs11127483 | 0.981152452 | G | -0.00247 | -0.2256 | 0.463407 | 4.21E-10 | *TMEM18* | 44280 |  |
| 2 | 623798 | rs11127484 | 0.981420902 | T | -0.00247 | -0.2409 | 0.464435 | 6.65E-12 | *TMEM18* | 44173 |  |
| 2 | 623935 | rs6719518 | 0.98094661 | C | -0.00237 | -0.2419 | 0.482858 | 6.27E-12 | *TMEM18* | 44036 |  |
| 2 | 623976 | rs6728726 | 0.979570089 | T | -0.00208 | -0.2461 | 0.536653 | 2.10E-11 | *TMEM18* | 43995 |  |
| 2 | 624034 | rs6711012 | 0.97968107 | G | -0.0021 | -0.242 | 0.533207 | 6.15E-12 | *TMEM18* | 43937 |  |
| 2 | 624524 | rs2867123 | 0.976326577 | G | -0.00141 | -0.2418 | 0.675221 | 6.40E-12 | *TMEM18* | 43447 |  |
| 2 | 624581 | rs2867122 | 0.981203349 | A | -0.00242 | -0.2418 | 0.472859 | 6.40E-12 | *TMEM18* | 43390 |  |
| 2 | 624678 | rs2903492 | 0.981256382 | G | -0.00243 | -0.242 | 0.470825 | 6.15E-12 | *TMEM18* | 43293 |  |
| 2 | 625029 | rs7576624 | 0.981159722 | C | -0.00241 | -0.242 | 0.474571 | 6.15E-12 | *TMEM18* | 42942 |  |
| 2 | 625057 | rs7576635 | 0.981263282 | C | -0.00243 | -0.242 | 0.470558 | 6.15E-12 | *TMEM18* | 42914 |  |
| 2 | 628504 | rs6744646 | 0.977518199 | A | -0.00166 | -0.243 | 0.623421 | 5.80E-12 | *TMEM18* | 39467 |  |
| 2 | 628524 | rs6744653 | 0.977535575 | A | -0.00166 | -0.243 | 0.622676 | 5.80E-12 | *TMEM18* | 39447 |  |
| 2 | 629244 | rs12463617 | 0.974624601 | A | -0.00156 | -0.2278 | 0.644556 | 6.83E-09 | *TMEM18* | 38727 |  |
| 2 | 629510 | rs6743060 | 0.978474199 | C | -0.00186 | -0.244 | 0.583031 | 4.75E-12 | *TMEM18* | 38461 |  |
| 2 | 629694 | rs6752470 | 0.973960512 | T | -0.00093 | -0.244 | 0.78289 | 4.75E-12 | *TMEM18* | 38277 |  |
| 2 | 629881 | rs12995480 | 0.977632231 | T | -0.00168 | -0.2436 | 0.618608 | 5.15E-12 | *TMEM18* | 38090 |  |
| 2 | 629914 | rs6732471 | 0.978338825 | G | -0.00183 | -0.2436 | 0.588664 | 5.15E-12 | *TMEM18* | 38057 |  |
| 2 | 630024 | rs13007080 | 0.978306665 | A | -0.00182 | -0.2436 | 0.590015 | 5.15E-12 | *TMEM18* | 37947 |  |
| 2 | 630034 | rs13007086 | 0.978997983 | A | -0.00197 | -0.244 | 0.561265 | 4.75E-12 | *TMEM18* | 37937 |  |
| 2 | 630323 | rs6725549 | 0.977885552 | C | -0.00179 | -0.2297 | 0.596692 | 2.87E-10 | *TMEM18* | 37648 |  |
| 2 | 630339 | rs6731348 | 0.977618742 | A | -0.00168 | -0.2439 | 0.619218 | 4.85E-12 | *TMEM18* | 37632 |  |
| 2 | 630662 | rs6731688 | 0.977802215 | A | -0.0018 | -0.2266 | 0.595023 | 4.96E-10 | *TMEM18* | 37309 |  |
| 2 | 630995 | rs5017303 | 0.977937444 | T | -0.00175 | -0.2408 | 0.605236 | 8.97E-12 | *TMEM18* | 36976 |  |
| 2 | 631099 | rs5017300 | 0.97750965 | C | -0.00166 | -0.2408 | 0.623477 | 8.97E-12 | *TMEM18* | 36872 |  |
| 2 | 631528 | rs7585056 | 0.97827033 | A | -0.00182 | -0.2404 | 0.591117 | 9.70E-12 | *TMEM18* | 36443 |  |
| 2 | 632028 | rs11127485 | 0.979830275 | C | -0.00214 | -0.2404 | 0.526887 | 9.70E-12 | *TMEM18* | 35943 |  |
| 2 | 632146 | rs12623218 | 0.977737748 | T | -0.00171 | -0.2418 | 0.613876 | 7.36E-12 | *TMEM18* | 35825 |  |
| 2 | 632300 | rs12992154 | 0.977277169 | G | -0.00169 | -0.2264 | 0.616553 | 5.14E-10 | *TMEM18* | 35671 |  |
| 2 | 632348 | rs13021737 | 0.977591319 | A | -0.00176 | -0.2263 | 0.603196 | 5.23E-10 | *TMEM18* | 35623 |  |
| 2 | 632550 | rs13012571 | 0.978401831 | C | -0.00184 | -0.24 | 0.585646 | 9.14E-12 | *TMEM18* | 35421 |  |
| 2 | 634905 | rs6548238 | 0.977343856 | T | -0.00163 | -0.2452 | 0.629178 | 2.82E-11 | *TMEM18* | 33066 |  |
| 2 | 635200 | rs6734363 | 0.977588758 | G | -0.00168 | -0.2397 | 0.620023 | 9.70E-12 | *TMEM18* | 32771 |  |
| 2 | 635721 | rs6755502 | 0.977748395 | T | -0.00171 | -0.2386 | 0.613138 | 1.05E-11 | *TMEM18* | 32250 |  |
| 2 | 637597 | rs13388043 | 0.978849889 | C | -0.00194 | -0.2399 | 0.567072 | 8.11E-12 | *TMEM18* | 30374 |  |
| 2 | 637830 | rs13393304 | 0.977348865 | A | -0.00162 | -0.2401 | 0.630495 | 7.80E-12 | *TMEM18* | 30141 |  |
| 2 | 638144 | rs4854344 | 0.976423461 | G | -0.00143 | -0.2445 | 0.671334 | 3.22E-12 | *TMEM18* | 29827 |  |
| 2 | 642499 | rs7601028 | 0.97665986 | C | -0.0015 | -0.2517 | 0.656923 | 6.16E-11 | *TMEM18* | 25472 |  |
| 2 | 643303 | rs7604609 | 0.975780769 | C | -0.00151 | -0.2565 | 0.65436 | 1.90E-09 | *TMEM18* | 24668 |  |
| 2 | 644953 | rs7561317 | 0.975749119 | A | -0.0013 | -0.2412 | 0.700916 | 6.26E-12 | *TMEM18* | 23018 |  |
| 2 | 646145 | rs11127491 | 0.97666157 | T | -0.00148 | -0.24 | 0.660208 | 9.14E-12 | *TMEM18* | 21826 |  |
| 2 | 646364 | rs10189761 | 0.972350686 | T | -0.0006 | -0.2363 | 0.857934 | 1.65E-11 | *TMEM18* | 21607 |  |
| 2 | 646674 | rs10190052 | 0.976431206 | T | -0.00144 | -0.2413 | 0.670534 | 7.07E-12 | *TMEM18* | 21297 |  |
| 2 | 646767 | rs10173167 | 0.976261528 | G | -0.0014 | -0.2413 | 0.678023 | 7.07E-12 | *TMEM18* | 21204 |  |
| 2 | 646803 | rs7571957 | 0.976477527 | T | -0.00145 | -0.2412 | 0.66848 | 7.21E-12 | *TMEM18* | 21168 |  |
| 2 | 647580 | rs10193244 | 0.97625801 | T | -0.0014 | -0.2416 | 0.678223 | 6.66E-12 | *TMEM18* | 20391 |  |
| 2 | 647760 | rs4854348 | 0.976158687 | A | -0.00138 | -0.2415 | 0.682607 | 6.79E-12 | *TMEM18* | 20211 |  |
| 2 | 647861 | rs4854349 | 0.976333869 | T | -0.00142 | -0.2415 | 0.674856 | 6.79E-12 | *TMEM18* | 20110 |  |
| 2 | 648198 | rs7570198 | 0.9786449 | C | -0.00187 | -0.2415 | 0.575703 | 6.79E-12 | *TMEM18* | 19773 |  |
| 2 | 648758 | rs4423631 | 0.977967121 | T | -0.00174 | -0.2414 | 0.604167 | 6.93E-12 | *TMEM18* | 19213 |  |
| 2 | 648810 | rs4452188 | 0.976228055 | A | 0.001394 | 0.2414 | 0.679519 | 6.93E-12 | *TMEM18* | 19161 |  |
| 2 | 649347 | rs1320338 | 0.97605169 | T | 0.001358 | 0.2413 | 0.687327 | 7.07E-12 | *TMEM18* | 18624 |  |
| 2 | 649867 | rs1320337 | 0.975858361 | A | 0.001319 | 0.2416 | 0.695986 | 6.66E-12 | *TMEM18* | 18104 |  |
| 2 | 650012 | rs1320336 | 0.975195297 | G | 0.001255 | 0.2288 | 0.710103 | 3.77E-10 | *TMEM18* | 17959 |  |
| 2 | 650143 | rs2867108 | 0.976069713 | T | 0.001362 | 0.2426 | 0.686613 | 6.28E-12 | *TMEM18* | 17828 |  |
| 2 | 650479 | rs13386517 | 0.976300205 | G | 0.001409 | 0.2426 | 0.676399 | 6.28E-12 | *TMEM18* | 17492 |  |
| 2 | 650519 | rs13401686 | 0.975257317 | A | 0.001196 | 0.2434 | 0.723077 | 6.16E-12 | *TMEM18* | 17452 |  |
| 2 | 650560 | rs13386627 | 0.975945186 | G | 0.001336 | 0.2434 | 0.692168 | 6.16E-12 | *TMEM18* | 17411 |  |
| 2 | 650647 | rs4613321 | 0.976044312 | A | 0.001357 | 0.2432 | 0.687727 | 6.41E-12 | *TMEM18* | 17324 |  |
| 2 | 650828 | rs13386964 | 0.976093466 | G | 0.001367 | 0.2424 | 0.685423 | 7.51E-12 | *TMEM18* | 17143 |  |
| 2 | 651030 | rs2867109 | 0.976145977 | T | 0.001378 | 0.2427 | 0.683139 | 7.08E-12 | *TMEM18* | 16941 |  |
| 2 | 651105 | rs2867110 | 0.976245973 | G | 0.001398 | 0.2425 | 0.678679 | 7.37E-12 | *TMEM18* | 16866 |  |
| 2 | 651349 | rs2867112 | 0.97822566 | T | 0.001846 | 0.2424 | 0.59319 | 7.51E-12 | *TMEM18* | 16622 |  |
| 2 | 651365 | rs2867113 | 0.986352551 | G | 0.004095 | 0.2424 | 0.289092 | 7.51E-12 | *TMEM18* | 16606 |  |
| 2 | 651407 | rs12714414 | 0.979960164 | T | 0.002307 | 0.2428 | 0.521881 | 6.94E-12 | *TMEM18* | 16564 |  |
| 2 | 651430 | rs12714415 | 0.976932811 | T | 0.001586 | 0.243 | 0.648616 | 6.67E-12 | *TMEM18* | 16541 |  |
| 2 | 651507 | rs6719980 | 0.976074352 | T | 0.001366 | 0.243 | 0.686363 | 6.67E-12 | *TMEM18* | 16464 |  |
| 2 | 652247 | rs7608050 | 0.976453648 | G | 0.001445 | 0.2429 | 0.669467 | 7.82E-12 | *TMEM18* | 15724 |  |
| 2 | 652542 | rs7574359 | 0.975317959 | T | 0.001215 | 0.2428 | 0.720126 | 7.98E-12 | *TMEM18* | 15429 |  |
| 2 | 653093 | rs13415094 | 0.975939406 | T | 0.00134 | 0.2434 | 0.69221 | 8.14E-12 | *TMEM18* | 14878 |  |
| 2 | 653195 | rs13396935 | 0.977124661 | G | 0.001583 | 0.2436 | 0.640172 | 7.83E-12 | *TMEM18* | 14776 |  |
| 2 | 653354 | rs13397165 | 0.97556596 | G | 0.001265 | 0.2437 | 0.708976 | 7.68E-12 | *TMEM18* | 14617 |  |
| 2 | 653874 | rs10188334 | 0.976715257 | C | 0.001583 | 0.2304 | 0.641969 | 4.47E-10 | *TMEM18* | 14097 |  |
| 2 | 24942448 | rs11889662 | 0.950032127 | T | -0.00777 | 0.1419 | 0.011406 | 1.06E-05 | *NCOA1* | 0 | 4 |
| 2 | 24946188 | rs11693308 | 0.978702965 | C | -0.00804 | 0.1559 | 0.008983 | 2.93E-06 | *NCOA1* | 0 |  |
| 2 | 24991569 | rs17737058 | 0.952050281 | C | -0.00751 | 0.1364 | 0.014678 | 1.30E-05 | *NCOA1* | 0 |  |
| 2 | 25075281 | rs7608976 | 0.970449095 | G | -0.00039 | -0.1609 | 0.88235 | 2.40E-09 | *ADCY3* | 0 | 5 |
| 2 | 25075675 | rs7597332 | 0.963829851 | A | -0.00024 | -0.1559 | 0.927462 | 2.55E-08 | *ADCY3* | 0 |  |
| 2 | 25076126 | rs13387729 | 0.969536515 | A | -0.0002 | -0.1605 | 0.939578 | 1.72E-09 | *ADCY3* | 0 |  |
| 2 | 25079770 | rs11675457 | 0.977119721 | C | -0.00154 | -0.1537 | 0.553621 | 6.19E-09 | *ADCY3* | 0 |  |
| 2 | 25082273 | rs916485 | 0.97731107 | T | -0.00135 | -0.1605 | 0.600414 | 1.29E-09 | *ADCY3* | 0 |  |
| 2 | 25082414 | rs1541984 | 0.976861676 | G | -0.00157 | -0.153 | 0.546743 | 8.27E-09 | *ADCY3* | 0 |  |
| 2 | 25082926 | rs11687089 | 0.980247722 | T | -0.00202 | -0.1553 | 0.438356 | 6.46E-09 | *ADCY3* | 0 |  |
| 2 | 25083310 | rs7576788 | 0.967578687 | T | -0.00138 | -0.1449 | 0.593907 | 6.85E-08 | *ADCY3* | 0 |  |
| 2 | 25086827 | rs1529897 | 0.975739438 | T | -0.00125 | -0.1557 | 0.629631 | 3.93E-09 | *ADCY3* | 0 |  |
| 2 | 25096692 | rs11892869 | 0.978825836 | A | -0.00163 | -0.1626 | 0.527899 | 2.16E-09 | *ADCY3* | 0 |  |
| 2 | 25096952 | rs7567997 | 0.97932156 | T | -0.00162 | -0.163 | 0.530932 | 6.12E-10 | *ADCY3* | 0 |  |
| 2 | 25097072 | rs7580081 | 0.978727175 | G | -0.00153 | -0.1629 | 0.554437 | 6.27E-10 | *ADCY3* | 0 |  |
| 2 | 25097644 | rs13407913 | 0.979072936 | A | -0.0016 | -0.1623 | 0.53669 | 8.42E-10 | *ADCY3* | 0 |  |
| 2 | 25097939 | rs13410999 | 0.979249293 | T | -0.00161 | -0.1624 | 0.532055 | 7.07E-10 | *ADCY3* | 0 |  |
| 2 | 25099357 | rs6545776 | 0.979626897 | A | -0.00168 | -0.1621 | 0.516245 | 7.59E-10 | *ADCY3* | 0 |  |
| 2 | 25100328 | rs2384058 | 0.978769659 | A | -0.00153 | -0.1633 | 0.55386 | 5.69E-10 | *ADCY3* | 0 |  |
| 2 | 25100338 | rs2384059 | 0.978668697 | C | -0.00153 | -0.1622 | 0.554626 | 7.41E-10 | *ADCY3* | 0 |  |
| 2 | 25100738 | rs10865315 | 0.976950211 | C | -0.00148 | -0.1545 | 0.569162 | 5.16E-09 | *ADCY3* | 0 |  |
| 2 | 25100902 | rs2033656 | 0.978492295 | C | -0.0015 | -0.1618 | 0.560385 | 8.16E-10 | *ADCY3* | 0 |  |
| 2 | 25101092 | rs2033655 | 0.977672109 | G | -0.00163 | -0.1535 | 0.530607 | 6.47E-09 | *ADCY3* | 0 |  |
| 2 | 25103108 | rs2033654 | 0.979067371 | C | -0.00161 | -0.161 | 0.532046 | 1.15E-09 | *ADCY3* | 0 |  |
| 2 | 25103446 | rs2033653 | 0.979231033 | T | -0.00161 | -0.1622 | 0.532161 | 7.41E-10 | *ADCY3* | 0 |  |
| 2 | 25103967 | rs7591460 | 0.978728074 | A | -0.00173 | -0.1548 | 0.505091 | 4.82E-09 | *ADCY3* | 0 |  |
| 2 | 25107759 | rs11686663 | 0.977907678 | C | -0.00162 | -0.1544 | 0.53171 | 5.28E-09 | *ADCY3* | 0 |  |
| 2 | 25108197 | rs1865689 | 0.978252109 | T | -0.00167 | -0.1545 | 0.51985 | 5.16E-09 | *ADCY3* | 0 |  |
| 2 | 25109302 | rs6545790 | 0.979566451 | A | -0.00165 | -0.1636 | 0.522806 | 5.29E-10 | *ADCY3* | 0 |  |
| 2 | 25110962 | rs6749170 | 0.980623615 | A | -0.00182 | -0.1637 | 0.481831 | 5.17E-10 | *ADCY3* | 0 |  |
| 2 | 25118885 | rs6545800 | 0.97810526 | C | -0.00143 | -0.1629 | 0.57973 | 6.27E-10 | *ADCY3* | 0 |  |
| 2 | 25120086 | rs6752483 | 0.97887378 | C | -0.00156 | -0.1625 | 0.5453 | 8.03E-10 | *ADCY3* | 0 |  |
| 2 | 25120713 | rs6723803 | 0.978344079 | G | -0.00148 | -0.1625 | 0.566615 | 8.03E-10 | *ADCY3* | 0 |  |
| 2 | 25120851 | rs6713978 | 0.978174893 | T | -0.00145 | -0.1625 | 0.573486 | 8.03E-10 | *ADCY3* | 0 |  |
| 2 | 25121125 | rs6756609 | 0.978781576 | C | -0.00155 | -0.1624 | 0.548647 | 8.22E-10 | *ADCY3* | 0 |  |
| 2 | 25122840 | rs4077678 | 0.978183453 | C | -0.00146 | -0.1619 | 0.570931 | 9.26E-10 | *ADCY3* | 0 |  |
| 2 | 25123463 | rs3903070 | 0.97752758 | C | -0.00135 | -0.1628 | 0.601134 | 7.47E-10 | *ADCY3* | 0 |  |
| 2 | 25126230 | rs6712981 | 0.977586197 | A | -0.00136 | -0.1623 | 0.599171 | 7.24E-10 | *ADCY3* | 0 |  |
| 2 | 25126328 | rs6726199 | 0.976918278 | G | -0.00126 | -0.1622 | 0.624293 | 8.62E-10 | *ADCY3* | 0 |  |
| 2 | 25126715 | rs6545809 | 0.976733312 | C | -0.00123 | -0.1622 | 0.634514 | 7.41E-10 | *ADCY3* | 0 |  |
| 2 | 25128351 | rs6706316 | 0.977551822 | C | -0.00136 | -0.1621 | 0.597539 | 8.83E-10 | *ADCY3* | 0 |  |
| 2 | 25128719 | rs6721750 | 0.977529153 | A | -0.00136 | -0.162 | 0.598083 | 9.04E-10 | *ADCY3* | 0 |  |
| 2 | 25128730 | rs6724772 | 0.977595704 | T | -0.00136 | -0.162 | 0.597719 | 7.78E-10 | *ADCY3* | 0 |  |
| 2 | 25130451 | rs11689546 | 0.979190733 | G | -0.00322 | 0.1652 | 0.523807 | 1.38E-09 | *ADCY3* | 0 |  |
| 2 | 25130462 | rs10200566 | 0.975419402 | T | -0.00102 | -0.1632 | 0.692405 | 6.79E-10 | *ADCY3* | 0 |  |
| 2 | 25130542 | rs10198275 | 0.977407322 | A | -0.00132 | -0.164 | 0.610036 | 5.61E-10 | *ADCY3* | 0 |  |
| 2 | 25130907 | rs6733224 | 0.977780507 | T | -0.00136 | -0.1659 | 0.597834 | 4.14E-10 | *ADCY3* | 0 |  |
| 2 | 25131316 | rs6545814 | 0.978594441 | A | -0.0015 | -0.1642 | 0.561666 | 5.34E-10 | *ADCY3* | 0 |  |
| 2 | 25131986 | rs11900505 | 0.9773713 | A | -0.00133 | -0.1614 | 0.60473 | 8.97E-10 | *ADCY3* | 0 |  |
| 2 | 25132192 | rs6722587 | 0.978391605 | C | -0.0015 | -0.1615 | 0.560952 | 1.02E-09 | *ADCY3* | 0 |  |
| 2 | 25135620 | rs2384061 | 0.974577 | G | -0.00129 | -0.1516 | 0.620226 | 9.91E-09 | *ADCY3* | 0 |  |
| 2 | 25136866 | rs10203386 | 0.977599866 | T | -0.00137 | -0.162 | 0.595168 | 9.04E-10 | *ADCY3* | 0 |  |
| 2 | 25138040 | rs6737082 | 0.966611126 | A | 0.000248 | -0.1523 | 0.923296 | 1.25E-08 | *ADCY3* | 0 |  |
| 2 | 25141538 | rs11676272 | 0.976914652 | A | -0.00146 | -0.1612 | 0.565022 | 5.77E-09 | *ADCY3* | 0 |  |
| 2 | 25150116 | rs6752378 | 0.980646516 | C | -0.00176 | -0.1695 | 0.49033 | 1.05E-10 | *ADCY3* | 8060 |  |
| 2 | 25150296 | rs10182181 | 0.980720132 | A | -0.00177 | -0.1682 | 0.48624 | 1.46E-10 | *ADCY3* | 8240 |  |
| 2 | 25153986 | rs6726261 | 0.98510267 | T | 0.002796 | -0.1597 | 0.290792 | 4.73E-09 | *ADCY3* | 11930 |  |
| 2 | 25156773 | rs2384054 | 0.979039207 | T | -0.00154 | -0.1649 | 0.548192 | 3.30E-10 | *DNAJC27* | 9730 |  |
| 2 | 25158008 | rs713586 | 0.978292128 | T | -0.00143 | -0.1647 | 0.578287 | 3.46E-10 | *DNAJC27* | 8495 |  |
| 2 | 25158281 | rs713587 | 0.977688318 | C | -0.00133 | -0.1646 | 0.603149 | 3.55E-10 | *DNAJC27* | 8222 |  |
| 2 | 25169200 | rs1172294 | 0.985971156 | A | -0.00266 | -0.1698 | 0.296878 | 2.21E-10 | *DNAJC27* | 0 |  |
| 2 | 25176200 | rs754536 | 0.979007946 | C | 0.001612 | 0.164 | 0.531166 | 1.36E-09 | *DNAJC27* | 0 |  |
| 2 | 25176277 | rs754537 | 0.979171823 | A | 0.001634 | 0.1642 | 0.525621 | 1.30E-09 | *DNAJC27* | 0 |  |
| 2 | 25187599 | rs4665736 | 0.987308112 | C | 0.002932 | 0.1694 | 0.254284 | 2.43E-10 | *DNAJC27* | 0 |  |
| 2 | 25205427 | rs1982200 | 0.985738931 | C | 0.002658 | 0.1614 | 0.296418 | 8.97E-10 | *LOC729723* | 0 |  |
| 2 | 25239264 | rs11125884 | 0.987718315 | A | 0.003028 | 0.1648 | 0.239352 | 4.62E-10 | *LOC729723* | 0 |  |
| 2 | 25239969 | rs12466350 | 0.986644934 | C | 0.00293 | 0.1608 | 0.254851 | 2.83E-09 | *LOC729723* | 0 |  |
| 2 | 25286435 | rs1077492 | 0.988706763 | C | 0.003235 | 0.1648 | 0.209773 | 5.39E-10 | *EFR3B* | 0 | 6 |
| 2 | 25301755 | rs478222 | 0.9824503 | A | 0.00334 | 0.1455 | 0.201576 | 8.52E-08 | *EFR3B* | 0 |  |
| 2 | 25303772 | rs493090 | 0.979457178 | G | 0.003393 | 0.1408 | 0.194399 | 1.59E-07 | *EFR3B* | 0 |  |
| 2 | 25304574 | rs567359 | 0.970688164 | A | 0.00343 | 0.1358 | 0.189418 | 4.28E-07 | *EFR3B* | 0 |  |
| 2 | 25305136 | rs483428 | 0.977937355 | C | 0.003364 | 0.1399 | 0.198186 | 1.90E-07 | *EFR3B* | 0 |  |
| 2 | 25305504 | rs551573 | 0.966359746 | G | 0.003378 | 0.1344 | 0.19612 | 5.61E-07 | *EFR3B* | 0 |  |
| 2 | 25305756 | rs1530016 | 0.977530479 | C | 0.003387 | 0.1395 | 0.195086 | 2.06E-07 | *EFR3B* | 0 |  |
| 2 | 25316584 | rs522806 | 0.974326255 | C | 0.00332 | 0.1375 | 0.204211 | 2.76E-07 | *EFR3B* | 0 |  |
| 2 | 25333735 | rs519111 | 0.972687739 | T | 0.003056 | 0.1386 | 0.243948 | 2.46E-07 | *EFR3B* | 0 |  |
| 2 | 25334340 | rs839652 | 0.97445376 | C | 0.00297 | 0.1411 | 0.257578 | 1.85E-07 | *EFR3B* | 0 |  |
| 2 | 25404982 | rs4665774 | 0.954789398 | T | -0.00284 | 0.175 | 0.386739 | 4.51E-07 | *POMC* | 13422 | 7 |
| 4 | 45175691 | rs13130484 | 0.980206591 | C | -0.00329 | -0.1434 | 0.202376 | 1.30E-07 | *GNPDA2* | 447078 | 8 |
| 4 | 45179883 | rs12641981 | 0.979653748 | C | -0.00335 | -0.142 | 0.193795 | 1.55E-07 | *GNPDA2* | 451270 |  |
| 4 | 130731284 | rs4864201 | 0.971817315 | T | 0.005051 | 0.1355 | 0.058066 | 1.41E-06 | *C4orf33* | 697440 | 9 |
| 12 | 50169070 | rs2720296 | 0.96503215 | G | -0.00122 | -0.1583 | 0.649719 | 7.50E-08 | *TMBIM6* | 10352 | 10 |
| 12 | 50171714 | rs2720295 | 0.965981792 | A | -0.00293 | -0.1526 | 0.284462 | 3.47E-07 | *TMBIM6* | 12996 |  |
| 12 | 50175380 | rs2720293 | 0.971277396 | A | -0.00374 | -0.1559 | 0.203192 | 3.70E-07 | *NCKAP5L* | 9547 |  |
| 12 | 50177324 | rs11836282 | 0.959408891 | A | -0.0025 | -0.1517 | 0.391075 | 3.38E-07 | *NCKAP5L* | 7603 |  |
| 12 | 50183874 | rs2603107 | 0.967902829 | T | -0.00263 | -0.1386 | 0.320582 | 2.46E-07 | *NCKAP5L* | 1053 |  |
| 12 | 50183966 | rs1470909 | 0.969729683 | T | -0.0026 | -0.1396 | 0.32633 | 2.02E-07 | *NCKAP5L* | 961 |  |
| 12 | 50188434 | rs2603105 | 0.958654985 | T | -0.00277 | -0.1435 | 0.317034 | 4.80E-07 | *NCKAP5L* | 0 |  |
| 12 | 50192009 | rs1075366 | 0.967667719 | T | -0.00279 | -0.1377 | 0.290471 | 2.95E-07 | *NCKAP5L* | 0 |  |
| 12 | 50199949 | rs2603112 | 0.985785316 | A | -0.00385 | -0.1475 | 0.153378 | 7.10E-08 | *NCKAP5L* | 0 |  |
| 12 | 50204337 | rs7313563 | 0.985956854 | C | -0.00385 | -0.1525 | 0.150437 | 7.09E-08 | *NCKAP5L* | 0 |  |
| 12 | 50204709 | rs4898530 | 0.981265913 | A | -0.00327 | -0.1432 | 0.21199 | 9.73E-08 | *NCKAP5L* | 0 |  |
| 12 | 50206723 | rs10875969 | 0.976650607 | G | -0.00294 | -0.1405 | 0.261575 | 1.35E-07 | *NCKAP5L* | 0 |  |
| 12 | 50207126 | rs11169162 | 0.981723761 | T | -0.00339 | -0.146 | 0.204517 | 9.62E-08 | *NCKAP5L* | 0 |  |
| 12 | 50208343 | rs11169163 | 0.971524369 | A | -0.00244 | -0.146 | 0.365774 | 1.33E-07 | *NCKAP5L* | 0 |  |
| 12 | 50212628 | rs2336448 | 0.976711884 | C | -0.00308 | -0.1408 | 0.240039 | 1.59E-07 | *NCKAP5L* | 0 |  |
| 12 | 50214637 | rs4075681 | 0.96122369 | C | -0.00259 | -0.1435 | 0.347526 | 3.59E-07 | *NCKAP5L* | 0 |  |
| 12 | 50241013 | rs11169176 | 0.95402301 | G | -0.00422 | -0.1273 | 0.100008 | 2.14E-06 | *BCDIN3D* | 4100 |  |
| 12 | 50244767 | rs10875980 | 0.957297724 | A | -0.00416 | -0.1268 | 0.104762 | 1.79E-06 | *BCDIN3D* | 7854 |  |
| 12 | 50245706 | rs10875982 | 0.958695091 | A | -0.00407 | -0.1275 | 0.112664 | 1.57E-06 | *BCDIN3D* | 8793 |  |
| 12 | 50247468 | rs7138803 | 0.98800015 | G | -0.00317 | -0.1672 | 0.229275 | 6.50E-10 | *BCDIN3D* | 10555 |  |
| 12 | 50263148 | rs7132908 | 0.967831783 | G | -0.00432 | -0.1635 | 0.099439 | 1.24E-08 | *FAIM2* | 0 |  |
| 12 | 50285061 | rs297924 | 0.992562288 | T | -0.00452 | -0.1531 | 0.087538 | 1.20E-08 | *FAIM2* | 0 |  |
| 12 | 50285562 | rs17201502 | 0.99405914 | C | -0.00479 | -0.165 | 0.073371 | 2.51E-09 | *FAIM2* | 0 |  |
| 16 | 53798523 | rs8047395 | 0.989114178 | G | -0.00341 | -0.1634 | 0.180955 | 4.08E-09 | *FTO* | 0 | 11 |
| 16 | 53799507 | rs9937053 | 0.996690366 | G | -0.00549 | -0.1776 | 0.032649 | 2.22E-10 | *FTO* | 0 |  |
| 16 | 53799905 | rs9928094 | 0.996683891 | A | -0.00546 | -0.1942 | 0.033576 | 7.20E-13 | *FTO* | 0 |  |
| 16 | 53799977 | rs9930333 | 0.996671883 | T | -0.00548 | -0.1776 | 0.033083 | 1.64E-10 | *FTO* | 0 |  |
| 16 | 53800387 | rs12446228 | 0.964653111 | A | -0.004 | -0.1368 | 0.127617 | 1.02E-06 | *FTO* | 0 |  |
| 16 | 53800568 | rs9939973 | 0.996963535 | G | -0.0056 | -0.1939 | 0.029378 | 6.39E-13 | *FTO* | 0 |  |
| 16 | 53800629 | rs9940646 | 0.994065847 | C | -0.00448 | -0.1978 | 0.08126 | 4.06E-13 | *FTO* | 0 |  |
| 16 | 53800754 | rs9940128 | 0.996698255 | G | -0.00547 | -0.1942 | 0.033358 | 5.89E-13 | *FTO* | 0 |  |
| 16 | 53800954 | rs1421085 | 0.98700937 | T | -0.00579 | -0.191 | 0.02557 | 1.40E-12 | *FTO* | 0 |  |
| 16 | 53801549 | rs9923147 | 0.996864217 | C | -0.00555 | -0.1941 | 0.030848 | 6.06E-13 | *FTO* | 0 |  |
| 16 | 53801985 | rs9923544 | 0.996882832 | C | -0.00556 | -0.1942 | 0.030571 | 5.89E-13 | *FTO* | 0 |  |
| 16 | 53803156 | rs8055197 | 0.992176815 | G | -0.00408 | -0.1568 | 0.108484 | 4.03E-09 | *FTO* | 0 |  |
| 16 | 53803574 | rs1558902 | 0.991863139 | T | -0.00391 | -0.1905 | 0.131348 | 1.32E-12 | *FTO* | 0 |  |
| 16 | 53804340 | rs1861866 | 0.992260197 | C | -0.0041 | -0.1568 | 0.106692 | 4.03E-09 | *FTO* | 0 |  |
| 16 | 53804965 | rs10852521 | 0.991389212 | T | -0.00394 | -0.1552 | 0.1217 | 5.78E-09 | *FTO* | 0 |  |
| 16 | 53805207 | rs11075985 | 0.996691533 | C | -0.00547 | -0.1957 | 0.033465 | 3.91E-13 | *FTO* | 0 |  |
| 16 | 53806145 | rs2058908 | 0.957536654 | T | -0.0057 | -0.1745 | 0.048647 | 3.39E-06 | *FTO* | 0 |  |
| 16 | 53806280 | rs9922047 | 0.979536811 | C | -0.00331 | -0.1392 | 0.193526 | 1.58E-07 | *FTO* | 0 |  |
| 16 | 53807764 | rs17817288 | 0.991302702 | A | -0.00395 | -0.1543 | 0.120851 | 7.07E-09 | *FTO* | 0 |  |
| 16 | 53808258 | rs1477196 | 0.967025008 | A | -0.00401 | -0.1365 | 0.126411 | 8.98E-07 | *FTO* | 0 |  |
| 16 | 53809247 | rs1121980 | 0.997015671 | G | -0.00562 | -0.1961 | 0.028623 | 2.85E-13 | *FTO* | 0 |  |
| 16 | 53810686 | rs7193144 | 0.997594649 | T | -0.00602 | -0.1926 | 0.020638 | 4.96E-13 | *FTO* | 0 |  |
| 16 | 53812614 | rs8057044 | 0.994794655 | G | -0.00472 | -0.1637 | 0.064114 | 5.17E-10 | *FTO* | 0 |  |
| 16 | 53813367 | rs17817449 | 0.997400068 | T | -0.0059 | -0.1927 | 0.023222 | 3.93E-13 | *FTO* | 0 |  |
| 16 | 53813450 | rs8043757 | 0.997427265 | A | -0.00592 | -0.1928 | 0.022855 | 3.82E-13 | *FTO* | 0 |  |
| 16 | 53815161 | rs11075987 | 0.992247887 | T | -0.00398 | -0.1616 | 0.117395 | 6.31E-10 | *FTO* | 0 |  |
| 16 | 53816275 | rs8050136 | 0.997559067 | C | -0.006 | -0.1909 | 0.021102 | 5.27E-13 | *FTO* | 0 |  |
| 16 | 53816647 | rs4783819 | 0.97743397 | G | -0.00404 | -0.1397 | 0.123279 | 4.07E-07 | *FTO* | 0 |  |
| 16 | 53816752 | rs8051591 | 0.997624302 | A | -0.00604 | -0.1932 | 0.020257 | 2.77E-13 | *FTO* | 0 |  |
| 16 | 53816838 | rs9935401 | 0.997753876 | G | -0.00612 | -0.1935 | 0.018607 | 2.54E-13 | *FTO* | 0 |  |
| 16 | 53818460 | rs3751812 | 0.990243298 | G | -0.00624 | -0.1932 | 0.016508 | 2.77E-13 | *FTO* | 0 |  |
| 16 | 53818708 | rs3751813 | 0.99602729 | G | -0.00519 | -0.166 | 0.042114 | 6.39E-10 | *FTO* | 0 |  |
| 16 | 53819169 | rs9936385 | 0.997777239 | T | -0.00615 | -0.184 | 0.018093 | 5.77E-11 | *FTO* | 0 |  |
| 16 | 53819198 | rs9923233 | 0.997730761 | G | -0.00611 | -0.1838 | 0.01878 | 2.24E-11 | *FTO* | 0 |  |
| 16 | 53819877 | rs11075989 | 0.997690908 | C | -0.0061 | -0.1781 | 0.019123 | 7.70E-11 | *FTO* | 0 |  |
| 16 | 53819893 | rs11075990 | 0.997705534 | A | -0.00609 | -0.1934 | 0.019217 | 2.62E-13 | *FTO* | 0 |  |
| 16 | 53820527 | rs9939609 | 0.997669042 | T | -0.00607 | -0.1934 | 0.019682 | 2.62E-13 | *FTO* | 0 |  |
| 16 | 53821615 | rs7202116 | 0.989039838 | A | -0.00607 | -0.1892 | 0.019704 | 1.39E-11 | *FTO* | 0 |  |
| 16 | 53821862 | rs7201850 | 0.99743628 | C | -0.00588 | -0.1831 | 0.022585 | 2.66E-11 | *FTO* | 0 |  |
| 16 | 53822651 | rs7185735 | 0.997567008 | A | -0.00601 | -0.1927 | 0.021 | 3.93E-13 | *FTO* | 0 |  |
| 16 | 53825488 | rs9941349 | 0.997793857 | C | -0.0061 | -0.1978 | 0.01811 | 1.16E-13 | *FTO* | 0 |  |
| 16 | 53827179 | rs9931494 | 0.997787888 | C | -0.0061 | -0.1974 | 0.018184 | 1.30E-13 | *FTO* | 0 |  |
| 16 | 53828066 | rs17817964 | 0.997974269 | C | -0.00628 | -0.1933 | 0.01591 | 4.09E-13 | *FTO* | 0 |  |
| 16 | 53828752 | rs7190492 | 0.970738242 | A | -0.00398 | -0.1359 | 0.128617 | 6.88E-07 | *FTO* | 0 |  |
| 16 | 53830452 | rs9930501 | 0.994978579 | A | -0.00477 | -0.1881 | 0.063009 | 1.70E-12 | *FTO* | 0 |  |
| 16 | 53830465 | rs9930506 | 0.994175707 | A | -0.00451 | -0.1874 | 0.078935 | 2.05E-12 | *FTO* | 0 |  |
| 16 | 53830491 | rs9932754 | 0.995010898 | T | -0.00478 | -0.1868 | 0.062388 | 1.98E-12 | *FTO* | 0 |  |
| 16 | 53831146 | rs9922708 | 0.994296019 | C | -0.00455 | -0.1866 | 0.076468 | 2.09E-12 | *FTO* | 0 |  |
| 16 | 53831771 | rs9922619 | 0.994410985 | G | -0.00458 | -0.1866 | 0.074137 | 2.09E-12 | *FTO* | 0 |  |
| 16 | 53839135 | rs8044769 | 0.985595125 | T | -0.00291 | -0.1482 | 0.24657 | 1.25E-08 | *FTO* | 0 |  |
| 16 | 53842908 | rs12149832 | 0.994757697 | G | -0.00474 | -0.1837 | 0.067201 | 5.49E-12 | *FTO* | 0 |  |
| 16 | 53845487 | rs11642841 | 0.998026647 | C | -0.00636 | -0.1882 | 0.015183 | 2.48E-11 | *FTO* | 0 |  |
| 16 | 53848561 | rs1861867 | 0.960794538 | A | -0.00318 | -0.1742 | 0.246332 | 5.93E-07 | *FTO* | 0 |  |
| 18 | 38765542 | rs17697435 | 0.984847118 | T | -0.01047 | -0.1859 | 0.006913 | 1.87E-06 | *KC6* | 294692 | 12 |
| 18 | 38765623 | rs17697453 | 0.98425585 | A | -0.01047 | -0.1854 | 0.006905 | 2.00E-06 | *KC6* | 294611 |  |
| 18 | 38765659 | rs17697518 | 0.985572449 | C | -0.01069 | -0.1855 | 0.005827 | 1.85E-06 | *KC6* | 294575 |  |
| 18 | 57732689 | rs4940927 | 0.964823454 | A | -0.00015 | 0.1678 | 0.959149 | 1.68E-08 | *PMAIP1* | 161150 | 13 |
| 18 | 57744189 | rs7240566 | 0.957871574 | A | 0.001716 | -0.1577 | 0.560908 | 2.05E-07 | *PMAIP1* | 172650 |  |
| 18 | 57744576 | rs11520442 | 0.954907659 | T | 0.00138 | -0.1579 | 0.639825 | 1.98E-07 | *PMAIP1* | 173037 |  |
| 18 | 57751185 | rs4299252 | 0.951166385 | A | 0.001579 | -0.1558 | 0.592479 | 2.86E-07 | *PMAIP1* | 179646 |  |
| 18 | 57751960 | rs8091524 | 0.950549318 | T | 0.001527 | -0.1558 | 0.605084 | 2.86E-07 | *PMAIP1* | 180421 |  |
| 18 | 57755117 | rs6567155 | 0.952931497 | C | 0.001466 | -0.1563 | 0.619348 | 2.39E-07 | *PMAIP1* | 183578 |  |
| 18 | 57766512 | rs1539952 | 0.95301274 | A | 0.001642 | -0.1562 | 0.579182 | 2.67E-07 | *PMAIP1* | 194973 |  |
| 18 | 57775295 | rs9966951 | 0.968008096 | G | 0.003152 | -0.1424 | 0.248255 | 3.63E-07 | *PMAIP1* | 203756 |  |
| 18 | 57777991 | rs9951795 | 0.966079291 | C | 0.002375 | -0.1452 | 0.382768 | 2.13E-07 | *PMAIP1* | 206452 |  |
| 18 | 57781188 | rs948760 | 0.967459758 | A | 0.002363 | -0.1459 | 0.384687 | 1.86E-07 | *PMAIP1* | 209649 |  |
| 18 | 57787559 | rs1893512 | 0.976720071 | T | 0.002627 | -0.1499 | 0.334034 | 7.65E-08 | *PMAIP1* | 216020 |  |
| 18 | 57790052 | rs756190 | 0.979943084 | A | 0.003464 | -0.147 | 0.202854 | 1.36E-07 | *PMAIP1* | 218513 |  |
| 18 | 57790225 | rs6567157 | 0.971479428 | T | 0.002558 | -0.1465 | 0.346799 | 1.49E-07 | *PMAIP1* | 218686 |  |
| 18 | 57791081 | rs8087080 | 0.97188545 | G | 0.002584 | -0.1466 | 0.342011 | 1.47E-07 | *PMAIP1* | 219542 |  |
| 18 | 57793209 | rs1942880 | 0.972579734 | C | 0.002903 | -0.1458 | 0.286117 | 1.90E-07 | *PMAIP1* | 221670 |  |
| 18 | 57793589 | rs8084834 | 0.973609862 | C | 0.002904 | -0.1464 | 0.285816 | 1.69E-07 | *PMAIP1* | 222050 |  |
| 18 | 57797485 | rs953442 | 0.968243264 | T | 0.00224 | -0.161 | 0.450384 | 1.25E-07 | *PMAIP1* | 225946 |  |
| 18 | 57798110 | rs952044 | 0.972868559 | C | 0.002923 | -0.1464 | 0.282997 | 1.88E-07 | *PMAIP1* | 226571 |  |
| 18 | 57799449 | rs1942859 | 0.976839711 | T | 0.00332 | -0.1472 | 0.223071 | 1.78E-07 | *PMAIP1* | 227910 |  |
| 18 | 57804346 | rs8095404 | 0.955547236 | A | 0.005775 | -0.1255 | 0.057084 | 3.24E-06 | *PMAIP1* | 232807 |  |
| 18 | 57811982 | rs17700144 | 0.974089265 | G | 0.001621 | -0.1825 | 0.605279 | 1.46E-08 | *MC4R* | 226580 |  |
| 18 | 57829135 | rs6567160 | 0.97547787 | T | 0.001146 | -0.1974 | 0.703783 | 1.85E-10 | *MC4R* | 209427 |  |
| 18 | 57838401 | rs663129 | 0.977097172 | G | 0.001428 | -0.1992 | 0.635183 | 1.27E-10 | *MC4R* | 200161 |  |
| 18 | 57839769 | rs571312 | 0.977075404 | C | 0.001423 | -0.1986 | 0.636183 | 1.25E-10 | *MC4R* | 198793 |  |
| 18 | 57848369 | rs523288 | 0.983752749 | A | 0.00268 | -0.1974 | 0.372723 | 1.85E-10 | *MC4R* | 190193 |  |
| 18 | 57848531 | rs2168711 | 0.976526187 | T | 0.001335 | -0.1975 | 0.657837 | 1.82E-10 | *MC4R* | 190031 |  |
| 18 | 57849023 | rs12967135 | 0.978500381 | G | 0.001707 | -0.195 | 0.570728 | 3.06E-10 | *MC4R* | 189539 |  |
| 18 | 57850422 | rs538656 | 0.98048294 | G | 0.002068 | -0.1963 | 0.492361 | 2.68E-10 | *MC4R* | 188140 |  |
| 18 | 57851097 | rs17782313 | 0.978061937 | T | 0.001622 | -0.1962 | 0.589727 | 2.73E-10 | *MC4R* | 187465 |  |
| 18 | 57851763 | rs10871777 | 0.977368676 | A | 0.001498 | -0.1946 | 0.617163 | 3.33E-10 | *MC4R* | 186799 |  |
| 18 | 57852587 | rs476828 | 0.97837304 | T | 0.001676 | -0.1947 | 0.57546 | 3.26E-10 | *MC4R* | 185975 |  |
| 18 | 57852948 | rs11152213 | 0.977972938 | A | 0.001604 | -0.1956 | 0.592453 | 3.10E-10 | *MC4R* | 185614 |  |
| 18 | 57858802 | rs492443 | 0.972508986 | A | 0.000984 | -0.1749 | 0.733095 | 6.60E-09 | *MC4R* | 179760 |  |
| 18 | 57858829 | rs8089364 | 0.969802382 | T | 0.000828 | -0.1722 | 0.774727 | 1.41E-08 | *MC4R* | 179733 |  |
| 18 | 57859563 | rs12969709 | 0.970736 | C | 0.000948 | -0.1713 | 0.74309 | 1.34E-08 | *MC4R* | 178999 |  |
| 18 | 57861663 | rs921971 | 0.969731043 | T | 0.000828 | -0.1709 | 0.77464 | 1.44E-08 | *MC4R* | 176899 |  |
| 18 | 57861961 | rs1457489 | 0.96953179 | G | 0.00079 | -0.1704 | 0.784613 | 1.42E-08 | *MC4R* | 176601 |  |
| 18 | 57876034 | rs11665563 | 0.969233305 | C | 0.001055 | -0.1667 | 0.71467 | 2.60E-08 | *MC4R* | 162528 |  |
| 18 | 57876227 | rs11663816 | 0.957051922 | T | 0.000984 | -0.1582 | 0.733319 | 1.27E-07 | *MC4R* | 162335 |  |
| 18 | 57877800 | rs11664883 | 0.96895095 | T | 0.001026 | -0.1666 | 0.722154 | 2.65E-08 | *MC4R* | 160762 |  |
| 18 | 57884750 | rs12970134 | 0.967454981 | G | 0.000895 | -0.1665 | 0.756368 | 3.01E-08 | *MC4R* | 153812 |  |
| 18 | 57893618 | rs8083289 | 0.964917955 | C | 0.000762 | -0.1655 | 0.792289 | 4.04E-08 | *MC4R* | 144944 |  |
| 18 | 57896742 | rs17175643 | 0.96700963 | C | 0.001114 | -0.165 | 0.699944 | 4.44E-08 | *MC4R* | 141820 |  |
| 18 | 57897803 | rs12960928 | 0.967380669 | T | 0.00118 | -0.1649 | 0.683764 | 4.52E-08 | *MC4R* | 140759 |  |
| 18 | 57903604 | rs12964203 | 0.965439325 | T | 0.001079 | -0.1639 | 0.709689 | 5.45E-08 | *MC4R* | 134958 |  |
| 18 | 57904088 | rs590215 | 0.967189885 | C | 0.001262 | -0.1636 | 0.662705 | 5.19E-08 | *MC4R* | 134474 |  |
| 18 | 57907311 | rs2168708 | 0.963859194 | G | 0.00098 | -0.1644 | 0.735339 | 6.11E-08 | *MC4R* | 131251 |  |
| 18 | 57911330 | rs12966550 | 0.96449743 | A | 0.001005 | -0.1653 | 0.729215 | 5.74E-08 | *MC4R* | 127232 |  |
| **Transethnic birthweight (BW) & Childhood obesity (COB)** | | | | | | | | | | | |
| 1 | 74977277 | rs6690871 | 0.991484231 | A | -0.00688 | -0.1493 | 0.006273 | 3.89E-08 | *FPGT-TNNI3K* | 0 | 1 |
| 1 | 74977425 | rs953567 | 0.972267297 | A | -0.00675 | -0.1339 | 0.007502 | 2.86E-06 | *FPGT-TNNI3K* | 0 |  |
| 1 | 74977870 | rs1040070 | 0.98198001 | G | 0.005761 | 0.1492 | 0.021627 | 2.78E-08 | *FPGT-TNNI3K* | 0 |  |
| 1 | 74983835 | rs10493544 | 0.987256183 | T | 0.006268 | 0.1469 | 0.012359 | 3.15E-08 | *FPGT-TNNI3K* | 0 |  |
| 1 | 74991402 | rs1514177 | 0.962586247 | C | 0.004898 | 0.1348 | 0.047829 | 3.83E-07 | *FPGT-TNNI3K* | 0 |  |
| 1 | 74991596 | rs1514176 | 0.977222678 | G | 0.005751 | 0.1349 | 0.020277 | 3.75E-07 | *FPGT-TNNI3K* | 0 |  |
| 1 | 74991644 | rs1514175 | 0.984358787 | A | 0.005634 | 0.136 | 0.022973 | 3.02E-07 | *FPGT-TNNI3K* | 0 |  |
| 1 | 74992278 | rs6604867 | 0.988475657 | T | 0.006425 | 0.1444 | 0.009878 | 5.36E-08 | *FPGT-TNNI3K* | 0 |  |
| 1 | 74992546 | rs6604866 | 0.985706866 | G | 0.006084 | 0.145 | 0.014254 | 4.72E-08 | *FPGT-TNNI3K* | 0 |  |
| 1 | 74993063 | rs1514174 | 0.985203823 | C | 0.006032 | 0.1451 | 0.015107 | 4.62E-08 | *FPGT-TNNI3K* | 0 |  |
| 1 | 74993318 | rs7526762 | 0.987400346 | A | 0.006279 | 0.1449 | 0.011591 | 4.82E-08 | *FPGT-TNNI3K* | 0 |  |
| 1 | 74995110 | rs1514173 | 0.975414662 | C | -0.00566 | -0.1381 | 0.023326 | 3.71E-07 | *FPGT-TNNI3K* | 0 |  |
| 1 | 74995225 | rs7551507 | 0.988229902 | C | 0.006446 | 0.1432 | 0.009647 | 7.75E-08 | *FPGT-TNNI3K* | 0 |  |
| 1 | 74997762 | rs12042908 | 0.988384986 | A | 0.00646 | 0.1435 | 0.009539 | 7.28E-08 | *FPGT-TNNI3K* | 0 |  |
| 1 | 74997795 | rs6703637 | 0.978141971 | T | -0.00599 | -0.1374 | 0.016578 | 5.18E-07 | *FPGT-TNNI3K* | 0 |  |
| 1 | 74997956 | rs6698622 | 0.979650493 | G | -0.00611 | -0.1374 | 0.014477 | 5.18E-07 | *FPGT-TNNI3K* | 0 |  |
| 1 | 74999713 | rs2344508 | 0.987918077 | G | 0.006469 | 0.1426 | 0.0094 | 1.10E-07 | *FPGT-TNNI3K* | 0 |  |
| 1 | 75000011 | rs12142020 | 0.957891222 | A | -0.00578 | -0.1367 | 0.051272 | 5.91E-07 | *FPGT-TNNI3K* | 0 |  |
| 1 | 75001683 | rs3894212 | 0.976205472 | T | -0.00586 | -0.1372 | 0.019112 | 5.38E-07 | *FPGT-TNNI3K* | 0 |  |
| 1 | 75002193 | rs12566985 | 0.985654205 | G | 0.006222 | 0.1421 | 0.01234 | 1.22E-07 | *FPGT-TNNI3K* | 0 |  |
| 1 | 75002667 | rs3845345 | 0.972716541 | G | -0.0057 | -0.1362 | 0.02269 | 6.50E-07 | *FPGT-TNNI3K* | 0 |  |
| 1 | 75003500 | rs12041852 | 0.987124127 | G | 0.006386 | 0.1423 | 0.010345 | 1.17E-07 | *FPGT-TNNI3K* | 0 |  |
| 1 | 75003710 | rs12041912 | 0.987151033 | G | 0.006374 | 0.1427 | 0.010494 | 1.08E-07 | *FPGT-TNNI3K* | 0 |  |
| 1 | 75004048 | rs3845347 | 0.987635824 | G | 0.006452 | 0.1423 | 0.009646 | 1.17E-07 | *FPGT-TNNI3K* | 0 |  |
| 1 | 75004611 | rs6604872 | 0.986025148 | T | 0.006266 | 0.1424 | 0.011955 | 1.15E-07 | *FPGT-TNNI3K* | 0 |  |
| 1 | 75004875 | rs10890130 | 0.974434658 | G | -0.00585 | -0.1358 | 0.019381 | 7.01E-07 | *FPGT-TNNI3K* | 0 |  |
| 1 | 75004943 | rs7520945 | 0.987419061 | T | 0.006429 | 0.1421 | 0.009848 | 1.22E-07 | *FPGT-TNNI3K* | 0 |  |
| 1 | 75005067 | rs7553348 | 0.986911624 | G | 0.006318 | 0.1432 | 0.011057 | 9.73E-08 | *FPGT-TNNI3K* | 0 |  |
| 1 | 75005238 | rs7553158 | 0.984642523 | G | 0.006083 | 0.1426 | 0.014435 | 9.83E-08 | *FPGT-TNNI3K* | 0 |  |
| 1 | 75005363 | rs11210477 | 0.976815204 | G | -0.00596 | -0.1369 | 0.016968 | 6.28E-07 | *FPGT-TNNI3K* | 0 |  |
| 1 | 75005776 | rs6656785 | 0.959417398 | A | -0.0059 | -0.1292 | 0.019726 | 2.80E-06 | *FPGT-TNNI3K* | 0 |  |
| 1 | 75006027 | rs3895907 | 0.986160526 | A | 0.006278 | 0.142 | 0.011553 | 1.24E-07 | *FPGT-TNNI3K* | 0 |  |
| 1 | 75006720 | rs7514705 | 0.987451329 | T | 0.00637 | 0.1441 | 0.010661 | 8.09E-08 | *FPGT-TNNI3K* | 0 |  |
| 1 | 75007008 | rs3765680 | 0.974895457 | A | -0.00575 | -0.1381 | 0.021734 | 5.00E-07 | *FPGT-TNNI3K* | 0 |  |
| 1 | 75008008 | rs6604871 | 0.975125469 | T | -0.00577 | -0.138 | 0.02121 | 5.10E-07 | *FPGT-TNNI3K* | 0 |  |
| 1 | 75008411 | rs11210478 | 0.976969897 | A | -0.0059 | -0.1381 | 0.018549 | 5.00E-07 | *FPGT-TNNI3K* | 0 |  |
| 1 | 75011358 | rs6669189 | 0.977159923 | C | -0.00597 | -0.138 | 0.017365 | 5.62E-07 | *FPGT-TNNI3K* | 1241 |  |
| 1 | 75012637 | rs10218727 | 0.970920396 | G | -0.00558 | -0.1381 | 0.027309 | 5.51E-07 | *FPGT-TNNI3K* | 2520 |  |
| 1 | 75013054 | rs3843262 | 0.978933185 | T | -0.00607 | -0.1409 | 0.017283 | 3.95E-07 | *FPGT-TNNI3K* | 2937 |  |
| 1 | 75014362 | rs10789396 | 0.987241636 | T | 0.006663 | 0.1612 | 0.010331 | 1.10E-07 | *FPGT-TNNI3K* | 4245 |  |
| 2 | 24942448 | rs11889662 | 0.951857001 | T | -0.0084 | 0.1419 | 0.005095 | 1.06E-05 | *NCOA1* | 0 | 2 |
| 2 | 24946188 | rs11693308 | 0.978392747 | C | -0.00887 | 0.1559 | 0.003236 | 2.93E-06 | *NCOA1* | 0 |  |
| 2 | 24991569 | rs17737058 | 0.955094717 | C | -0.008 | 0.1364 | 0.007608 | 1.30E-05 | *NCOA1* | 0 |  |
| 2 | 24996715 | rs17799110 | 0.950590342 | T | -0.00812 | 0.1381 | 0.006939 | 1.01E-05 | *NCOA1* | 3144 |  |
| 2 | 25079770 | rs11675457 | 0.958909087 | C | -0.00167 | -0.1537 | 0.506227 | 6.19E-09 | *ADCY3* | 0 | 3 |
| 2 | 25082926 | rs11687089 | 0.96564108 | T | -0.0022 | -0.1553 | 0.382016 | 6.46E-09 | *ADCY3* | 0 |  |
| 2 | 25083310 | rs7576788 | 0.956654116 | T | -0.00183 | -0.1449 | 0.46374 | 6.85E-08 | *ADCY3* | 0 |  |
| 2 | 25086827 | rs1529897 | 0.95363648 | T | -0.00125 | -0.1557 | 0.619381 | 3.93E-09 | *ADCY3* | 0 |  |
| 2 | 25096692 | rs11892869 | 0.963762786 | A | -0.00199 | -0.1626 | 0.425578 | 2.16E-09 | *ADCY3* | 0 |  |
| 2 | 25097072 | rs7580081 | 0.961834545 | G | -0.00182 | -0.1629 | 0.467462 | 6.27E-10 | *ADCY3* | 0 |  |
| 2 | 25099357 | rs6545776 | 0.963286261 | A | -0.00193 | -0.1621 | 0.439426 | 7.59E-10 | *ADCY3* | 0 |  |
| 2 | 25101092 | rs2033655 | 0.960869027 | G | -0.00182 | -0.1535 | 0.468202 | 6.47E-09 | *ADCY3* | 0 |  |
| 2 | 25135620 | rs2384061 | 0.953310636 | G | -0.00128 | -0.1516 | 0.608977 | 9.91E-09 | *ADCY3* | 0 |  |
| 2 | 25136866 | rs10203386 | 0.957917031 | T | -0.00151 | -0.162 | 0.54311 | 9.04E-10 | *ADCY3* | 0 |  |
| 2 | 25150116 | rs6752378 | 0.958194018 | C | -0.0015 | -0.1695 | 0.542756 | 1.05E-10 | *ADCY3* | 8060 |  |
| 2 | 25153986 | rs6726261 | 0.965530023 | T | 0.002206 | -0.1597 | 0.387423 | 4.73E-09 | *ADCY3* | 11930 |  |
| 2 | 25158281 | rs713587 | 0.952652053 | C | -0.0011 | -0.1646 | 0.657394 | 3.55E-10 | *DNAJC27* | 8222 |  |
| 2 | 25169200 | rs1172294 | 0.97116704 | A | -0.00254 | -0.1698 | 0.304086 | 2.21E-10 | *DNAJC27* | 0 |  |
| 2 | 25176200 | rs754536 | 0.957068192 | C | 0.001463 | 0.164 | 0.558148 | 1.36E-09 | *DNAJC27* | 0 |  |
| 2 | 25205427 | rs1982200 | 0.967735611 | C | 0.002269 | 0.1614 | 0.358968 | 8.97E-10 | *LOC729723* | 0 |  |
| 2 | 25286435 | rs1077492 | 0.970010399 | C | 0.002487 | 0.1648 | 0.321756 | 5.39E-10 | *EFR3B* | 0 | 4 |
| 4 | 130731284 | rs4864201 | 0.959658531 | T | 0.005524 | 0.1355 | 0.031964 | 1.41E-06 | *C4orf33* | 697440 | 5 |
| 12 | 50175380 | rs2720293 | 0.956903466 | A | -0.00287 | -0.1559 | 0.309445 | 3.70E-07 | *NCKAP5L* | 9547 | 6 |
| 12 | 50247468 | rs7138803 | 0.976659216 | G | -0.00315 | -0.1672 | 0.218313 | 6.50E-10 | *BCDIN3D* | 10555 |  |
| 12 | 50263148 | rs7132908 | 0.973909341 | G | -0.00453 | -0.1635 | 0.075927 | 1.24E-08 | *FAIM2* | 0 |  |
| 12 | 50285061 | rs297924 | 0.96581583 | T | -0.00479 | -0.1531 | 0.061394 | 1.20E-08 | *FAIM2* | 0 |  |
| 12 | 50285562 | rs17201502 | 0.970749344 | C | -0.00513 | -0.165 | 0.049669 | 2.51E-09 | *FAIM2* | 0 |  |
| 16 | 53799507 | rs9937053 | 0.961287087 | G | -0.00437 | -0.1776 | 0.077833 | 2.22E-10 | *FTO* | 0 | 7 |
| 16 | 53799905 | rs9928094 | 0.961518263 | A | -0.00438 | -0.1942 | 0.07735 | 7.20E-13 | *FTO* | 0 |  |
| 16 | 53799977 | rs9930333 | 0.962972766 | T | -0.00445 | -0.1776 | 0.072657 | 1.64E-10 | *FTO* | 0 |  |
| 16 | 53800568 | rs9939973 | 0.964533375 | G | -0.00452 | -0.1939 | 0.068124 | 6.39E-13 | *FTO* | 0 |  |
| 16 | 53800754 | rs9940128 | 0.965240343 | G | -0.00456 | -0.1942 | 0.066026 | 5.89E-13 | *FTO* | 0 |  |
| 16 | 53800954 | rs1421085 | 0.988083016 | T | -0.0057 | -0.191 | 0.02399 | 1.40E-12 | *FTO* | 0 |  |
| 16 | 53801549 | rs9923147 | 0.965983923 | C | -0.0046 | -0.1941 | 0.063846 | 6.06E-13 | *FTO* | 0 |  |
| 16 | 53801985 | rs9923544 | 0.967125682 | C | -0.00465 | -0.1942 | 0.060553 | 5.89E-13 | *FTO* | 0 |  |
| 16 | 53803574 | rs1558902 | 0.956743014 | T | -0.00395 | -0.1905 | 0.117897 | 1.32E-12 | *FTO* | 0 |  |
| 16 | 53805207 | rs11075985 | 0.96289184 | C | -0.00444 | -0.1957 | 0.073093 | 3.91E-13 | *FTO* | 0 |  |
| 16 | 53807764 | rs17817288 | 0.950651936 | A | -0.00391 | -0.1543 | 0.11088 | 7.07E-09 | *FTO* | 0 |  |
| 16 | 53809247 | rs1121980 | 0.973155873 | G | -0.00499 | -0.1961 | 0.044275 | 2.85E-13 | *FTO* | 0 |  |
| 16 | 53810686 | rs7193144 | 0.974605374 | T | -0.00514 | -0.1926 | 0.040648 | 4.96E-13 | *FTO* | 0 |  |
| 16 | 53812614 | rs8057044 | 0.959961977 | G | -0.00429 | -0.1637 | 0.081838 | 5.17E-10 | *FTO* | 0 |  |
| 16 | 53813367 | rs17817449 | 0.976482745 | T | -0.00526 | -0.1927 | 0.036123 | 3.93E-13 | *FTO* | 0 |  |
| 16 | 53813450 | rs8043757 | 0.974638448 | A | -0.00514 | -0.1928 | 0.040567 | 3.82E-13 | *FTO* | 0 |  |
| 16 | 53816275 | rs8050136 | 0.976535177 | C | -0.00527 | -0.1909 | 0.035999 | 5.27E-13 | *FTO* | 0 |  |
| 16 | 53816752 | rs8051591 | 0.975474306 | A | -0.00519 | -0.1932 | 0.03853 | 2.77E-13 | *FTO* | 0 |  |
| 16 | 53816838 | rs9935401 | 0.976826134 | G | -0.00528 | -0.1935 | 0.035317 | 2.54E-13 | *FTO* | 0 |  |
| 16 | 53818460 | rs3751812 | 0.992081098 | G | -0.00631 | -0.1932 | 0.012922 | 2.77E-13 | *FTO* | 0 |  |
| 16 | 53818708 | rs3751813 | 0.965678091 | G | -0.00455 | -0.166 | 0.064313 | 6.39E-10 | *FTO* | 0 |  |
| 16 | 53819169 | rs9936385 | 0.978661318 | T | -0.00541 | -0.184 | 0.031072 | 5.77E-11 | *FTO* | 0 |  |
| 16 | 53819198 | rs9923233 | 0.978195312 | G | -0.00538 | -0.1838 | 0.032142 | 2.24E-11 | *FTO* | 0 |  |
| 16 | 53819877 | rs11075989 | 0.978033745 | C | -0.00537 | -0.1781 | 0.032473 | 7.70E-11 | *FTO* | 0 |  |
| 16 | 53819893 | rs11075990 | 0.977998845 | A | -0.00537 | -0.1934 | 0.032613 | 2.62E-13 | *FTO* | 0 |  |
| 16 | 53820527 | rs9939609 | 0.977799987 | T | -0.00535 | -0.1934 | 0.033066 | 2.62E-13 | *FTO* | 0 |  |
| 16 | 53821615 | rs7202116 | 0.985475737 | A | -0.00537 | -0.1892 | 0.032387 | 1.39E-11 | *FTO* | 0 |  |
| 16 | 53821862 | rs7201850 | 0.976619974 | C | -0.00523 | -0.1831 | 0.035769 | 2.66E-11 | *FTO* | 0 |  |
| 16 | 53822651 | rs7185735 | 0.977521824 | A | -0.00533 | -0.1927 | 0.033703 | 3.93E-13 | *FTO* | 0 |  |
| 16 | 53825488 | rs9941349 | 0.986959151 | C | -0.00612 | -0.1978 | 0.014687 | 1.16E-13 | *FTO* | 0 |  |
| 16 | 53827179 | rs9931494 | 0.98849514 | C | -0.00629 | -0.1974 | 0.012143 | 1.30E-13 | *FTO* | 0 |  |
| 16 | 53828066 | rs17817964 | 0.989033776 | C | -0.00643 | -0.1933 | 0.011291 | 4.09E-13 | *FTO* | 0 |  |
| 16 | 53828752 | rs7190492 | 0.969030221 | A | -0.00383 | -0.1359 | 0.132081 | 6.88E-07 | *FTO* | 0 |  |
| 16 | 53830452 | rs9930501 | 0.971759749 | A | -0.00492 | -0.1881 | 0.04787 | 1.70E-12 | *FTO* | 0 |  |
| 16 | 53830465 | rs9930506 | 0.967304585 | A | -0.00468 | -0.1874 | 0.060038 | 2.05E-12 | *FTO* | 0 |  |
| 16 | 53830491 | rs9932754 | 0.97186551 | T | -0.00493 | -0.1868 | 0.047593 | 1.98E-12 | *FTO* | 0 |  |
| 16 | 53831146 | rs9922708 | 0.967732734 | C | -0.00471 | -0.1866 | 0.058824 | 2.09E-12 | *FTO* | 0 |  |
| 16 | 53831771 | rs9922619 | 0.971705106 | G | -0.00493 | -0.1866 | 0.048012 | 2.09E-12 | *FTO* | 0 |  |
| 16 | 53842908 | rs12149832 | 0.969009936 | G | -0.00484 | -0.1837 | 0.055251 | 5.49E-12 | *FTO* | 0 |  |
| 16 | 53845487 | rs11642841 | 0.982472194 | C | -0.00583 | -0.1882 | 0.023015 | 2.48E-11 | *FTO* | 0 |  |
| 16 | 53848561 | rs1861867 | 0.955645547 | A | -0.00295 | -0.1742 | 0.268237 | 5.93E-07 | *FTO* | 0 |  |
| 18 | 38765542 | rs17697435 | 0.976022614 | T | -0.01002 | -0.1859 | 0.008312 | 1.87E-06 | *KC6* | 294692 | 8 |
| 18 | 38765623 | rs17697453 | 0.975370915 | A | -0.01001 | -0.1854 | 0.008322 | 2.00E-06 | *KC6* | 294611 |  |
| 18 | 38765659 | rs17697518 | 0.977425349 | C | -0.0102 | -0.1855 | 0.007197 | 1.85E-06 | *KC6* | 294575 |  |

**Table S6: Replication and validation of our findings in the European birthweight (2013 GWAS) and childhood obesity (2013 GWAS) with European birthweight (2013 GWAS) and childhood obesity (2019 GWAS)**

| **European birthweight (BW) & Childhood obesity (COB)** | | | | | | | | | | | | |
| --- | --- | --- | --- | --- | --- | --- | --- | --- | --- | --- | --- | --- |
| **Chr** | **Position (hg19)** | **SNP** | **Effect Allele** | **Beta (BW)** | **Beta (COB)** | **P value (BW)** | **P value (COB)** | **Nearest gene** | **Distance to nearest gene** | **Loci** | **Joint Posterior Probability (2013 COB GWAS)** | **Joint Posterior Probability (2019 COB GWAS)** |
| 1 | 74977277 | rs6690871 | A | -0.00677 | -0.1493 | 0.009442 | 3.89E-08 | *FPGT-TNNI3K* | 0 | 1 | 0.997407904 | 0.999614035 |
| 1 | 74977425 | rs953567 | A | -0.00653 | -0.1339 | 0.012666 | 2.86E-06 | *FPGT-TNNI3K* | 0 |  | 0.976787093 | 0.999584987 |
| 1 | 74977870 | rs1040070 | G | 0.006294 | 0.1492 | 0.014694 | 2.78E-08 | *FPGT-TNNI3K* | 0 |  | 0.997033229 | 0.99963977 |
| 1 | 74979975 | rs12036473 | A | -0.00497 | -0.133 | 0.05853 | 1.29E-06 | *FPGT-TNNI3K* | 0 |  | 0.97301915 | 0.999596613 |
| 1 | 74983835 | rs10493544 | T | 0.006962 | 0.1469 | 0.006852 | 3.15E-08 | *FPGT-TNNI3K* | 0 |  | 0.997908801 | 0.999598296 |
| 1 | 74991402 | rs1514177 | C | 0.006157 | 0.1348 | 0.016617 | 3.83E-07 | *FPGT-TNNI3K* | 0 |  | 0.991714514 | 0.999499387 |
| 1 | 74991596 | rs1514176 | G | 0.00714 | 0.1349 | 0.005471 | 3.75E-07 | *FPGT-TNNI3K* | 0 |  | 0.994395843 | 0.999532936 |
| 1 | 74991644 | rs1514175 | A | 0.007041 | 0.136 | 0.006124 | 3.02E-07 | *FPGT-TNNI3K* | 0 |  | 0.991800667 | 0.999472784 |
| 1 | 74992278 | rs6604867 | T | 0.007264 | 0.1444 | 0.004627 | 5.36E-08 | *FPGT-TNNI3K* | 0 |  | 0.997880948 | 0.999886645 |
| 1 | 74992546 | rs6604866 | G | 0.00704 | 0.145 | 0.006067 | 4.72E-08 | *FPGT-TNNI3K* | 0 |  | 0.997742079 | 0.999872258 |
| 1 | 74993063 | rs1514174 | C | 0.006992 | 0.1451 | 0.006428 | 4.62E-08 | *FPGT-TNNI3K* | 0 |  | 0.997702368 | 0.999864303 |
| 1 | 74993318 | rs7526762 | A | 0.007104 | 0.1449 | 0.005627 | 4.82E-08 | *FPGT-TNNI3K* | 0 |  | 0.997795987 | 0.999761918 |
| 1 | 74995110 | rs1514173 | C | -0.00626 | -0.1381 | 0.015726 | 3.71E-07 | *FPGT-TNNI3K* | 0 |  | 0.992022531 | 0.999886544 |
| 1 | 74995225 | rs7551507 | C | 0.007228 | 0.1432 | 0.004831 | 7.75E-08 | *FPGT-TNNI3K* | 0 |  | 0.997502654 | 0.999857338 |
| 1 | 74997762 | rs12042908 | A | 0.007255 | 0.1435 | 0.004704 | 7.28E-08 | *FPGT-TNNI3K* | 0 |  | 0.997590427 | 0.999909206 |
| 1 | 74997795 | rs6703637 | T | -0.00622 | -0.1374 | 0.01647 | 5.18E-07 | *FPGT-TNNI3K* | 0 |  | 0.990376812 | 0.999904941 |
| 1 | 74997956 | rs6698622 | G | -0.00633 | -0.1374 | 0.01463 | 5.18E-07 | *FPGT-TNNI3K* | 0 |  | 0.990801701 | 0.999915191 |
| 1 | 74999713 | rs2344508 | G | 0.007242 | 0.1426 | 0.004759 | 1.10E-07 | *FPGT-TNNI3K* | 0 |  | 0.997104367 | 0.999905309 |
| 1 | 75000011 | rs12142020 | A | -0.00629 | -0.1367 | 0.038996 | 5.91E-07 | *FPGT-TNNI3K* | 0 |  | 0.985126181 | 0.999889489 |
| 1 | 75001683 | rs3894212 | T | -0.00625 | -0.1372 | 0.015847 | 5.38E-07 | *FPGT-TNNI3K* | 0 |  | 0.990329548 | 0.999895381 |
| 1 | 75002193 | rs12566985 | G | 0.007077 | 0.1421 | 0.005805 | 1.22E-07 | *FPGT-TNNI3K* | 0 |  | 0.996752044 | 0.99984028 |
| 1 | 75002667 | rs3845345 | G | -0.00624 | -0.1362 | 0.016022 | 6.50E-07 | *FPGT-TNNI3K* | 0 |  | 0.989266527 | 0.999913689 |
| 1 | 75003500 | rs12041852 | G | 0.007172 | 0.1423 | 0.005179 | 1.17E-07 | *FPGT-TNNI3K* | 0 |  | 0.996934863 | 0.99991462 |
| 1 | 75003710 | rs12041912 | G | 0.007153 | 0.1427 | 0.005299 | 1.08E-07 | *FPGT-TNNI3K* | 0 |  | 0.997022349 | 0.999857857 |
| 1 | 75004048 | rs3845347 | G | 0.007244 | 0.1423 | 0.004786 | 1.17E-07 | *FPGT-TNNI3K* | 0 |  | 0.997015729 | 0.999864544 |
| 1 | 75004611 | rs6604872 | T | 0.007172 | 0.1424 | 0.005177 | 1.15E-07 | *FPGT-TNNI3K* | 0 |  | 0.996963996 | 0.999914215 |
| 1 | 75004875 | rs10890130 | G | -0.00621 | -0.1358 | 0.016624 | 7.01E-07 | *FPGT-TNNI3K* | 0 |  | 0.988667136 | 0.999798174 |
| 1 | 75004943 | rs7520945 | T | 0.007176 | 0.1421 | 0.005154 | 1.22E-07 | *FPGT-TNNI3K* | 0 |  | 0.996883128 | 0.999860069 |
| 1 | 75005067 | rs7553348 | G | 0.007136 | 0.1432 | 0.005402 | 9.73E-08 | *FPGT-TNNI3K* | 0 |  | 0.997134033 | 0.999906994 |
| 1 | 75005238 | rs7553158 | G | 0.007053 | 0.1426 | 0.005962 | 9.83E-08 | *FPGT-TNNI3K* | 0 |  | 0.9970154 | 0.999859442 |
| 1 | 75005363 | rs11210477 | G | -0.00617 | -0.1369 | 0.017358 | 6.28E-07 | *FPGT-TNNI3K* | 0 |  | 0.989139965 | 0.999911213 |
| 1 | 75005776 | rs6656785 | A | -0.00617 | -0.1292 | 0.017929 | 2.80E-06 | *FPGT-TNNI3K* | 0 |  | 0.974307034 | 0.999910381 |
| 1 | 75006027 | rs3895907 | A | 0.006993 | 0.142 | 0.006433 | 1.24E-07 | *FPGT-TNNI3K* | 0 |  | 0.996599604 | 0.999914017 |
| 1 | 75006720 | rs7514705 | T | 0.007113 | 0.1441 | 0.005628 | 8.09E-08 | *FPGT-TNNI3K* | 0 |  | 0.997309857 | 0.999911227 |
| 1 | 75007008 | rs3765680 | A | -0.00611 | -0.1381 | 0.018595 | 5.00E-07 | *FPGT-TNNI3K* | 0 |  | 0.990084348 | 0.999857317 |
| 1 | 75008008 | rs6604871 | T | -0.0059 | -0.138 | 0.023186 | 5.10E-07 | *FPGT-TNNI3K* | 0 |  | 0.989035897 | 0.999911387 |
| 1 | 75008411 | rs11210478 | A | -0.00605 | -0.1381 | 0.019957 | 5.00E-07 | *FPGT-TNNI3K* | 0 |  | 0.989796592 | 0.999909677 |
| 1 | 75011358 | rs6669189 | C | -0.00624 | -0.138 | 0.016584 | 5.62E-07 | *FPGT-TNNI3K* | 1241 |  | 0.989932151 | 0.99989286 |
| 1 | 75012637 | rs10218727 | G | -0.00596 | -0.1381 | 0.022661 | 5.51E-07 | *FPGT-TNNI3K* | 2520 |  | 0.988699919 | 0.999833654 |
| 1 | 75013054 | rs3843262 | T | -0.00606 | -0.1409 | 0.021862 | 3.95E-07 | *FPGT-TNNI3K* | 2937 |  | 0.990570538 | 0.999831412 |
| 1 | 75014362 | rs10789396 | T | 0.007703 | 0.1612 | 0.004172 | 1.10E-07 | *FPGT-TNNI3K* | 4245 |  | 0.997232469 | 0.999903026 |
| 1 | 177852580 | rs633715 | T | 0.00407 | -0.1699 | 0.19203 | 2.08E-07 | *SEC16B* | 45660 | 2 | 0.977654408 | 0.999908158 |
| 1 | 177873210 | rs574367 | G | 0.004404 | -0.1741 | 0.159993 | 1.24E-07 | *SEC16B* | 25030 |  | 0.983031603 | 0.999850665 |
| 1 | 177875514 | rs527248 | A | 0.004383 | -0.1735 | 0.16206 | 1.37E-07 | *SEC16B* | 22726 |  | 0.982374864 | 0.999836642 |
| 1 | 177876946 | rs589500 | C | 0.004516 | -0.1737 | 0.14932 | 1.33E-07 | *SEC16B* | 21294 |  | 0.983374161 | 0.999846309 |
| 1 | 177881651 | rs693232 | C | 0.004533 | -0.1738 | 0.147811 | 1.31E-07 | *SEC16B* | 16589 |  | 0.983558143 | 0.999857457 |
| 1 | 177889480 | rs543874 | A | 0.003614 | -0.178 | 0.252343 | 8.54E-08 | *SEC16B* | 8760 |  | 0.971279422 | 0.999838157 |
| 1 | 177894287 | rs506589 | T | 0.003229 | -0.1782 | 0.306948 | 8.27E-08 | *SEC16B* | 3953 |  | 0.977485766 | 0.999840503 |
| 1 | 177913519 | rs10913469 | T | 0.002594 | -0.1773 | 0.413486 | 7.99E-08 | *SEC16B* | 0 |  | 0.973174914 | 0.999918687 |
| 2 | 600575 | rs2683992 | G | -0.00227 | -0.224 | 0.497712 | 1.96E-10 | *TMEM18* | 67396 | 3 | 0.980391467 | 0.999832133 |
| 2 | 601905 | rs2867105 | T | -0.00214 | -0.2241 | 0.522932 | 1.03E-10 | *TMEM18* | 66066 |  | 0.9798236 | 0.999854458 |
| 2 | 610603 | rs2867131 | T | -0.00248 | -0.2341 | 0.465755 | 4.87E-11 | *TMEM18* | 57368 |  | 0.981331476 | 0.96236504 |
| 2 | 614168 | rs2947411 | A | -0.00301 | -0.2381 | 0.37012 | 1.16E-11 | *TMEM18* | 53803 |  | 0.983961917 | 0.999162097 |
| 2 | 614210 | rs2860323 | A | -0.00243 | -0.2379 | 0.474072 | 1.21E-11 | *TMEM18* | 53761 |  | 0.981161767 | 0.999267815 |
| 2 | 615140 | rs7567570 | T | -0.00313 | -0.223 | 0.352679 | 8.31E-10 | *TMEM18* | 52831 |  | 0.984058185 | 0.999263941 |
| 2 | 621461 | rs6548237 | A | -0.00126 | -0.239 | 0.709728 | 1.11E-11 | *TMEM18* | 46510 |  | 0.975541378 | 0.999406359 |
| 2 | 621558 | rs939584 | C | -0.00257 | -0.191 | 0.444212 | 2.49E-08 | *TMEM18* | 46413 |  | 0.977334826 | 0.99938645 |
| 2 | 622161 | rs1320331 | G | -0.0025 | -0.2431 | 0.457339 | 4.28E-12 | *TMEM18* | 45810 |  | 0.981610728 | 0.999409369 |
| 2 | 622225 | rs1320330 | T | -0.0025 | -0.2383 | 0.457325 | 1.28E-11 | *TMEM18* | 45746 |  | 0.981595305 | 0.999460113 |
| 2 | 622531 | rs939583 | C | -0.00239 | -0.2422 | 0.477704 | 5.12E-12 | *TMEM18* | 45440 |  | 0.981081282 | 0.999389156 |
| 2 | 622723 | rs939582 | A | -0.00243 | -0.2279 | 0.471553 | 3.52E-10 | *TMEM18* | 45248 |  | 0.980974226 | 0.999374483 |
| 2 | 622827 | rs2867125 | T | -0.00238 | -0.24 | 0.480064 | 7.95E-12 | *TMEM18* | 45144 |  | 0.981014877 | 0.999417732 |
| 2 | 623691 | rs11127483 | G | -0.00247 | -0.2256 | 0.463407 | 4.21E-10 | *TMEM18* | 44280 |  | 0.981152452 | 0.999416374 |
| 2 | 623798 | rs11127484 | T | -0.00247 | -0.2409 | 0.464435 | 6.65E-12 | *TMEM18* | 44173 |  | 0.981420902 | 0.853724103 |
| 2 | 623935 | rs6719518 | C | -0.00237 | -0.2419 | 0.482858 | 6.27E-12 | *TMEM18* | 44036 |  | 0.98094661 | 0.999317094 |
| 2 | 623976 | rs6728726 | T | -0.00208 | -0.2461 | 0.536653 | 2.10E-11 | *TMEM18* | 43995 |  | 0.979570089 | 0.999415873 |
| 2 | 624034 | rs6711012 | G | -0.0021 | -0.242 | 0.533207 | 6.15E-12 | *TMEM18* | 43937 |  | 0.97968107 | 0.857312775 |
| 2 | 624524 | rs2867123 | G | -0.00141 | -0.2418 | 0.675221 | 6.40E-12 | *TMEM18* | 43447 |  | 0.976326577 | 0.999406256 |
| 2 | 624581 | rs2867122 | A | -0.00242 | -0.2418 | 0.472859 | 6.40E-12 | *TMEM18* | 43390 |  | 0.981203349 | 0.999405922 |
| 2 | 624678 | rs2903492 | G | -0.00243 | -0.242 | 0.470825 | 6.15E-12 | *TMEM18* | 43293 |  | 0.981256382 | 0.999300386 |
| 2 | 625029 | rs7576624 | C | -0.00241 | -0.242 | 0.474571 | 6.15E-12 | *TMEM18* | 42942 |  | 0.981159722 | 0.999403422 |
| 2 | 625057 | rs7576635 | C | -0.00243 | -0.242 | 0.470558 | 6.15E-12 | *TMEM18* | 42914 |  | 0.981263282 | 0.999416519 |
| 2 | 628504 | rs6744646 | A | -0.00166 | -0.243 | 0.623421 | 5.80E-12 | *TMEM18* | 39467 |  | 0.977518199 | 0.999415877 |
| 2 | 628524 | rs6744653 | A | -0.00166 | -0.243 | 0.622676 | 5.80E-12 | *TMEM18* | 39447 |  | 0.977535575 | 0.999415825 |
| 2 | 629244 | rs12463617 | A | -0.00156 | -0.2278 | 0.644556 | 6.83E-09 | *TMEM18* | 38727 |  | 0.974624601 | 0.999333933 |
| 2 | 629510 | rs6743060 | C | -0.00186 | -0.244 | 0.583031 | 4.75E-12 | *TMEM18* | 38461 |  | 0.978474199 | 0.999319708 |
| 2 | 629694 | rs6752470 | T | -0.00093 | -0.244 | 0.78289 | 4.75E-12 | *TMEM18* | 38277 |  | 0.973960512 | 0.999421358 |
| 2 | 629881 | rs12995480 | T | -0.00168 | -0.2436 | 0.618608 | 5.15E-12 | *TMEM18* | 38090 |  | 0.977632231 | 0.999420023 |
| 2 | 629914 | rs6732471 | G | -0.00183 | -0.2436 | 0.588664 | 5.15E-12 | *TMEM18* | 38057 |  | 0.978338825 | 0.999438952 |
| 2 | 630024 | rs13007080 | A | -0.00182 | -0.2436 | 0.590015 | 5.15E-12 | *TMEM18* | 37947 |  | 0.978306665 | 0.999295071 |
| 2 | 630034 | rs13007086 | A | -0.00197 | -0.244 | 0.561265 | 4.75E-12 | *TMEM18* | 37937 |  | 0.978997983 | 0.999312607 |
| 2 | 630323 | rs6725549 | C | -0.00179 | -0.2297 | 0.596692 | 2.87E-10 | *TMEM18* | 37648 |  | 0.977885552 | 0.999400734 |
| 2 | 630339 | rs6731348 | A | -0.00168 | -0.2439 | 0.619218 | 4.85E-12 | *TMEM18* | 37632 |  | 0.977618742 | 0.99939779 |
| 2 | 630662 | rs6731688 | A | -0.0018 | -0.2266 | 0.595023 | 4.96E-10 | *TMEM18* | 37309 |  | 0.977802215 | 0.999408534 |
| 2 | 630995 | rs5017303 | T | -0.00175 | -0.2408 | 0.605236 | 8.97E-12 | *TMEM18* | 36976 |  | 0.977937444 | 0.999398883 |
| 2 | 631099 | rs5017300 | C | -0.00166 | -0.2408 | 0.623477 | 8.97E-12 | *TMEM18* | 36872 |  | 0.97750965 | 0.999386152 |
| 2 | 631528 | rs7585056 | A | -0.00182 | -0.2404 | 0.591117 | 9.70E-12 | *TMEM18* | 36443 |  | 0.97827033 | 0.999285439 |
| 2 | 632028 | rs11127485 | C | -0.00214 | -0.2404 | 0.526887 | 9.70E-12 | *TMEM18* | 35943 |  | 0.979830275 | 0.999376645 |
| 2 | 632146 | rs12623218 | T | -0.00171 | -0.2418 | 0.613876 | 7.36E-12 | *TMEM18* | 35825 |  | 0.977737748 | 0.999372516 |
| 2 | 632300 | rs12992154 | G | -0.00169 | -0.2264 | 0.616553 | 5.14E-10 | *TMEM18* | 35671 |  | 0.977277169 | 0.999387647 |
| 2 | 632348 | rs13021737 | A | -0.00176 | -0.2263 | 0.603196 | 5.23E-10 | *TMEM18* | 35623 |  | 0.977591319 | 0.99938742 |
| 2 | 632550 | rs13012571 | C | -0.00184 | -0.24 | 0.585646 | 9.14E-12 | *TMEM18* | 35421 |  | 0.978401831 | 0.999387572 |
| 2 | 634905 | rs6548238 | T | -0.00163 | -0.2452 | 0.629178 | 2.82E-11 | *TMEM18* | 33066 |  | 0.977343856 | 0.999323887 |
| 2 | 635200 | rs6734363 | G | -0.00168 | -0.2397 | 0.620023 | 9.70E-12 | *TMEM18* | 32771 |  | 0.977588758 | 0.999349842 |
| 2 | 635721 | rs6755502 | T | -0.00171 | -0.2386 | 0.613138 | 1.05E-11 | *TMEM18* | 32250 |  | 0.977748395 | 0.999280136 |
| 2 | 637597 | rs13388043 | C | -0.00194 | -0.2399 | 0.567072 | 8.11E-12 | *TMEM18* | 30374 |  | 0.978849889 | 0.999387524 |
| 2 | 637830 | rs13393304 | A | -0.00162 | -0.2401 | 0.630495 | 7.80E-12 | *TMEM18* | 30141 |  | 0.977348865 | 0.999402868 |
| 2 | 638144 | rs4854344 | G | -0.00143 | -0.2445 | 0.671334 | 3.22E-12 | *TMEM18* | 29827 |  | 0.976423461 | 0.999384661 |
| 2 | 642499 | rs7601028 | C | -0.0015 | -0.2517 | 0.656923 | 6.16E-11 | *TMEM18* | 25472 |  | 0.97665986 | 0.999304557 |
| 2 | 643303 | rs7604609 | C | -0.00151 | -0.2565 | 0.65436 | 1.90E-09 | *TMEM18* | 24668 |  | 0.975780769 | 0.999378298 |
| 2 | 644953 | rs7561317 | A | -0.0013 | -0.2412 | 0.700916 | 6.26E-12 | *TMEM18* | 23018 |  | 0.975749119 | 0.999388636 |
| 2 | 646145 | rs11127491 | T | -0.00148 | -0.24 | 0.660208 | 9.14E-12 | *TMEM18* | 21826 |  | 0.97666157 | 0.999269153 |
| 2 | 646364 | rs10189761 | T | -0.0006 | -0.2363 | 0.857934 | 1.65E-11 | *TMEM18* | 21607 |  | 0.972350686 | 0.99940142 |
| 2 | 646674 | rs10190052 | T | -0.00144 | -0.2413 | 0.670534 | 7.07E-12 | *TMEM18* | 21297 |  | 0.976431206 | 0.999434949 |
| 2 | 646767 | rs10173167 | G | -0.0014 | -0.2413 | 0.678023 | 7.07E-12 | *TMEM18* | 21204 |  | 0.976261528 | 0.99943687 |
| 2 | 646803 | rs7571957 | T | -0.00145 | -0.2412 | 0.66848 | 7.21E-12 | *TMEM18* | 21168 |  | 0.976477527 | 0.883566086 |
| 2 | 647580 | rs10193244 | T | -0.0014 | -0.2416 | 0.678223 | 6.66E-12 | *TMEM18* | 20391 |  | 0.97625801 | 0.999408734 |
| 2 | 647760 | rs4854348 | A | -0.00138 | -0.2415 | 0.682607 | 6.79E-12 | *TMEM18* | 20211 |  | 0.976158687 | 0.999395746 |
| 2 | 647861 | rs4854349 | T | -0.00142 | -0.2415 | 0.674856 | 6.79E-12 | *TMEM18* | 20110 |  | 0.976333869 | 0.999385315 |
| 2 | 648198 | rs7570198 | C | -0.00187 | -0.2415 | 0.575703 | 6.79E-12 | *TMEM18* | 19773 |  | 0.9786449 | 0.999539228 |
| 2 | 648758 | rs4423631 | T | -0.00174 | -0.2414 | 0.604167 | 6.93E-12 | *TMEM18* | 19213 |  | 0.977967121 | 0.99941627 |
| 2 | 648810 | rs4452188 | A | 0.001394 | 0.2414 | 0.679519 | 6.93E-12 | *TMEM18* | 19161 |  | 0.976228055 | 0.999418755 |
| 2 | 649347 | rs1320338 | T | 0.001358 | 0.2413 | 0.687327 | 7.07E-12 | *TMEM18* | 18624 |  | 0.97605169 | 0.99956735 |
| 2 | 649867 | rs1320337 | A | 0.001319 | 0.2416 | 0.695986 | 6.66E-12 | *TMEM18* | 18104 |  | 0.975858361 | 0.999539519 |
| 2 | 650012 | rs1320336 | G | 0.001255 | 0.2288 | 0.710103 | 3.77E-10 | *TMEM18* | 17959 |  | 0.975195297 | 0.999577867 |
| 2 | 650143 | rs2867108 | T | 0.001362 | 0.2426 | 0.686613 | 6.28E-12 | *TMEM18* | 17828 |  | 0.976069713 | 0.999497345 |
| 2 | 650479 | rs13386517 | G | 0.001409 | 0.2426 | 0.676399 | 6.28E-12 | *TMEM18* | 17492 |  | 0.976300205 | 0.999599913 |
| 2 | 650519 | rs13401686 | A | 0.001196 | 0.2434 | 0.723077 | 6.16E-12 | *TMEM18* | 17452 |  | 0.975257317 | 0.999543585 |
| 2 | 650560 | rs13386627 | G | 0.001336 | 0.2434 | 0.692168 | 6.16E-12 | *TMEM18* | 17411 |  | 0.975945186 | 0.999550343 |
| 2 | 650647 | rs4613321 | A | 0.001357 | 0.2432 | 0.687727 | 6.41E-12 | *TMEM18* | 17324 |  | 0.976044312 | 0.999616176 |
| 2 | 650828 | rs13386964 | G | 0.001367 | 0.2424 | 0.685423 | 7.51E-12 | *TMEM18* | 17143 |  | 0.976093466 | 0.999609044 |
| 2 | 651030 | rs2867109 | T | 0.001378 | 0.2427 | 0.683139 | 7.08E-12 | *TMEM18* | 16941 |  | 0.976145977 | 0.999548711 |
| 2 | 651105 | rs2867110 | G | 0.001398 | 0.2425 | 0.678679 | 7.37E-12 | *TMEM18* | 16866 |  | 0.976245973 | 0.999549689 |
| 2 | 651349 | rs2867112 | T | 0.001846 | 0.2424 | 0.59319 | 7.51E-12 | *TMEM18* | 16622 |  | 0.97822566 | 0.999541137 |
| 2 | 651365 | rs2867113 | G | 0.004095 | 0.2424 | 0.289092 | 7.51E-12 | *TMEM18* | 16606 |  | 0.986352551 | 0.999574586 |
| 2 | 651407 | rs12714414 | T | 0.002307 | 0.2428 | 0.521881 | 6.94E-12 | *TMEM18* | 16564 |  | 0.979960164 | 0.999495135 |
| 2 | 651430 | rs12714415 | T | 0.001586 | 0.243 | 0.648616 | 6.67E-12 | *TMEM18* | 16541 |  | 0.976932811 | 0.999335039 |
| 2 | 651507 | rs6719980 | T | 0.001366 | 0.243 | 0.686363 | 6.67E-12 | *TMEM18* | 16464 |  | 0.976074352 | 0.999431506 |
| 2 | 652247 | rs7608050 | G | 0.001445 | 0.2429 | 0.669467 | 7.82E-12 | *TMEM18* | 15724 |  | 0.976453648 | 0.999419966 |
| 2 | 652542 | rs7574359 | T | 0.001215 | 0.2428 | 0.720126 | 7.98E-12 | *TMEM18* | 15429 |  | 0.975317959 | 0.99944664 |
| 2 | 653093 | rs13415094 | T | 0.00134 | 0.2434 | 0.69221 | 8.14E-12 | *TMEM18* | 14878 |  | 0.975939406 | 0.999496007 |
| 2 | 653195 | rs13396935 | G | 0.001583 | 0.2436 | 0.640172 | 7.83E-12 | *TMEM18* | 14776 |  | 0.977124661 | 0.999351079 |
| 2 | 653354 | rs13397165 | G | 0.001265 | 0.2437 | 0.708976 | 7.68E-12 | *TMEM18* | 14617 |  | 0.97556596 | 0.999505802 |
| 2 | 653874 | rs10188334 | C | 0.001583 | 0.2304 | 0.641969 | 4.47E-10 | *TMEM18* | 14097 |  | 0.976715257 | 0.999233881 |
| 2 | 24942448 | rs11889662 | T | -0.00777 | 0.1419 | 0.011406 | 1.06E-05 | *NCOA1* | 0 | 4 | 0.950032127 | 0.999368219 |
| 2 | 24946188 | rs11693308 | C | -0.00804 | 0.1559 | 0.008983 | 2.93E-06 | *NCOA1* | 0 |  | 0.978702965 | 0.999450727 |
| 2 | 24991569 | rs17737058 | C | -0.00751 | 0.1364 | 0.014678 | 1.30E-05 | *NCOA1* | 0 |  | 0.952050281 | 0.999450734 |
| 2 | 25075281 | rs7608976 | G | -0.00039 | -0.1609 | 0.88235 | 2.40E-09 | *ADCY3* | 0 | 5 | 0.970449095 | 0.999440911 |
| 2 | 25075675 | rs7597332 | A | -0.00024 | -0.1559 | 0.927462 | 2.55E-08 | *ADCY3* | 0 |  | 0.963829851 | 0.999443849 |
| 2 | 25076126 | rs13387729 | A | -0.0002 | -0.1605 | 0.939578 | 1.72E-09 | *ADCY3* | 0 |  | 0.969536515 | 0.999347716 |
| 2 | 25079770 | rs11675457 | C | -0.00154 | -0.1537 | 0.553621 | 6.19E-09 | *ADCY3* | 0 |  | 0.977119721 | 0.999357126 |
| 2 | 25082273 | rs916485 | T | -0.00135 | -0.1605 | 0.600414 | 1.29E-09 | *ADCY3* | 0 |  | 0.97731107 | 0.999447278 |
| 2 | 25082414 | rs1541984 | G | -0.00157 | -0.153 | 0.546743 | 8.27E-09 | *ADCY3* | 0 |  | 0.976861676 | 0.999438466 |
| 2 | 25082926 | rs11687089 | T | -0.00202 | -0.1553 | 0.438356 | 6.46E-09 | *ADCY3* | 0 |  | 0.980247722 | 0.999413826 |
| 2 | 25083310 | rs7576788 | T | -0.00138 | -0.1449 | 0.593907 | 6.85E-08 | *ADCY3* | 0 |  | 0.967578687 | 0.999415359 |
| 2 | 25086827 | rs1529897 | T | -0.00125 | -0.1557 | 0.629631 | 3.93E-09 | *ADCY3* | 0 |  | 0.975739438 | 0.999356451 |
| 2 | 25096692 | rs11892869 | A | -0.00163 | -0.1626 | 0.527899 | 2.16E-09 | *ADCY3* | 0 |  | 0.978825836 | 0.999443223 |
| 2 | 25096952 | rs7567997 | T | -0.00162 | -0.163 | 0.530932 | 6.12E-10 | *ADCY3* | 0 |  | 0.97932156 | 0.999352911 |
| 2 | 25097072 | rs7580081 | G | -0.00153 | -0.1629 | 0.554437 | 6.27E-10 | *ADCY3* | 0 |  | 0.978727175 | 0.999442405 |
| 2 | 25097644 | rs13407913 | A | -0.0016 | -0.1623 | 0.53669 | 8.42E-10 | *ADCY3* | 0 |  | 0.979072936 | 0.999353062 |
| 2 | 25097939 | rs13410999 | T | -0.00161 | -0.1624 | 0.532055 | 7.07E-10 | *ADCY3* | 0 |  | 0.979249293 | 0.999376999 |
| 2 | 25099357 | rs6545776 | A | -0.00168 | -0.1621 | 0.516245 | 7.59E-10 | *ADCY3* | 0 |  | 0.979626897 | 0.999377302 |
| 2 | 25100328 | rs2384058 | A | -0.00153 | -0.1633 | 0.55386 | 5.69E-10 | *ADCY3* | 0 |  | 0.978769659 | 0.999368497 |
| 2 | 25100338 | rs2384059 | C | -0.00153 | -0.1622 | 0.554626 | 7.41E-10 | *ADCY3* | 0 |  | 0.978668697 | 0.999393735 |
| 2 | 25100738 | rs10865315 | C | -0.00148 | -0.1545 | 0.569162 | 5.16E-09 | *ADCY3* | 0 |  | 0.976950211 | 0.999316555 |
| 2 | 25100902 | rs2033656 | C | -0.0015 | -0.1618 | 0.560385 | 8.16E-10 | *ADCY3* | 0 |  | 0.978492295 | 0.999378959 |
| 2 | 25101092 | rs2033655 | G | -0.00163 | -0.1535 | 0.530607 | 6.47E-09 | *ADCY3* | 0 |  | 0.977672109 | 0.999391361 |
| 2 | 25103108 | rs2033654 | C | -0.00161 | -0.161 | 0.532046 | 1.15E-09 | *ADCY3* | 0 |  | 0.979067371 | 0.999289952 |
| 2 | 25103446 | rs2033653 | T | -0.00161 | -0.1622 | 0.532161 | 7.41E-10 | *ADCY3* | 0 |  | 0.979231033 | 0.999304397 |
| 2 | 25103967 | rs7591460 | A | -0.00173 | -0.1548 | 0.505091 | 4.82E-09 | *ADCY3* | 0 |  | 0.978728074 | 0.999388 |
| 2 | 25107759 | rs11686663 | C | -0.00162 | -0.1544 | 0.53171 | 5.28E-09 | *ADCY3* | 0 |  | 0.977907678 | 0.999275698 |
| 2 | 25108197 | rs1865689 | T | -0.00167 | -0.1545 | 0.51985 | 5.16E-09 | *ADCY3* | 0 |  | 0.978252109 | 0.999388696 |
| 2 | 25109302 | rs6545790 | A | -0.00165 | -0.1636 | 0.522806 | 5.29E-10 | *ADCY3* | 0 |  | 0.979566451 | 0.999384452 |
| 2 | 25110962 | rs6749170 | A | -0.00182 | -0.1637 | 0.481831 | 5.17E-10 | *ADCY3* | 0 |  | 0.980623615 | 0.999273652 |
| 2 | 25118885 | rs6545800 | C | -0.00143 | -0.1629 | 0.57973 | 6.27E-10 | *ADCY3* | 0 |  | 0.97810526 | 0.999289407 |
| 2 | 25120086 | rs6752483 | C | -0.00156 | -0.1625 | 0.5453 | 8.03E-10 | *ADCY3* | 0 |  | 0.97887378 | 0.999418186 |
| 2 | 25120713 | rs6723803 | G | -0.00148 | -0.1625 | 0.566615 | 8.03E-10 | *ADCY3* | 0 |  | 0.978344079 | 0.999380895 |
| 2 | 25120851 | rs6713978 | T | -0.00145 | -0.1625 | 0.573486 | 8.03E-10 | *ADCY3* | 0 |  | 0.978174893 | 0.999379798 |
| 2 | 25121125 | rs6756609 | C | -0.00155 | -0.1624 | 0.548647 | 8.22E-10 | *ADCY3* | 0 |  | 0.978781576 | 0.999283468 |
| 2 | 25122840 | rs4077678 | C | -0.00146 | -0.1619 | 0.570931 | 9.26E-10 | *ADCY3* | 0 |  | 0.978183453 | 0.999392631 |
| 2 | 25123463 | rs3903070 | C | -0.00135 | -0.1628 | 0.601134 | 7.47E-10 | *ADCY3* | 0 |  | 0.97752758 | 0.999374666 |
| 2 | 25126230 | rs6712981 | A | -0.00136 | -0.1623 | 0.599171 | 7.24E-10 | *ADCY3* | 0 |  | 0.977586197 | 0.999378382 |
| 2 | 25126328 | rs6726199 | G | -0.00126 | -0.1622 | 0.624293 | 8.62E-10 | *ADCY3* | 0 |  | 0.976918278 | 0.999278632 |
| 2 | 25126715 | rs6545809 | C | -0.00123 | -0.1622 | 0.634514 | 7.41E-10 | *ADCY3* | 0 |  | 0.976733312 | 0.999400538 |
| 2 | 25128351 | rs6706316 | C | -0.00136 | -0.1621 | 0.597539 | 8.83E-10 | *ADCY3* | 0 |  | 0.977551822 | 0.999374134 |
| 2 | 25128719 | rs6721750 | A | -0.00136 | -0.162 | 0.598083 | 9.04E-10 | *ADCY3* | 0 |  | 0.977529153 | 0.99935796 |
| 2 | 25128730 | rs6724772 | T | -0.00136 | -0.162 | 0.597719 | 7.78E-10 | *ADCY3* | 0 |  | 0.977595704 | 0.999363599 |
| 2 | 25130451 | rs11689546 | G | -0.00322 | 0.1652 | 0.523807 | 1.38E-09 | *ADCY3* | 0 |  | 0.979190733 | 0.99925906 |
| 2 | 25130462 | rs10200566 | T | -0.00102 | -0.1632 | 0.692405 | 6.79E-10 | *ADCY3* | 0 |  | 0.975419402 | 0.999237809 |
| 2 | 25130542 | rs10198275 | A | -0.00132 | -0.164 | 0.610036 | 5.61E-10 | *ADCY3* | 0 |  | 0.977407322 | 0.999362307 |
| 2 | 25130907 | rs6733224 | T | -0.00136 | -0.1659 | 0.597834 | 4.14E-10 | *ADCY3* | 0 |  | 0.977780507 | 0.999171714 |
| 2 | 25131316 | rs6545814 | A | -0.0015 | -0.1642 | 0.561666 | 5.34E-10 | *ADCY3* | 0 |  | 0.978594441 | 0.999358271 |
| 2 | 25131986 | rs11900505 | A | -0.00133 | -0.1614 | 0.60473 | 8.97E-10 | *ADCY3* | 0 |  | 0.9773713 | 0.999248151 |
| 2 | 25132192 | rs6722587 | C | -0.0015 | -0.1615 | 0.560952 | 1.02E-09 | *ADCY3* | 0 |  | 0.978391605 | 0.999252523 |
| 2 | 25135620 | rs2384061 | G | -0.00129 | -0.1516 | 0.620226 | 9.91E-09 | *ADCY3* | 0 |  | 0.974577 | 0.999355289 |
| 2 | 25136866 | rs10203386 | T | -0.00137 | -0.162 | 0.595168 | 9.04E-10 | *ADCY3* | 0 |  | 0.977599866 | 0.999353598 |
| 2 | 25138040 | rs6737082 | A | 0.000248 | -0.1523 | 0.923296 | 1.25E-08 | *ADCY3* | 0 |  | 0.966611126 | 0.999356593 |
| 2 | 25141538 | rs11676272 | A | -0.00146 | -0.1612 | 0.565022 | 5.77E-09 | *ADCY3* | 0 |  | 0.976914652 | 0.999396845 |
| 2 | 25150116 | rs6752378 | C | -0.00176 | -0.1695 | 0.49033 | 1.05E-10 | *ADCY3* | 8060 |  | 0.980646516 | 0.999282993 |
| 2 | 25150296 | rs10182181 | A | -0.00177 | -0.1682 | 0.48624 | 1.46E-10 | *ADCY3* | 8240 |  | 0.980720132 | 0.999354789 |
| 2 | 25153986 | rs6726261 | T | 0.002796 | -0.1597 | 0.290792 | 4.73E-09 | *ADCY3* | 11930 |  | 0.98510267 | 0.999243923 |
| 2 | 25156773 | rs2384054 | T | -0.00154 | -0.1649 | 0.548192 | 3.30E-10 | *DNAJC27* | 9730 |  | 0.979039207 | 0.999240019 |
| 2 | 25158008 | rs713586 | T | -0.00143 | -0.1647 | 0.578287 | 3.46E-10 | *DNAJC27* | 8495 |  | 0.978292128 | 0.999343137 |
| 2 | 25158281 | rs713587 | C | -0.00133 | -0.1646 | 0.603149 | 3.55E-10 | *DNAJC27* | 8222 |  | 0.977688318 | 0.999352059 |
| 2 | 25169200 | rs1172294 | A | -0.00266 | -0.1698 | 0.296878 | 2.21E-10 | *DNAJC27* | 0 |  | 0.985971156 | 0.999355995 |
| 2 | 25176200 | rs754536 | C | 0.001612 | 0.164 | 0.531166 | 1.36E-09 | *DNAJC27* | 0 |  | 0.979007946 | 0.999338284 |
| 2 | 25176277 | rs754537 | A | 0.001634 | 0.1642 | 0.525621 | 1.30E-09 | *DNAJC27* | 0 |  | 0.979171823 | 0.999241737 |
| 2 | 25187599 | rs4665736 | C | 0.002932 | 0.1694 | 0.254284 | 2.43E-10 | *DNAJC27* | 0 |  | 0.987308112 | 0.999243742 |
| 2 | 25205427 | rs1982200 | C | 0.002658 | 0.1614 | 0.296418 | 8.97E-10 | *LOC729723* | 0 | 5 | 0.985738931 | 0.999352516 |
| 2 | 25239264 | rs11125884 | A | 0.003028 | 0.1648 | 0.239352 | 4.62E-10 | *LOC729723* | 0 |  | 0.987718315 | 0.999353394 |
| 2 | 25239969 | rs12466350 | C | 0.00293 | 0.1608 | 0.254851 | 2.83E-09 | *LOC729723* | 0 |  | 0.986644934 | 0.999355113 |
| 2 | 25286435 | rs1077492 | C | 0.003235 | 0.1648 | 0.209773 | 5.39E-10 | *EFR3B* | 0 | 6 | 0.988706763 | 0.999389463 |
| 2 | 25301755 | rs478222 | A | 0.00334 | 0.1455 | 0.201576 | 8.52E-08 | *EFR3B* | 0 |  | 0.9824503 | 0.999544181 |
| 2 | 25303772 | rs493090 | G | 0.003393 | 0.1408 | 0.194399 | 1.59E-07 | *EFR3B* | 0 |  | 0.979457178 | 0.99942044 |
| 2 | 25304574 | rs567359 | A | 0.00343 | 0.1358 | 0.189418 | 4.28E-07 | *EFR3B* | 0 |  | 0.970688164 | 0.999366883 |
| 2 | 25305136 | rs483428 | C | 0.003364 | 0.1399 | 0.198186 | 1.90E-07 | *EFR3B* | 0 |  | 0.977937355 | 0.999352155 |
| 2 | 25305504 | rs551573 | G | 0.003378 | 0.1344 | 0.19612 | 5.61E-07 | *EFR3B* | 0 |  | 0.966359746 | 0.999358686 |
| 2 | 25305756 | rs1530016 | C | 0.003387 | 0.1395 | 0.195086 | 2.06E-07 | *EFR3B* | 0 |  | 0.977530479 | 0.999339384 |
| 2 | 25316584 | rs522806 | C | 0.00332 | 0.1375 | 0.204211 | 2.76E-07 | *EFR3B* | 0 |  | 0.974326255 | 0.999349917 |
| 2 | 25333735 | rs519111 | T | 0.003056 | 0.1386 | 0.243948 | 2.46E-07 | *EFR3B* | 0 |  | 0.972687739 | 0.999265712 |
| 2 | 25334340 | rs839652 | C | 0.00297 | 0.1411 | 0.257578 | 1.85E-07 | *EFR3B* | 0 |  | 0.97445376 | 0.999343561 |
| 2 | 25404982 | rs4665774 | T | -0.00284 | 0.175 | 0.386739 | 4.51E-07 | *POMC* | 13422 | 7 | 0.954789398 | 0.999369529 |
| 4 | 45175691 | rs13130484 | C | -0.00329 | -0.1434 | 0.202376 | 1.30E-07 | *GNPDA2* | 447078 | 8 | 0.980206591 | 0.999701006 |
| 4 | 45179883 | rs12641981 | C | -0.00335 | -0.142 | 0.193795 | 1.55E-07 | *GNPDA2* | 451270 |  | 0.979653748 | 0.999605504 |
| 4 | 130731284 | rs4864201 | T | 0.005051 | 0.1355 | 0.058066 | 1.41E-06 | *C4orf33* | 697440 | 9 | 0.971817315 | 0.999550786 |
| 12 | 50169070 | rs2720296 | G | -0.00122 | -0.1583 | 0.649719 | 7.50E-08 | *TMBIM6* | 10352 | 10 | 0.96503215 | 0.999366424 |
| 12 | 50171714 | rs2720295 | A | -0.00293 | -0.1526 | 0.284462 | 3.47E-07 | *TMBIM6* | 12996 |  | 0.965981792 | 0.884484003 |
| 12 | 50175380 | rs2720293 | A | -0.00374 | -0.1559 | 0.203192 | 3.70E-07 | *NCKAP5L* | 9547 |  | 0.971277396 | 0.999605065 |
| 12 | 50177324 | rs11836282 | A | -0.0025 | -0.1517 | 0.391075 | 3.38E-07 | *NCKAP5L* | 7603 |  | 0.959408891 | 0.999484594 |
| 12 | 50183874 | rs2603107 | T | -0.00263 | -0.1386 | 0.320582 | 2.46E-07 | *NCKAP5L* | 1053 |  | 0.967902829 | 0.999524609 |
| 12 | 50183966 | rs1470909 | T | -0.0026 | -0.1396 | 0.32633 | 2.02E-07 | *NCKAP5L* | 961 |  | 0.969729683 | 0.999521161 |
| 12 | 50188434 | rs2603105 | T | -0.00277 | -0.1435 | 0.317034 | 4.80E-07 | *NCKAP5L* | 0 |  | 0.958654985 | 0.999450092 |
| 12 | 50192009 | rs1075366 | T | -0.00279 | -0.1377 | 0.290471 | 2.95E-07 | *NCKAP5L* | 0 |  | 0.967667719 | 0.999469542 |
| 12 | 50199949 | rs2603112 | A | -0.00385 | -0.1475 | 0.153378 | 7.10E-08 | *NCKAP5L* | 0 |  | 0.985785316 | 0.999647746 |
| 12 | 50204337 | rs7313563 | C | -0.00385 | -0.1525 | 0.150437 | 7.09E-08 | *NCKAP5L* | 0 |  | 0.985956854 | 0.999650509 |
| 12 | 50204709 | rs4898530 | A | -0.00327 | -0.1432 | 0.21199 | 9.73E-08 | *NCKAP5L* | 0 |  | 0.981265913 | 0.999598199 |
| 12 | 50206723 | rs10875969 | G | -0.00294 | -0.1405 | 0.261575 | 1.35E-07 | *NCKAP5L* | 0 |  | 0.976650607 | 0.999562348 |
| 12 | 50207126 | rs11169162 | T | -0.00339 | -0.146 | 0.204517 | 9.62E-08 | *NCKAP5L* | 0 |  | 0.981723761 | 0.99960402 |
| 12 | 50208343 | rs11169163 | A | -0.00244 | -0.146 | 0.365774 | 1.33E-07 | *NCKAP5L* | 0 |  | 0.971524369 | 0.999416761 |
| 12 | 50212628 | rs2336448 | C | -0.00308 | -0.1408 | 0.240039 | 1.59E-07 | *NCKAP5L* | 0 |  | 0.976711884 | 0.999577374 |
| 12 | 50214637 | rs4075681 | C | -0.00259 | -0.1435 | 0.347526 | 3.59E-07 | *NCKAP5L* | 0 |  | 0.96122369 | 0.999508751 |
| 12 | 50241013 | rs11169176 | G | -0.00422 | -0.1273 | 0.100008 | 2.14E-06 | *BCDIN3D* | 4100 |  | 0.95402301 | 0.999703977 |
| 12 | 50244767 | rs10875980 | A | -0.00416 | -0.1268 | 0.104762 | 1.79E-06 | *BCDIN3D* | 7854 |  | 0.957297724 | 0.999698333 |
| 12 | 50245706 | rs10875982 | A | -0.00407 | -0.1275 | 0.112664 | 1.57E-06 | *BCDIN3D* | 8793 |  | 0.958695091 | 0.99964077 |
| 12 | 50247468 | rs7138803 | G | -0.00317 | -0.1672 | 0.229275 | 6.50E-10 | *BCDIN3D* | 10555 |  | 0.98800015 | 0.999403757 |
| 12 | 50263148 | rs7132908 | G | -0.00432 | -0.1635 | 0.099439 | 1.24E-08 | *FAIM2* | 0 |  | 0.967831783 | 0.999704663 |
| 12 | 50285061 | rs297924 | T | -0.00452 | -0.1531 | 0.087538 | 1.20E-08 | *FAIM2* | 0 |  | 0.992562288 | 0.999719584 |
| 12 | 50285562 | rs17201502 | C | -0.00479 | -0.165 | 0.073371 | 2.51E-09 | *FAIM2* | 0 |  | 0.99405914 | 0.999739012 |
| 16 | 53798523 | rs8047395 | G | -0.00341 | -0.1634 | 0.180955 | 4.08E-09 | *FTO* | 0 | 11 | 0.989114178 | 0.999563416 |
| 16 | 53799507 | rs9937053 | G | -0.00549 | -0.1776 | 0.032649 | 2.22E-10 | *FTO* | 0 |  | 0.996690366 | 0.999812242 |
| 16 | 53799905 | rs9928094 | A | -0.00546 | -0.1942 | 0.033576 | 7.20E-13 | *FTO* | 0 |  | 0.996683891 | 0.999781745 |
| 16 | 53799977 | rs9930333 | T | -0.00548 | -0.1776 | 0.033083 | 1.64E-10 | *FTO* | 0 |  | 0.996671883 | 0.999811231 |
| 16 | 53800387 | rs12446228 | A | -0.004 | -0.1368 | 0.127617 | 1.02E-06 | *FTO* | 0 |  | 0.964653111 | 0.999673125 |
| 16 | 53800568 | rs9939973 | G | -0.0056 | -0.1939 | 0.029378 | 6.39E-13 | *FTO* | 0 |  | 0.996963535 | 0.999820133 |
| 16 | 53800629 | rs9940646 | C | -0.00448 | -0.1978 | 0.08126 | 4.06E-13 | *FTO* | 0 |  | 0.994065847 | 0.999685975 |
| 16 | 53800754 | rs9940128 | G | -0.00547 | -0.1942 | 0.033358 | 5.89E-13 | *FTO* | 0 |  | 0.996698255 | 0.99978233 |
| 16 | 53800954 | rs1421085 | T | -0.00579 | -0.191 | 0.02557 | 1.40E-12 | *FTO* | 0 |  | 0.98700937 | 0.999804896 |
| 16 | 53801549 | rs9923147 | C | -0.00555 | -0.1941 | 0.030848 | 6.06E-13 | *FTO* | 0 |  | 0.996864217 | 0.999816526 |
| 16 | 53801985 | rs9923544 | C | -0.00556 | -0.1942 | 0.030571 | 5.89E-13 | *FTO* | 0 |  | 0.996882832 | 0.999817198 |
| 16 | 53803156 | rs8055197 | G | -0.00408 | -0.1568 | 0.108484 | 4.03E-09 | *FTO* | 0 |  | 0.992176815 | 0.99969402 |
| 16 | 53803574 | rs1558902 | T | -0.00391 | -0.1905 | 0.131348 | 1.32E-12 | *FTO* | 0 |  | 0.991863139 | 0.999669272 |
| 16 | 53804340 | rs1861866 | C | -0.0041 | -0.1568 | 0.106692 | 4.03E-09 | *FTO* | 0 |  | 0.992260197 | 0.999696085 |
| 16 | 53804965 | rs10852521 | T | -0.00394 | -0.1552 | 0.1217 | 5.78E-09 | *FTO* | 0 |  | 0.991389212 | 0.999679375 |
| 16 | 53805207 | rs11075985 | C | -0.00547 | -0.1957 | 0.033465 | 3.91E-13 | *FTO* | 0 |  | 0.996691533 | 0.999810347 |
| 16 | 53806145 | rs2058908 | T | -0.0057 | -0.1745 | 0.048647 | 3.39E-06 | *FTO* | 0 |  | 0.957536654 | 0.999779181 |
| 16 | 53806280 | rs9922047 | C | -0.00331 | -0.1392 | 0.193526 | 1.58E-07 | *FTO* | 0 |  | 0.979536811 | 0.999612816 |
| 16 | 53807764 | rs17817288 | A | -0.00395 | -0.1543 | 0.120851 | 7.07E-09 | *FTO* | 0 |  | 0.991302702 | 0.999680287 |
| 16 | 53808258 | rs1477196 | A | -0.00401 | -0.1365 | 0.126411 | 8.98E-07 | *FTO* | 0 |  | 0.967025008 | 0.999674385 |
| 16 | 53809247 | rs1121980 | G | -0.00562 | -0.1961 | 0.028623 | 2.85E-13 | *FTO* | 0 |  | 0.997015671 | 0.999822028 |
| 16 | 53810686 | rs7193144 | T | -0.00602 | -0.1926 | 0.020638 | 4.96E-13 | *FTO* | 0 |  | 0.997594649 | 0.999844197 |
| 16 | 53812614 | rs8057044 | G | -0.00472 | -0.1637 | 0.064114 | 5.17E-10 | *FTO* | 0 |  | 0.994794655 | 0.999752941 |
| 16 | 53813367 | rs17817449 | T | -0.0059 | -0.1927 | 0.023222 | 3.93E-13 | *FTO* | 0 |  | 0.997400068 | 0.999836539 |
| 16 | 53813450 | rs8043757 | A | -0.00592 | -0.1928 | 0.022855 | 3.82E-13 | *FTO* | 0 |  | 0.997427265 | 0.999837595 |
| 16 | 53815161 | rs11075987 | T | -0.00398 | -0.1616 | 0.117395 | 6.31E-10 | *FTO* | 0 |  | 0.992247887 | 0.999684037 |
| 16 | 53816275 | rs8050136 | C | -0.006 | -0.1909 | 0.021102 | 5.27E-13 | *FTO* | 0 |  | 0.997559067 | 0.999842782 |
| 16 | 53816647 | rs4783819 | G | -0.00404 | -0.1397 | 0.123279 | 4.07E-07 | *FTO* | 0 |  | 0.97743397 | 0.99967769 |
| 16 | 53816752 | rs8051591 | A | -0.00604 | -0.1932 | 0.020257 | 2.77E-13 | *FTO* | 0 |  | 0.997624302 | 0.999822734 |
| 16 | 53816838 | rs9935401 | G | -0.00612 | -0.1935 | 0.018607 | 2.54E-13 | *FTO* | 0 |  | 0.997753876 | 0.999850626 |
| 16 | 53818460 | rs3751812 | G | -0.00624 | -0.1932 | 0.016508 | 2.77E-13 | *FTO* | 0 |  | 0.990243298 | 0.999857724 |
| 16 | 53818708 | rs3751813 | G | -0.00519 | -0.166 | 0.042114 | 6.39E-10 | *FTO* | 0 |  | 0.99602729 | 0.99979176 |
| 16 | 53819169 | rs9936385 | T | -0.00615 | -0.184 | 0.018093 | 5.77E-11 | *FTO* | 0 |  | 0.997777239 | 0.99983079 |
| 16 | 53819198 | rs9923233 | G | -0.00611 | -0.1838 | 0.01878 | 2.24E-11 | *FTO* | 0 |  | 0.997730761 | 0.999850062 |
| 16 | 53819877 | rs11075989 | C | -0.0061 | -0.1781 | 0.019123 | 7.70E-11 | *FTO* | 0 |  | 0.997690908 | 0.999848954 |
| 16 | 53819893 | rs11075990 | A | -0.00609 | -0.1934 | 0.019217 | 2.62E-13 | *FTO* | 0 |  | 0.997705534 | 0.999848653 |
| 16 | 53820527 | rs9939609 | T | -0.00607 | -0.1934 | 0.019682 | 2.62E-13 | *FTO* | 0 |  | 0.997669042 | 0.999847174 |
| 16 | 53821615 | rs7202116 | A | -0.00607 | -0.1892 | 0.019704 | 1.39E-11 | *FTO* | 0 |  | 0.989039838 | 0.999847104 |
| 16 | 53821862 | rs7201850 | C | -0.00588 | -0.1831 | 0.022585 | 2.66E-11 | *FTO* | 0 |  | 0.99743628 | 0.999838378 |
| 16 | 53822651 | rs7185735 | A | -0.00601 | -0.1927 | 0.021 | 3.93E-13 | *FTO* | 0 |  | 0.997567008 | 0.999843091 |
| 16 | 53825488 | rs9941349 | C | -0.0061 | -0.1978 | 0.01811 | 1.16E-13 | *FTO* | 0 |  | 0.997793857 | 0.999852261 |
| 16 | 53827179 | rs9931494 | C | -0.0061 | -0.1974 | 0.018184 | 1.30E-13 | *FTO* | 0 |  | 0.997787888 | 0.99983044 |
| 16 | 53828066 | rs17817964 | C | -0.00628 | -0.1933 | 0.01591 | 4.09E-13 | *FTO* | 0 |  | 0.997974269 | 0.999859843 |
| 16 | 53828752 | rs7190492 | A | -0.00398 | -0.1359 | 0.128617 | 6.88E-07 | *FTO* | 0 |  | 0.970738242 | 0.999543581 |
| 16 | 53830452 | rs9930501 | A | -0.00477 | -0.1881 | 0.063009 | 1.70E-12 | *FTO* | 0 |  | 0.994978579 | 0.999754682 |
| 16 | 53830465 | rs9930506 | A | -0.00451 | -0.1874 | 0.078935 | 2.05E-12 | *FTO* | 0 |  | 0.994175707 | 0.999731137 |
| 16 | 53830491 | rs9932754 | T | -0.00478 | -0.1868 | 0.062388 | 1.98E-12 | *FTO* | 0 |  | 0.995010898 | 0.999755668 |
| 16 | 53831146 | rs9922708 | C | -0.00455 | -0.1866 | 0.076468 | 2.09E-12 | *FTO* | 0 |  | 0.994296019 | 0.999734586 |
| 16 | 53831771 | rs9922619 | G | -0.00458 | -0.1866 | 0.074137 | 2.09E-12 | *FTO* | 0 |  | 0.994410985 | 0.999697612 |
| 16 | 53839135 | rs8044769 | T | -0.00291 | -0.1482 | 0.24657 | 1.25E-08 | *FTO* | 0 |  | 0.985595125 | 0.999572736 |
| 16 | 53842908 | rs12149832 | G | -0.00474 | -0.1837 | 0.067201 | 5.49E-12 | *FTO* | 0 |  | 0.994757697 | 0.999748171 |
| 16 | 53845487 | rs11642841 | C | -0.00636 | -0.1882 | 0.015183 | 2.48E-11 | *FTO* | 0 |  | 0.998026647 | 0.999842574 |
| 16 | 53848561 | rs1861867 | A | -0.00318 | -0.1742 | 0.246332 | 5.93E-07 | *FTO* | 0 |  | 0.960794538 | 0.999572903 |
| 18 | 38765542 | rs17697435 | T | -0.01047 | -0.1859 | 0.006913 | 1.87E-06 | *KC6* | 294692 | 12 | 0.984847118 | 0.999802784 |
| 18 | 38765623 | rs17697453 | A | -0.01047 | -0.1854 | 0.006905 | 2.00E-06 | *KC6* | 294611 |  | 0.98425585 | 0.99969545 |
| 18 | 38765659 | rs17697518 | C | -0.01069 | -0.1855 | 0.005827 | 1.85E-06 | *KC6* | 294575 |  | 0.985572449 | 0.999610725 |
| 18 | 57732689 | rs4940927 | A | -0.00015 | 0.1678 | 0.959149 | 1.68E-08 | *PMAIP1* | 161150 | 13 | 0.964823454 | 0.823631429 |
| 18 | 57744189 | rs7240566 | A | 0.001716 | -0.1577 | 0.560908 | 2.05E-07 | *PMAIP1* | 172650 |  | 0.957871574 | 0.999403195 |
| 18 | 57744576 | rs11520442 | T | 0.00138 | -0.1579 | 0.639825 | 1.98E-07 | *PMAIP1* | 173037 |  | 0.954907659 | 0.999265875 |
| 18 | 57751185 | rs4299252 | A | 0.001579 | -0.1558 | 0.592479 | 2.86E-07 | *PMAIP1* | 179646 |  | 0.951166385 | 0.99938976 |
| 18 | 57751960 | rs8091524 | T | 0.001527 | -0.1558 | 0.605084 | 2.86E-07 | *PMAIP1* | 180421 |  | 0.950549318 | 0.999384514 |
| 18 | 57755117 | rs6567155 | C | 0.001466 | -0.1563 | 0.619348 | 2.39E-07 | *PMAIP1* | 183578 |  | 0.952931497 | 0.999378656 |
| 18 | 57766512 | rs1539952 | A | 0.001642 | -0.1562 | 0.579182 | 2.67E-07 | *PMAIP1* | 194973 |  | 0.95301274 | 0.999395365 |
| 18 | 57775295 | rs9966951 | G | 0.003152 | -0.1424 | 0.248255 | 3.63E-07 | *PMAIP1* | 203756 |  | 0.968008096 | 0.999496184 |
| 18 | 57777991 | rs9951795 | C | 0.002375 | -0.1452 | 0.382768 | 2.13E-07 | *PMAIP1* | 206452 |  | 0.966079291 | 0.999487735 |
| 18 | 57781188 | rs948760 | A | 0.002363 | -0.1459 | 0.384687 | 1.86E-07 | *PMAIP1* | 209649 |  | 0.967459758 | 0.999403351 |
| 18 | 57787559 | rs1893512 | T | 0.002627 | -0.1499 | 0.334034 | 7.65E-08 | *PMAIP1* | 216020 |  | 0.976720071 | 0.999435465 |
| 18 | 57790052 | rs756190 | A | 0.003464 | -0.147 | 0.202854 | 1.36E-07 | *PMAIP1* | 218513 |  | 0.979943084 | 0.999541012 |
| 18 | 57790225 | rs6567157 | T | 0.002558 | -0.1465 | 0.346799 | 1.49E-07 | *PMAIP1* | 218686 |  | 0.971479428 | 0.999507766 |
| 18 | 57791081 | rs8087080 | G | 0.002584 | -0.1466 | 0.342011 | 1.47E-07 | *PMAIP1* | 219542 |  | 0.97188545 | 0.999510064 |
| 18 | 57793209 | rs1942880 | C | 0.002903 | -0.1458 | 0.286117 | 1.90E-07 | *PMAIP1* | 221670 |  | 0.972579734 | 0.999469138 |
| 18 | 57793589 | rs8084834 | C | 0.002904 | -0.1464 | 0.285816 | 1.69E-07 | *PMAIP1* | 222050 |  | 0.973609862 | 0.999471539 |
| 18 | 57797485 | rs953442 | T | 0.00224 | -0.161 | 0.450384 | 1.25E-07 | *PMAIP1* | 225946 |  | 0.968243264 | 0.999454138 |
| 18 | 57798110 | rs952044 | C | 0.002923 | -0.1464 | 0.282997 | 1.88E-07 | *PMAIP1* | 226571 |  | 0.972868559 | 0.999546233 |
| 18 | 57799449 | rs1942859 | T | 0.00332 | -0.1472 | 0.223071 | 1.78E-07 | *PMAIP1* | 227910 |  | 0.976839711 | 0.99958819 |
| 18 | 57804346 | rs8095404 | A | 0.005775 | -0.1255 | 0.057084 | 3.24E-06 | *PMAIP1* | 232807 |  | 0.955547236 | 0.994868903 |
| 18 | 57811982 | rs17700144 | G | 0.001621 | -0.1825 | 0.605279 | 1.46E-08 | *MC4R* | 226580 |  | 0.974089265 | 0.999384433 |
| 18 | 57829135 | rs6567160 | T | 0.001146 | -0.1974 | 0.703783 | 1.85E-10 | *MC4R* | 209427 |  | 0.97547787 | 0.99934552 |
| 18 | 57838401 | rs663129 | G | 0.001428 | -0.1992 | 0.635183 | 1.27E-10 | *MC4R* | 200161 |  | 0.977097172 | 0.999372247 |
| 18 | 57839769 | rs571312 | C | 0.001423 | -0.1986 | 0.636183 | 1.25E-10 | *MC4R* | 198793 |  | 0.977075404 | 0.999371845 |
| 18 | 57848369 | rs523288 | A | 0.00268 | -0.1974 | 0.372723 | 1.85E-10 | *MC4R* | 190193 |  | 0.983752749 | 0.999412227 |
| 18 | 57848531 | rs2168711 | T | 0.001335 | -0.1975 | 0.657837 | 1.82E-10 | *MC4R* | 190031 |  | 0.976526187 | 0.999363239 |
| 18 | 57849023 | rs12967135 | G | 0.001707 | -0.195 | 0.570728 | 3.06E-10 | *MC4R* | 189539 |  | 0.978500381 | 0.999398969 |
| 18 | 57850422 | rs538656 | G | 0.002068 | -0.1963 | 0.492361 | 2.68E-10 | *MC4R* | 188140 |  | 0.98048294 | 0.999433999 |
| 18 | 57851097 | rs17782313 | T | 0.001622 | -0.1962 | 0.589727 | 2.73E-10 | *MC4R* | 187465 |  | 0.978061937 | 0.999390914 |
| 18 | 57851763 | rs10871777 | A | 0.001498 | -0.1946 | 0.617163 | 3.33E-10 | *MC4R* | 186799 |  | 0.977368676 | 0.999379549 |
| 18 | 57852587 | rs476828 | T | 0.001676 | -0.1947 | 0.57546 | 3.26E-10 | *MC4R* | 185975 |  | 0.97837304 | 0.999396948 |
| 18 | 57852948 | rs11152213 | A | 0.001604 | -0.1956 | 0.592453 | 3.10E-10 | *MC4R* | 185614 |  | 0.977972938 | 0.999389771 |
| 18 | 57858802 | rs492443 | A | 0.000984 | -0.1749 | 0.733095 | 6.60E-09 | *MC4R* | 179760 |  | 0.972508986 | 0.998979715 |
| 18 | 57858829 | rs8089364 | T | 0.000828 | -0.1722 | 0.774727 | 1.41E-08 | *MC4R* | 179733 |  | 0.969802382 | 0.999205757 |
| 18 | 57859563 | rs12969709 | C | 0.000948 | -0.1713 | 0.74309 | 1.34E-08 | *MC4R* | 178999 |  | 0.970736 | 0.999330898 |
| 18 | 57861663 | rs921971 | T | 0.000828 | -0.1709 | 0.77464 | 1.44E-08 | *MC4R* | 176899 |  | 0.969731043 | 0.999319492 |
| 18 | 57861961 | rs1457489 | G | 0.00079 | -0.1704 | 0.784613 | 1.42E-08 | *MC4R* | 176601 |  | 0.96953179 | 0.999315944 |
| 18 | 57876034 | rs11665563 | C | 0.001055 | -0.1667 | 0.71467 | 2.60E-08 | *MC4R* | 162528 |  | 0.969233305 | 0.999341422 |
| 18 | 57876227 | rs11663816 | T | 0.000984 | -0.1582 | 0.733319 | 1.27E-07 | *MC4R* | 162335 |  | 0.957051922 | 0.999334488 |
| 18 | 57877800 | rs11664883 | T | 0.001026 | -0.1666 | 0.722154 | 2.65E-08 | *MC4R* | 160762 |  | 0.96895095 | 0.999338627 |
| 18 | 57884750 | rs12970134 | G | 0.000895 | -0.1665 | 0.756368 | 3.01E-08 | *MC4R* | 153812 |  | 0.967454981 | 0.999326063 |
| 18 | 57893618 | rs8083289 | C | 0.000762 | -0.1655 | 0.792289 | 4.04E-08 | *MC4R* | 144944 |  | 0.964917955 | 0.99931323 |
| 18 | 57896742 | rs17175643 | C | 0.001114 | -0.165 | 0.699944 | 4.44E-08 | *MC4R* | 141820 |  | 0.96700963 | 0.999346973 |
| 18 | 57897803 | rs12960928 | T | 0.00118 | -0.1649 | 0.683764 | 4.52E-08 | *MC4R* | 140759 |  | 0.967380669 | 0.999353152 |
| 18 | 57903604 | rs12964203 | T | 0.001079 | -0.1639 | 0.709689 | 5.45E-08 | *MC4R* | 134958 |  | 0.965439325 | 0.999343292 |
| 18 | 57904088 | rs590215 | C | 0.001262 | -0.1636 | 0.662705 | 5.19E-08 | *MC4R* | 134474 |  | 0.967189885 | 0.999361327 |
| 18 | 57907311 | rs2168708 | G | 0.00098 | -0.1644 | 0.735339 | 6.11E-08 | *MC4R* | 131251 |  | 0.963859194 | 0.999333744 |
| 18 | 57911330 | rs12966550 | A | 0.001005 | -0.1653 | 0.729215 | 5.74E-08 | *MC4R* | 127232 |  | 0.96449743 | 0.999336006 |

**Table S7: IPA Canonical Pathways with at least 2 molecules and P-value significance < 0.05**.

| **Ingenuity Canonical Pathways** | **P-values** | **Ratio** | **Molecules** |
| --- | --- | --- | --- |
| RAR Activation | 6.18E-03 | 0.0103 | ADCY3, NCOA1 |
| Estrogen Receptor Signaling | 1.69E-02 | 0.0061 | ADCY3, NCOA1 |

**Table S8: IPA pathways for diseases and biological function with at least 2 molecules and P-value significance < 0.05.**

| **Categories** | **Diseases or Functions Annotation** | **P-values** | **Molecules** |
| --- | --- | --- | --- |
| Nervous System Development and Function, Organ Morphology, Organismal Development | Morphology of brain | 8.68E-05 | ADCY3,EBF1,FAIM2,FTO,NCOA1 |
| Nervous System Development and Function, Organ Morphology, Organismal Development | Morphology of rhombencephalon | 3.26E-04 | EBF1,FAIM2,NCOA1 |
| Nervous System Development and Function, Organ Morphology, Organismal Development | Size of brain | 3.48E-04 | EBF1,FAIM2,FTO |
| Endocrine System Development and Function, Molecular Transport, Small Molecule Biochemistry | Abnormal quantity of hormone | 4.96E-04 | FTO,NCOA1 |

**Table S9: Colocalization analysis of SNPs found to be jointly associated with European birthweight and childhood obesity and its related trait childhood body mass index**

|  | **European Birthweight (BW) & Childhood Body Mass Index (CBMI)** | | | | | | | | | | |
| --- | --- | --- | --- | --- | --- | --- | --- | --- | --- | --- | --- |
| **Genetic loci** | **Nearest Gene** | **Lead SNP** | **CHR** | **Start (500Kb downstream lead SNP)** | **End (500Kb upstream lead SNP)** | **Number of SNPs** | **Posterior probability neither trait has a genetic association in the region(H0)** | **Posterior probability only CBMI/COB has a genetic association in the region(H1)** | **Posterior Probability only BW has a genetic association in the region(H2)** | **Posterior Probability both traits are associated, but with different causal variants(H3)** | **Posterior Probability both traits are associated and share a single causal variant(H4)** |
| 1 | *FPGT-TNNI3K* | rs10789396 | 1 | 74514362 | 75514362 | 657 | 0 | 0.501 | 0 | 0.036 | 0.463 |
| 2 | *NCOA1* | rs10495749 | 2 | 24230847 | 25230847 | 670 | 0 | 0.495 | 0 | 0.485 | 0.02 |
| 3 | *ADCY3* | rs1344840 | 2 | 24570645 | 25570645 | 584 | 0 | 0.852 | 0 | 0.112 | 0.036 |
| 4 | *LMBR1L* | rs7958572 | 12 | 49009441 | 50009441 | 0 | NA | NA | NA | NA | NA |
| 5 | *HMGA2* | rs2358954 | 12 | 65879504 | 66879504 | 0 | NA | NA | NA | NA | NA |
|  | **European Birthweight (BW) & Childhood Obesity (COB)** | | | | | | | | | | |
| 1 | *FPGT-TNNI3K* | rs10493544 | 1 | 74483835 | 75483835 | 789 | 0.005 | 0.512 | 0 | 0.038 | 0.445 |
| 2 | *SEC16B* | rs693232 | 1 | 177381651 | 178381651 | 861 | 0.056 | 0.803 | 0.006 | 0.081 | 0.054 |
| 3 | *TMEM18* | rs2867113 | 2 | 151365 | 1151365 | 1002 | 0 | 0.891 | 0 | 0.072 | 0.037 |
| 4 | *NCOA1* | rs11693308 | 2 | 24446188 | 25446188 | 723 | 0 | 0.618 | 0 | 0.356 | 0.026 |
| 5 | *LOC729723* | rs11125884 | 2 | 24739264 | 25739264 | 632 | 0 | 0.866 | 0 | 0.095 | 0.038 |
| 6 | *EFR3B* | rs1077492 | 2 | 24786435 | 25786435 | 628 | 0 | 0.869 | 0 | 0.092 | 0.039 |
| 7 | *POMC* | rs4665774 | 2 | 24904982 | 25904982 | 602 | 0 | 0.893 | 0 | 0.067 | 0.04 |
| 8 | *GNPDA2* | rs13130484 | 4 | 44675691 | 45675691 | 871 | 0.095 | 0.786 | 0.008 | 0.064 | 0.047 |
| 9 | *C4orf33* | rs4864201 | 4 | 130231284 | 131231284 | 934 | 0.294 | 0.591 | 0.017 | 0.034 | 0.063 |
| 10 | *FAIM2* | rs17201502 | 12 | 49785562 | 50785562 | 453 | 0.019 | 0.825 | 0.002 | 0.074 | 0.081 |
| 11 | *FTO* | rs11642841 | 16 | 53345487 | 54345487 | 924 | 0 | 0.266 | 0 | 0.651 | 0.083 |
| 12 | *KC6* | rs17697518 | 18 | 38265659 | 39265659 | 1041 | 0.413 | 0.328 | 0.036 | 0.029 | 0.194 |
| 13 | *MC4R* | rs523288 | 18 | 57348369 | 58348369 | 1080 | 0 | 0.903 | 0 | 0.064 | 0.033 |

**Table S10: Colocalization analysis of SNPs found to be jointly associated with Transethnic birthweight and childhood obesity and its related trait childhood body mass index**

|  | **Transethnic Birthweight (BW) & Childhood Body Mass Index (CBMI)** | | | | | | | | | | |
| --- | --- | --- | --- | --- | --- | --- | --- | --- | --- | --- | --- |
| **Genetic loci** | **Nearest Gene** | **Lead SNP** | **CHR** | **Start (500Kb downstream lead SNP)** | **End (500Kb upstream lead SNP)** | **Number of SNPs** | **Posterior probability neither trait has a genetic association in the region(H0)** | **Posterior probability only CBMI/COB has a genetic association in the region(H1)** | **Posterior Probability only BW has a genetic association in the region(H2)** | **Posterior Probability both traits are associated, but with different causal variants(H3)** | **Posterior Probability both traits are associated and share a single causal variant(H4)** |
| 1 | *FPGT-TNNI3K* | rs10789396 | 1 | 74514362 | 75514362 | 657 | 0 | 0.6182 | 0 | 0.0381 | 0.3437 |
| 2 | *NCOA1* | rs10495749 | 2 | 24230847 | 25230847 | 670 | 0 | 0.5609 | 0 | 0.4172 | 0.0219 |
| 3 | *ADCY3* | rs1344840 | 2 | 24570645 | 25570645 | 584 | 0 | 0.7973 | 0 | 0.1697 | 0.033 |
| 4 | *EBF1* | rs6887211 | 5 | 157946223 | 158946223 | 889 | 0.001 | 0.0004 | 0.0913 | 0.0356 | 0.8717 |
| 5 | *LMBR1L* | rs7958572 | 12 | 49009441 | 50009441 | 0 | NA | NA | NA | NA | NA |
| 6 | *HMGA2* | rs2358954 | 12 | 65879504 | 66879504 | 0 | NA | NA | NA | NA | NA |
|  | **Transethnic Birthweight (BW) & Childhood Obesity (COB)** | | | | | | | | | | |
| 1 | *FPGT-TNNI3K* | rs6690871 | 1 | 74477277 | 75477277 | 791 | 0.0055 | 0.621 | 0.0004 | 0.0406 | 0.3326 |
| 2 | *NCOA1* | rs11693308 | 2 | 24446188 | 25446188 | 723 | 0.0001 | 0.6504 | 0 | 0.3223 | 0.0271 |
| 3 | *DNAJC27* | rs1172294 | 2 | 24669200 | 25669200 | 665 | 0.0001 | 0.78 | 0 | 0.1862 | 0.0337 |
| 4 | *EFR3B* | rs1077492 | 2 | 24786435 | 25786435 | 628 | 0.0002 | 0.8348 | 0 | 0.1281 | 0.0368 |
| 5 | *C4orf33* | rs4864201 | 4 | 130231284 | 131231284 | 934 | 0.281 | 0.5742 | 0.0184 | 0.0376 | 0.0887 |
| 6 | *NCKAP5L* | rs2720293 | 12 | 49675380 | 50675380 | 470 | 0.0168 | 0.8316 | 0.0012 | 0.0608 | 0.0895 |
| 7 | *BCDIN3D* | rs7138803 | 12 | 49747468 | 50747468 | 461 | 0.018 | 0.8311 | 0.0013 | 0.0598 | 0.0898 |
| 8 | *FTO* | rs3751812 | 16 | 53318460 | 54318460 | 930 | 0 | 0.269 | 0 | 0.6582 | 0.0728 |
| 9 | *KC6* | rs17697518 | 18 | 38265659 | 39265659 | 1041 | 0.4313 | 0.3299 | 0.0399 | 0.0303 | 0.1687 |

**
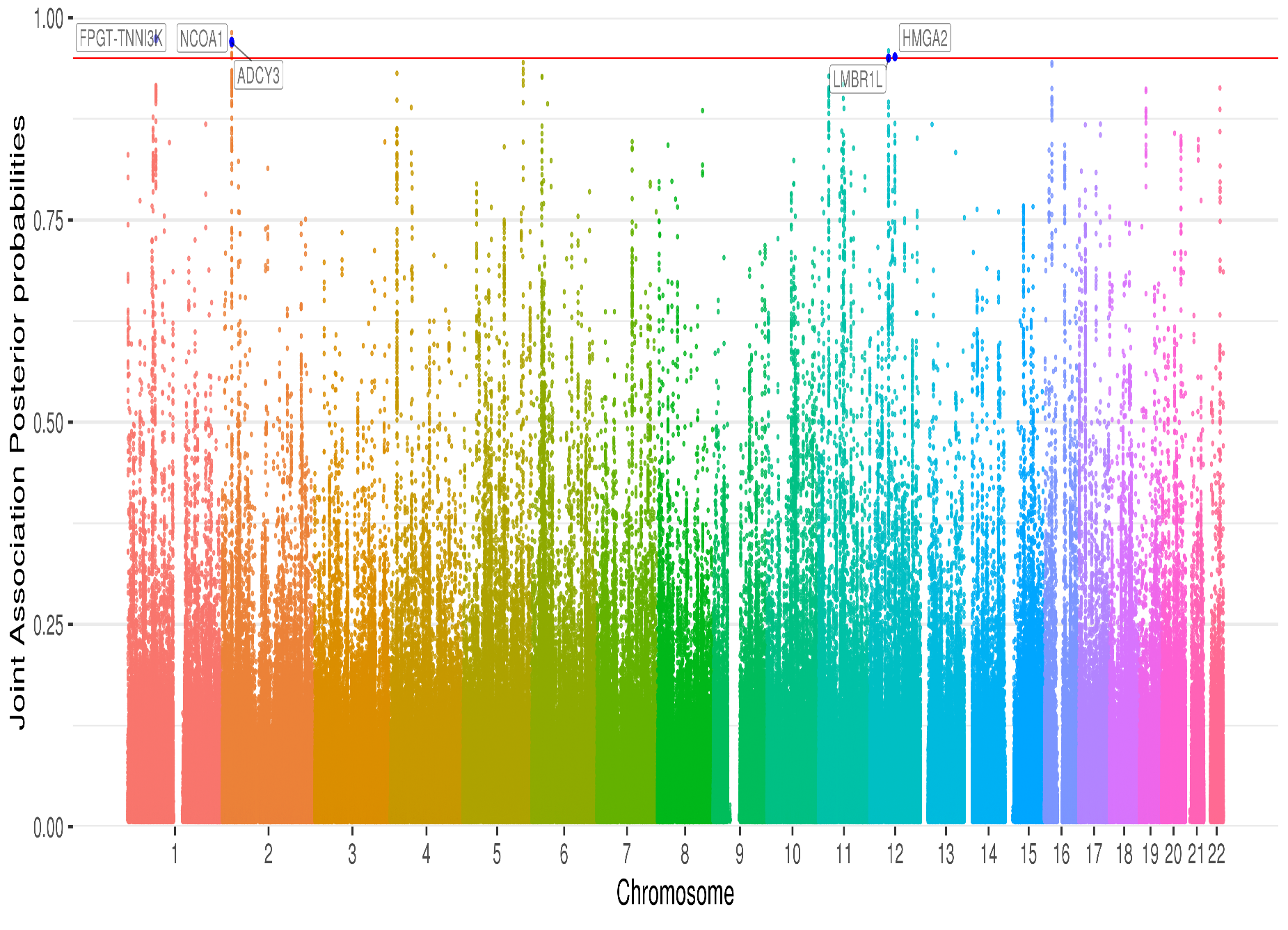
**

**Figure S1: Manhattan plot of joint association of variants with childhood body mass index and European birth weight.** The red line denotes the threshold of posterior probability (PP) at 0.95. The colored points under the red line are all common non-significant SNPs between childhood body mass index and European birth weight that have PP < 0.95. The blue circles above or on the red line are all common significant SNPs found between childhood body mass index and European birth weight that have PP ≥ 0.95. The text boxes contain the genes associated to the 5 pleiotropic genetic loci that was found jointly associated with both childhood body mass index and European birth weight.

**
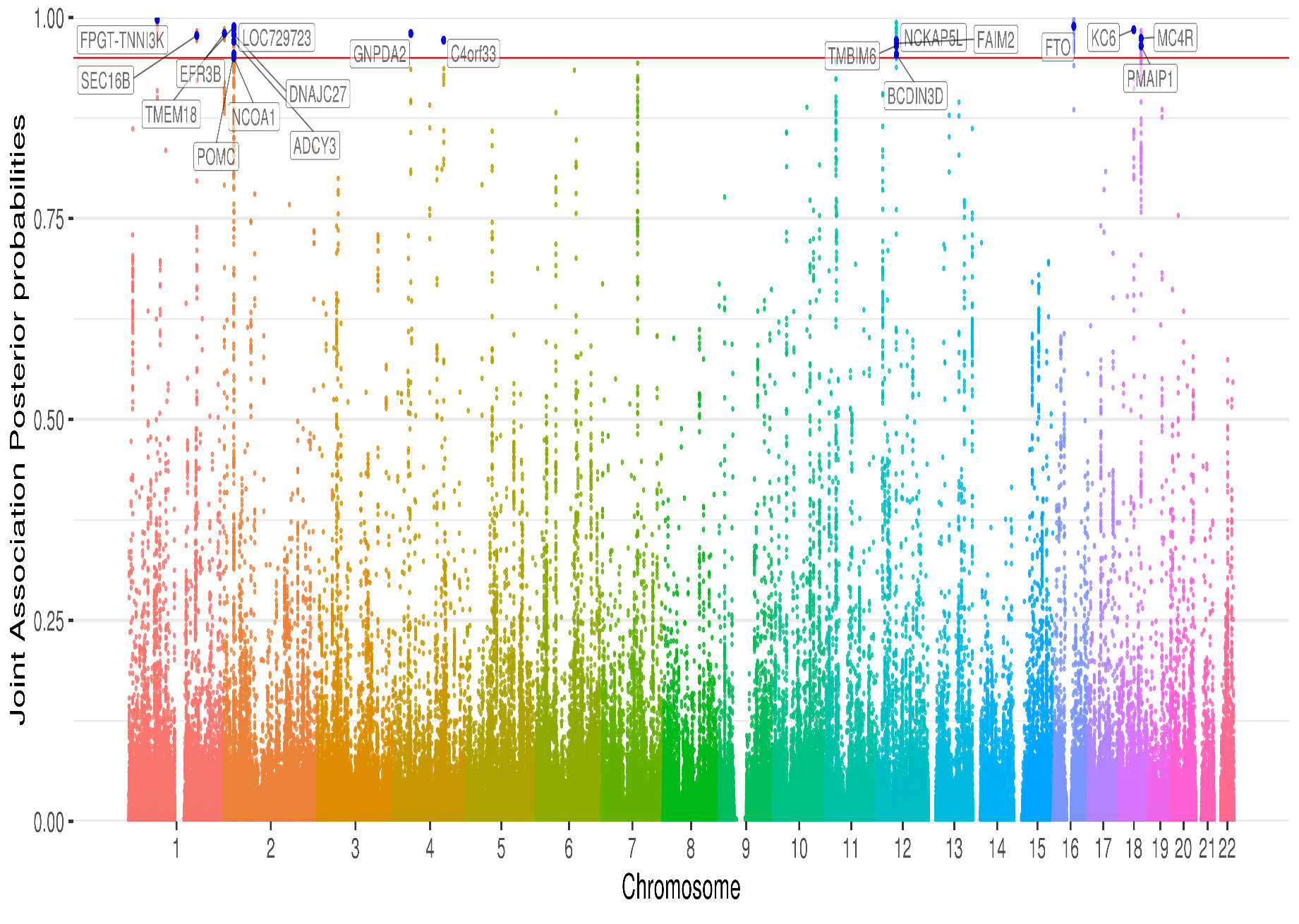
**

**Figure S2: Manhattan plot of joint association of variants with childhood Obesity and European birth weight.** The red line denotes the threshold of posterior probability (PP) at 0.95. The colored points under the red line are all common non-significant SNPs between childhood Obesity and European birth weight that have PP < 0.95. The blue circles above or on the red line are all common significant SNPs found between childhood Obesity and European birth weight that have PP ≥ 0.95. The text boxes contain the genes associated with the 13 pleiotropic genetic loci that was found jointly associated with both childhood Obesity and European birth weight.

**
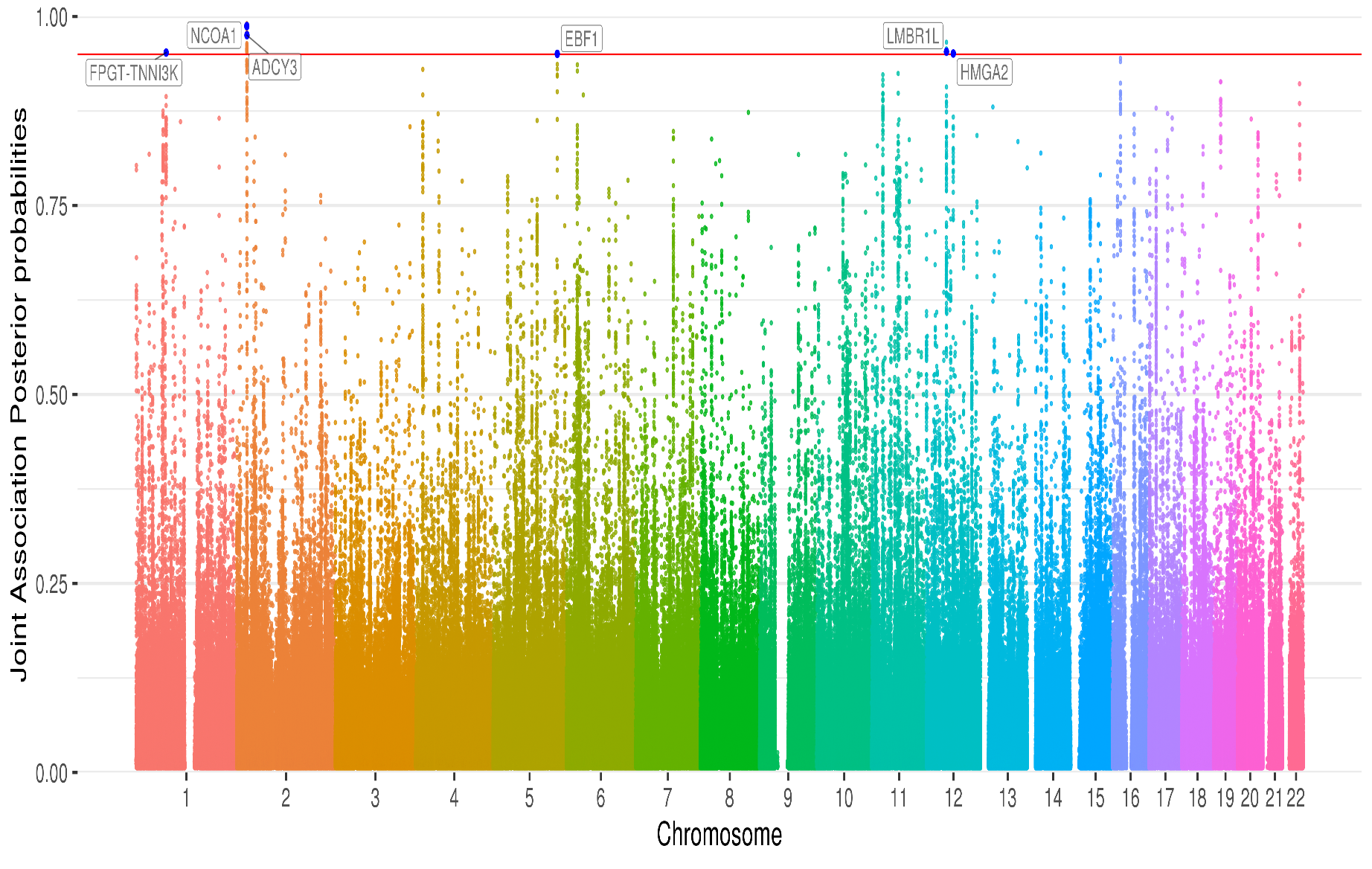
**

**Figure S3: Manhattan plot of joint association of variants with childhood body mass index and Transethnic birth weight.** The red line denotes the threshold of posterior probability (PP) at 0.95. The colored points under the red line are all common non-significant SNPs between childhood body mass index and transethnic birth weight that have PP < 0.95. The blue circles above or on the red line are all common significant SNPs found between childhood body mass index and transethnic birth weight that have PP ≥ 0.95. The text boxes contain the genes associated with the 6 pleiotropic genetic loci that was found jointly associated with both childhood body mass index and transethnic birth weight.

**
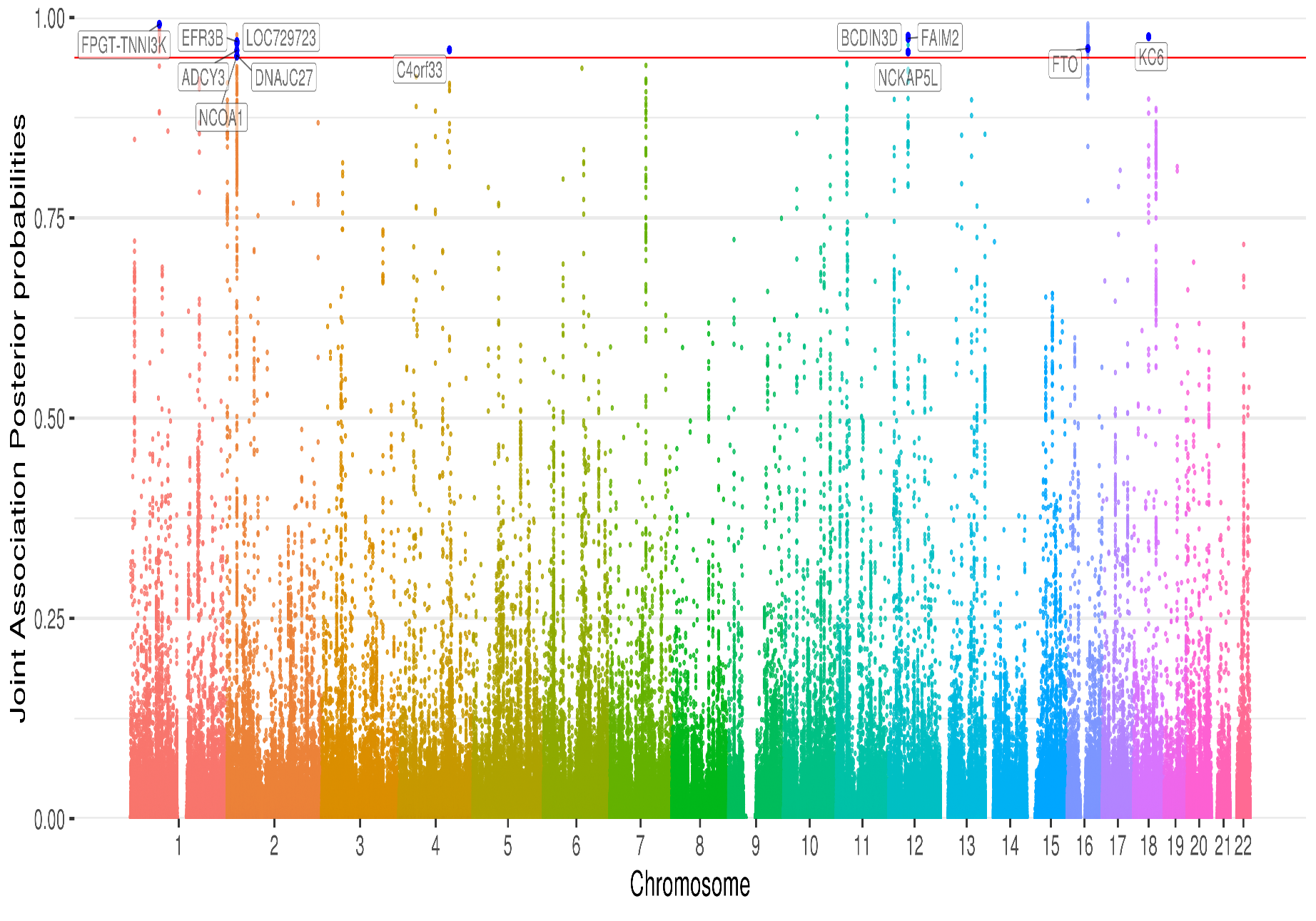
**

**Figure S4: Manhattan plot of joint association of variants with childhood Obesity and Transethnic birth weight.** The red line denotes the threshold of posterior probability (PP) at 0.95. The colored points under the red line are all common non-significant SNPs between childhood Obesity and transethnic birth weight that have PP < 0.95. The blue circles above or on the red line are all common significant SNPs found between childhood Obesity and transethnic birth weight that have PP ≥ 0.95. The text boxes contain the genes associated to the 9 pleiotropic genetic loci that was found jointly associated with both childhood Obesity and transethnic birth weight.

**
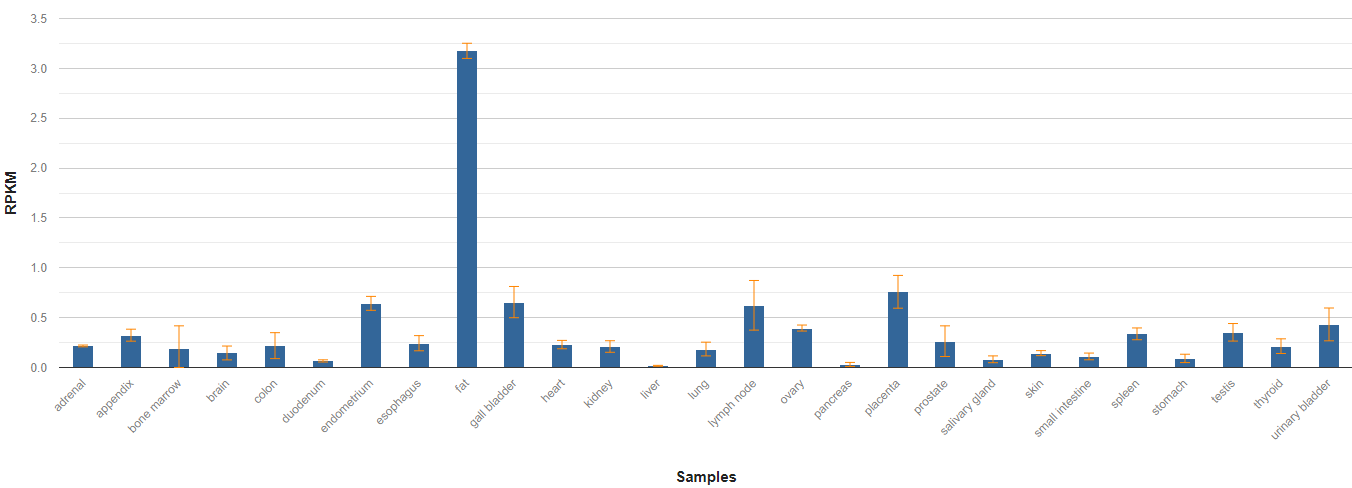
Figure S5: Expression of *EBF1* in different tissues** (source: GTEX).

**
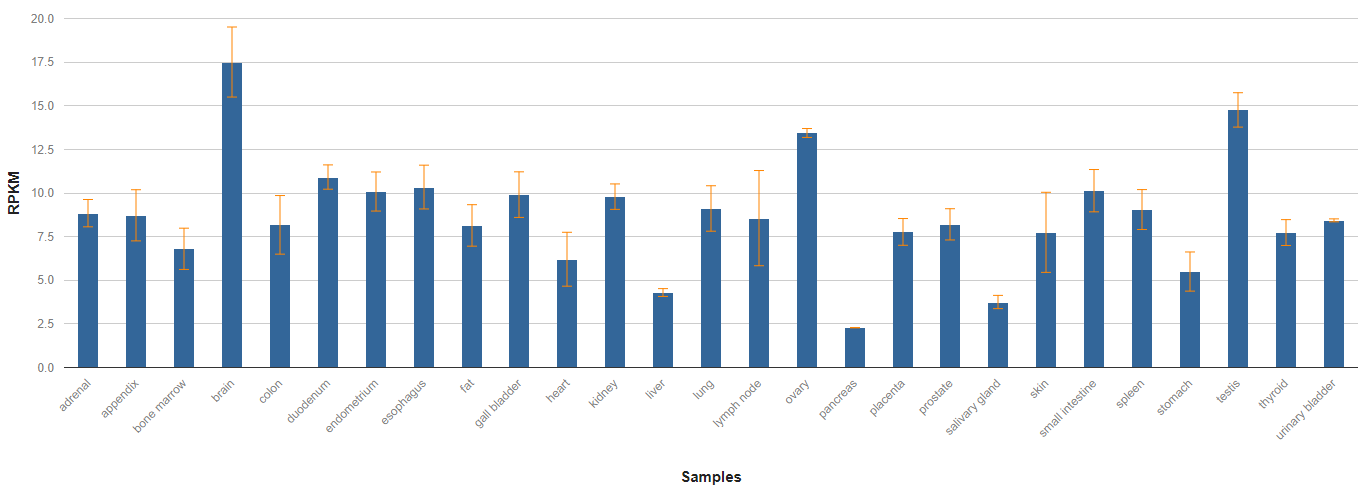
**

**Figure S6: Expression of *NCOA1* in different tissues** (source GTEX).
